# Bacteroides uniformis-generated hexadecanedioic acid ameliorates metabolic-associated fatty liver disease

**DOI:** 10.1080/19490976.2025.2508433

**Published:** 2025-05-25

**Authors:** Da-Ya Zhang, Da Li, Shi-Ju Chen, Li-Jun Zhang, Xu-Li Zhu, Fa-Di Chen, Chen Chen, Qi Wang, Yiping Du, Jian-Xin Xiong, Shi-Mei Huang, Xiao-Dong Zhang, Yan-Ting Lv, Fan Zeng, Run-Xiang Chen, Xianfeng Huang, Fengjiao Mao, Shuo Zhou, Qicen Yao, Yuliang Huang, Runyu Chen, Ying Mo, Yunqian Xie, Yue-Hong Jiang, Zhai Chen, Cui-Yi Mo, Jia-Jia Chen, Fei-Hu Bai

**Affiliations:** a The Second School of Clinical Medicine, Hainan Medical University, Haikou, China; b Department of Gastroenterology, The Second Affiliated Hospital of Hainan Medical University, Haikou, China; c Health Management Center, The Second Affiliated Hospital of Hainan Medical University, Haikou, China; d Department of Gastroenterology, Otog Front Banner People’s Hospital, Otog Front Banner, China; e Wuzhishan Center for Disease Control and Prevention, Wuzhishan, China; f Cardiovascular Surgery, The Second Affiliated Hospital of Hainan Medical University, Haikou, China; g Department of Gastroenterology, Hainan Second People’s Hospital, Wuzhishan, China; h Department of Rheumatology and Immunology, The Second Affiliated Hospital of Hainan Medical University, Haikou, China; i Department of Gastroenterology, The Second People’s Hospital of Ledong Li Autonomous County, Ledong Li Autonomous County, China; j Department of Gastroenterology, Dongfang People’s Hospital, Dongfang, China; k Department of Gastroenterology, Qionghai People’s Hospital, Qionghai, China; l Department of Gastroenterology, The Gastroenterology Clinical Medical Center of Hainan Province, Haikou, China

**Keywords:** Bacteroides uniformis, gut microbiota, hexadecanedioic acid, lipid metabolites, metabolic associated fatty liver disease, metabolomics

## Abstract

Gut microbiota exerts a pivotal influence on the development of Metabolic Associated Fatty Liver Disease (MAFLD), although the specific contributions of individual bacterial strains and their metabolites remain poorly defined. We conducted stool shotgun metagenomic sequencing and plasma untargeted metabolomics in a large prospective cohort comprising 120 MAFLD patients and 120 matched healthy controls. The mechanisms and microbial-derived metabolites involved in MAFLD were further investigated through multi-omics analyses *in vitro* and *in vivo*. Distinct differences were identified in both the microbial community structure and metabolomic profiles between MAFLD patients and healthy controls. Bacteroides uniformis (*B. uniformis*) was the most significantly depleted species in MAFLD and negatively correlated with hepatic steatosis and BMI. MAFLD was characterized by marked disruptions in fatty acid and amino acid metabolism. Combined analysis of metabolomic and metagenomic data achieved high diagnostic accuracy for MAFLD and hepatic steatosis severity (AUC = 0.93). Transplantation of fecal microbiota from MAFLD subjects into ABX mice led to the onset of MAFLD-like symptoms, whereas *B. uniformis* administration alleviate disease progression by inhibiting intestinal fat absorption, FFA from eWAT influx into liver via the gut-liver axis, and IRE1α-XBP1s-mediated flipogenesis and ferroptosis, as confirmed by hepatic transcriptomic and proteomic analyses. Hexadecanedioic acid (HDA), potentially identified as a key metabolite produced by *B. uniformis*, ameliorated MAFLD symptoms. Mechanistically, *B. uniformis*-derived HDA also inhibited fat absorption and transported, and entered the liver via the portal vein to suppress IRE1α-XBP1s-mediated flipogenesis and ferroptosis. B. uniformis and its potential putative metabolite HDA may contribute to MAFLD progression modulation, through regulation of the IRE1α-XBP1s axis. This study provides new insights into the gut-liver axis in MAFLD and offers promising therapeutic targets based on specific microbes and their metabolites.

## 1. Introduction

Metabolic Associated Fatty Liver Disease (MAFLD) presents a wide range of hepatic abnormalities, progressing from simple hepatic steatosis (HS) to more advanced stages such as steatohepatitis, fibrosis, cirrhosis, and even hepatocellular carcinoma.^1^ Affecting over 25% of adults worldwide,^2^ MAFLD has a prevalence of approximately 29.2% in China,^3^ making it a major contributor to chronic liver disease and an increasing public health concern.^4^ The underlying mechanisms of MAFLD remain unclear, and no approved pharmacological treatments are currently available. Further investigation into its pathogenesis and the development of novel therapeutic strategies are urgently needed.

Gut microbiota (GM) regulates host metabolism through the production of bioactive metabolites, playing a crucial role in the progression of MAFLD.^5,6^ Recent studies have demonstrated that GM promotes fatty liver disease by producing endogenous ethanol.^7^ The altered microbial composition has also been linked to shifts in circulating metabolites, such as amino acids, bile acids, and short-chain fatty acids (SCFA), all of which are implicated in metabolic dysfunction.^8^

However, comprehensive taxonomic and functional insights into GM in the context of MAFLD have been hindered by the limitations of *16S rRNA* gene sequencing technologies. Many studies have been descriptive and lacked mechanistic validation, limiting the ability to establish causality. The specific contributions of individual microbial species in the pathogenesis of MAFLD remain poorly defined. The interplay between specific bacteria and their metabolic products in driving disease progression has yet to be clearly defined.

Hence, a pivotal gut commensal strain along with its functional metabolite was identified as contributors to MAFLD. Pathogenic mechanisms involving these microbial factors were further elucidated through in vitro and in vivo experiments, integrating fecal metagenomic, hepatic transcriptomic, and proteomic analyses. These findings provide essential evidence of host-microbiota interactions and highlight potential microbial and metabolic targets for the management of MAFLD.

## 2. Materials and methods

### 2.1. Human studies

A non-interventional prospective study was designed to assess the GM composition and plasma metabolism in patients with MAFLD in China. Eligible participants, aged 18–65 years, were diagnosed with MAFLD according to international expert consensus guidelines.^9^ Detailed inclusion and exclusion criteria are provided in the Supplementary Materials. Ethical approval for this research was granted by both the Ethics Committee of the Second Affiliated Hospital of Hainan Medical University (LW2022223) and the Ethics Research Committee of Inner Mongolia Autonomous Region Otog Front Banner People’s Hospital (EC-20231214-1009). This study has been registered in Chinese Clinical Trial with registration number ChiCTR2500098170. All procedures adhered to the Declaration of Helsinki, with written informed consent obtained from each participant. Demographic information, including age, sex, BMI, lifestyle factors, comorbidities, and medical history, was collected through face-to-face interviews using standardized questionnaires. From April to July 2023, a total of 240 individuals were enrolled: 120 from Hainan (HN) and 120 from Inner Mongolia (NM), each contributing 60 MAFLD patients and 60 matched healthy controls (HC). Samples from all participants were collected for microbiome and plasma metabolomics analyses. Detailed procedures for anthropometric evaluation, sample processing, and clinical laboratory testing are provided in the Supplementary Materials.

### 2.2. Tool sample collection, DNA extraction and metagenomic shotgun sequencing

Extraction of bacterial DNA was carried out by Novogene Bioinformatics Technology (Beijing, China) utilizing the sodium dodecyl sulfate (SDS) method. The quality and concentration of extracted DNA were initially assessed with 1% agarose gel electrophoresis, and samples were adjusted to 1 ng/μL with sterile water. Gel electrophoresis was also employed to verify DNA integrity and detect any potential contaminants. A NanoPhotometer® (IMPLEN, CA, USA) was utilized to evaluate DNA purity by measuring the OD260/OD280 and OD260/OD230 ratios. Precise DNA quantification was performed with the Qubit® dsDNA Assay Kit on a Qubit® 2.0 Fluorometer (Life Technologies, Carlsbad, CA, USA). Shotgun metagenomic sequencing was carried out on the Illumina NovaSeq 6000 platform with paired-end reads (151 bp) and an average insert size of 350 bp, all processed at Novogene.

### 2.3. Serums LC/MS nontargeted metabolomics analysis

For serum metabolomics, 100 μL of sample was mixed with 400 μL methanol-based extraction solution containing isotopically labeled internal standards. After vortexing, samples were centrifuged at 12,000 rpm (13,800 × g, 8.6 cm radius) for 15 minutes at 4°C. The resulting supernatant was transferred to glass vials for analysis. Metabolomic profiling was performed on an ultra-high-performance liquid chromatography (UHPLC) system coupled with an Orbitrap Exploris 120 mass spectrometer operating in information-dependent acquisition (IDA) mode. During the run, continuous full-scan MS data collection was used to trigger MS/MS fragmentation. Metabolites were identified by comparison with a proprietary MS2 database (SHANGHAI BIOTREE BIOTECH Co., LTD), using an annotation confidence threshold of 0.3.^10^

### 2.4. Statistical analysis

All statistical analyses were performed utilizing SPSS (version 26; Chicago, Armonk, NY, USA). Categorical variables were presented as proportions, while continuous variables were reported as mean ± standard deviation (SD), median, range, or interquartile range (IQR), as appropriate. Categorical data were compared using the χ^2^ test. The unpaired t-test and Mann-Whitney U test were applied to analyze parametric and nonparametric continuous variables, respectively. Multivariate analysis was conducted using binary logistic regression. LEfSe analysis and ANOSIM^11,12^ were applied to compare microbial compositions between groups. Metabolite differences were evaluated using Student’s t-test, partial least squares discriminant analysis (PLS-DA), principal component analysis (PCA), and fold change analysis. Metabolomics data were processed and analyzed using the metaX R package,^13^ which facilitated data preprocessing, statistical evaluation, metabolite classification, and functional annotation. Spearman’s correlation analysis and random forest modeling were employed to assess associations between microbial taxa and metabolites. Statistical significance was determined using a two-sided p-value less than 0.05.

### 2.5. Predictive modeling of MAFLD diagnosis and severity by random forests

Microbial, metabolite, and clinical continuous variables were standardized utilizing Z-score normalization. A random forest model was constructed with the “randomForest” R package to integrate all variables. Ten-fold cross-validation, repeated five times using the “rfcv” function, was applied to assess the association between variable selection and the average classification error rate. Variables were ranked by Mean Decrease Accuracy, with the top 20 variables selected for subsequent modeling. Samples were randomly stratified into training and test sets in a 7:3 ratio. Model performance was assessed through calibration analyses, including calibration plots, to confirm predictive reliability.^14^

### 2.6. Bacteria culture and bacterial supernatant

*Bacteroides uniformis* (*B. uniformis*) (BNCC139204) was obtained from BeNa Culture Collection and cultured anaerobically at 37°C for 24 hours on Columbia blood agar plates using Mitsubishi anaeropacks and anaerobic culture bags, following established protocols.^15^ Bacterial proliferation was monitored by record in optical density at 600 nm with a microplate reader (Epoch2, BioTek Instruments, USA) and pH with a pH meter (FiveEasy Plus, METTLER TOLEDO, Shanghai, China) at 0, 3, 6, 9, 12, 15, and 24 hours.^16^ Following centrifugation at 4000 rpm for 5 minutes at 4°C, bacterial cells were harvested and resuspended in sterile anaerobic *PBS*. The suspension was adjusted to a final concentration of 2 × 10^8^ CFU/mL and kept at 4°C until further experimentation. The corresponding bacterial supernatants were divided into 1.5 mL EP *tubes* and subjected to concentration using a thermostatic mixer at 60°C for 10 hours.^17^

### 2.7. Animal experiments

All animal procedures were granted by the Ethics Committee of Hainan Medical University (HYLL-2023-453). Healthy male C57BL/6J mice (8 weeks old, specific-pathogen-free (SPF) grade) were obtained from SPF Biotechnology Co., Ltd. (Beijing, China). Animals were maintained in an SPF facility with a controlled environment (12-hour light/dark cycle, 24 ± 2°C, 40–60% humidity) and had free access to standard chow and sterile water. After one week of acclimatization, an antibiotic mixture was administered by oral gavage for 7 days to deplete GM and establish a pseudo-sterile model (ABx mice).^18^ The antibiotic regimen included neomycin sulfate (200 mg/kg), vancomycin (100 mg/kg), metronidazole (200 mg/kg), and ampicillin (200 mg/kg).

To evaluate the effects of fecal microbiota transplantation (FMT) and *B. uniformis* on MAFLD, 40 mice were randomized into five groups: normal chow diet with saline gavage (NC), high-fat diet with saline gavage (HFD), high-fat diet combined with fecal microbiota from MAFLD patients (MAF-FMT), high-fat diet with fecal microbiota from healthy individuals (HC-FMT), and high-fat diet with *B. uniformis* administration (Bu). Mice in the NC group were fed a standard chow diet, while all other groups received a HFD for 12 weeks to induce MAFLD. The HFD formulation supplied 60% of calories from fat, 20% from carbohydrates, and 20% from protein (Changzhou SYSE Bio-Tech. Co., Ltd.). In the Bu group, mice were orally gavaged every other day with 200 μL of saline containing *B. uniformis* at a concentration of 2 × 10^8^ CFU/mL. In the MAF-FMT and HC-FMT groups, mice received 200 μL of saline containing fecal bacterial suspension prepared by mixing 200 mg of feces with 2 mL of PBS, followed by centrifugation at 3000 rpm for 5 minutes at 4°C to collect the supernatant, and 200 μL of this solution was administered by gavage. NC and HFD received 200 μL sterile saline. Body weight and food consumption were recorded every two weeks. At the end of the 12-week intervention, following overnight fasting, mice were euthanized, and samples of feces, serum, liver, epididymal white adipose tissue (eWAT), ileum, and cecum were collected, immediately frozen in liquid nitrogen, and stored at −80°C for subsequent analyses.

To determine whether the anti-MAFLD effects of *B. uniformis* were mediated by its bioactive products rather than live bacteria,^16^ MAFLD mice were treated with live *B. uniformis* (Bu), heat-inactivated *B. uniformis* (Inact Bu; 100°C for 30 minutes), *B. uniformis* fermentation supernatant (Bu_SN), Columbia Blood Agar (CBA), or Hexadecanedioic Acid (HDA). Male C57BL/6J mice were randomized into nine groups: NC (*n* = 9), HFD (*n* = 8), PBS (*n* = 5), Bu (*n* = 6), Inact Bu (*n* = 5), Bu_SN (*n* = 5), CBA (*n* = 5), HDA-L (*n* = 5), and HDA-H (*n* = 5). The NC group was maintained on a normal chow diet, whereas all other groups received HFD for 12 weeks. Mice in the HDA-L and HDA-H groups were gavaged with 8 mg/0.2 mL and 16 mg/0.2 mL of HDA (Sigma-Aldrich, 505-54-4) every other day, respectively. The Bu and Inact Bu groups received 200 μL saline containing 2 × 10^8^ CFU/mL of B. uniformis. The Bu_SN, and CBA groups received 200 μL respective preparations. The NC and PBS groups were administered an equal volume of saline. Body weight and food consumption were recorded every two weeks. After 12 weeks, mice were executed and specimens collected as before.

Detailed methodologies for histopathological evaluation, biochemical assays, ROS detection, electron microscopy (TEM), transcriptomics, proteomics, fecal 16S rRNA analysis, quantitative real-time PCR (qRT-PCR), western blotting (WB), liquid chromatography – tandem mass spectrometry, and enzyme-linked immunosorbent assays (ELISA) are provided in the Supplementary Materials.

### 2.8. Cell culture and treatments

HepG2 were acquired from Wuhan Saibaikang (Shanghai) Biotechnology Co., Ltd. (Item No. iCell-h092, STR identified) and cultured in high-glucose DMEM enriched with 10% FBS and Penicillin-Streptomycin Solution. Cells were maintained at 37°C in a 5% CO₂ humidified incubator, with medium changes every two days as previously described.^19,20^ HepG2 cells were seeded into 6-well plates (Costar, Corning, NY, USA) at 3 × 10^5^ cells/well and cultured to over 80% confluence. Steatosis was induced by exposing cells to 1 mm free fatty acid (FFA) solution (palmitic acid and oleic acid, 1:2 molar ratio) for 24 hours.^21^ Cell viability was assessed utilizing a CCK-8 assay (Vazyme, Nanjing, China). Intracellular lipid accumulation and liver function were evaluated using Oil Red O staining (Modified Oil Red O Staining Kit) and triglyceride (TG) and aspartate aminotransferase (AST) quantification kits (Jiancheng, Nanjing, China). After confirming successful induction of steatosis, cells were treated for 24 hours with varying concentrations of HDA (160, 80, 40, 20, and 10 μg/mL). Following treatment, cells were collected to assess lipid accumulation and liver function. Based on protective effects against lipid accumulation and liver function, 80 μg/mL and 40 μg/mL HDA were selected for further experiments. Ferroptosis activators were subsequently added to evaluate the modulatory effects of HDA on ferroptosis. After treatment, cells were harvested for WB, qRT-PCR, ELISA, and TEM. All experiments were performed in triplicate.

To investigate the role of XBP1 in regulating hepatic ferroptosis and lipid metabolism, HepG2 cells were divided into four groups based on XBP1 expression modulation following PA/OA exposure: (1) control, (2) model, (3) negative control siRNA (si-NC), and (4) XBP1 knockdown (si-XBP1). After treatment, cells were collected for analysis of intracellular lipid accumulation, liver function, CCK-8, WB, qRT-PCR, ELISA, and TEM. All experiments were conducted in triplicate.

### 2.9. Statistical analyses of experiments in vivo and in vitro

Data are reported as mean ± SEM. For comparisons among there groups, one-way ANOVA followed by Tukey’s post hoc test was applied. Multiple testing adjustments were performed using the Benjamini-Hochberg false discovery rate, with significance set at an adjusted p-value ≤0.05. All statistical analyses were performed utilizing GraphPad Prism 10 (GraphPad Inc., San Diego, CA).

## 3. Results

### 3.1. Microbiome signatures of MAFLD patients

**Figure 1.**
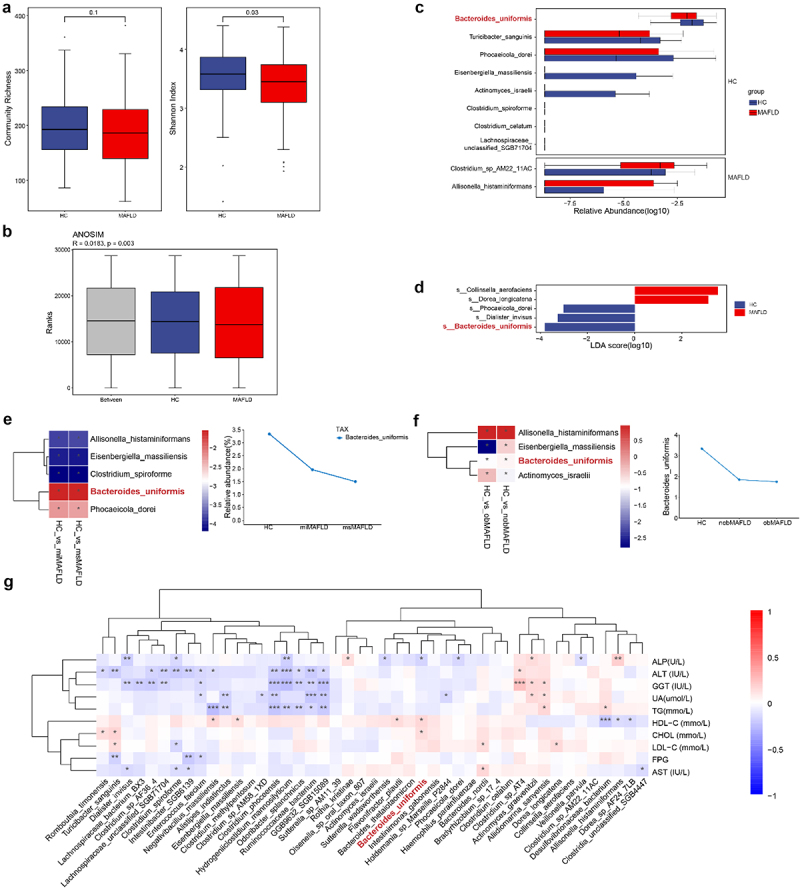
Diversity and compositional differences in the gut microbiome between HC and MAFLD patients. (a) Alpha diversity analysis comparing microbial community richness (observed species) and evenness (Shannon index) between the HC and MAFLD groups. (b) Beta diversity differences assessed using analysis of similarities (ANOSIM) based on metagenomic sequencing data at the species level, revealing significant separation between groups. (c) Species-level differential abundance analysis performed using the Wilcoxon rank-sum test, with results displayed as a bar plot highlighting statistically significant taxa. (d) Linear discriminant analysis (LDA) effect size (LEfSe) identifying microbial biomarkers that discriminate between HC and MAFLD groups based on phylogenetic and abundance differences. (e) Heatmap visualization of common characteristic bacterial species and their relative abundance in mild MAFLD (miMAFLD) and severe MAFLD (msMAFLD) compared to HC. (f) Heatmap comparison of shared bacterial signatures and their abundance profiles between non-obese MAFLD (nobMAFLD) and obese MAFLD (obMAFLD) versus HC. (g) Correlation network analysis illustrating associations between differentially abundant microbial species and key clinical parameters (e.g., liver enzymes, lipid profiles). The analysis included 120 healthy controls (HC) and 120 metabolic-associated fatty liver disease (MAFLD) patients, matched for age, sex, etc.

BMI, ALT, AST, GGT, FPG, uric acid (URIC), triglycerides (TG), cholesterol (CHOL), and LDL-C were lower in the HC group compared with the MAFLD group, while HDL-C levels were higher (Table 1). BMI, ALT, AST, FPG, URIC, and TG were independent risk factors for MAFLD (Table S1). Patients with MAFLD were stratified into two groups based on HS severity (mild, miMAFLD; moderate to severe, msMAFLD) and BMI (BMI <28 kg/m^2^, nobMAFLD; BMI ≥28 kg/m^2^, obMAFLD). ALT and AST were elevated in msMAFLD compared with miMAFLD group. Similarly, the msMAFLD group exhibited markedly elevated ALT and AST vs. the miMAFLD group(*p* < 0.01) (Table S2). Microbial diversity analysis revealed a reduced Shannon index in the MAFLD group relative to HC (Figure 1(a)), and β-diversity differed significantly between groups (ANOSIM; *p* = 0.003; Figure 1(b)). At the phylum level, MAFLD patients had decreased *Bacteroidetes* and increased *Actinobacteria* abundance (Figure S1(a,b). Genus-level analysis showed lower *Bacteroides* and higher *Lachnospiraceae_unclassified* in the MAFLD group (Figure S1(c,d). Species-level comparisons indicated that *B. uniformis* and *Phocaeicola dorei* were more abundant in HC than MAFLD (Figure 1(c), Figure S1(e)). Linear discriminant analysis effect size (LEfSe) identified *B. uniformis* as the most depleted species in MAFLD (Figure 1(d)), which was further confirmed by qPCR (Figure S1(f)). We further evaluate the relationship between microbiota and HS, BMI. No notable differences in either α-diversity or β-diversity were detected between the miMAFLD and msMAFLD groups (Figure S2(a–b)). Differentially expressed gene (DEGs) and LEfSe analyses identified *B. uniformis* as the most depleted species in both miMAFLD vs. HC and msMAFLD vs. HC comparisons (Figure S2(c,d), Figure 1(e)). The abundance of *B. uniformis* was further reduced in the msMAFLD group compared with the miMAFLD group (Figure 1(e)). Similarly, no significant differences in alpha or beta diversity were observed between the nobMAFLD vs. HC and obMAFLD vs. HC groups (Figure S2(e,f)), However, DEG and LEfSe analyses consistently identified *B. uniformis* as the most depleted species in both nobMAFLD and obMAFLD groups (Figure S2(g,h), Figure 1(f)), with lower abundance in the obMAFLD group vs. the nobMAFLD group (Figure 1(f)). Several microbial species showed correlations with clinical parameters of MAFLD, including ALT, AST, GGT, FPG, URIC, TG, CHOL, LDL-C, and HDL-C. *B. uniformis* was positively associated with HDL-C levels and negatively associated with ALP (Figure 1(g)). Functional predictions of microbial metabolic pathways indicated enrichment primarily in lipid and amino acid metabolism (Figure S3).

**Table 1.** Characteristics of the participants.

Indicator	HC (*n* = 120)	MAFLD (*n* = 120)	Test value	*p*
Age (year)	48.00(41–55.00)	52.0(44–56)	−1.898	0.058
Gender [male], n [%]	41(34.17%)	49(40.83%)	1.138	0.286
BMI (kg/cm^2^)	22.87(20.95–24.46)	26.48(23.49–29.29)	−7.194	<0.001
ALT (IU/L)	16.5(13–21)	25(17.25–37.75)	−6.365	<0.001
AST (IU/L)	16.5(13–21)	22.5(18–27)	−6.377	<0.001
GGT (IU/L)	20(14.25–28)	31(21–51.75)	−6.294	<0.001
FPG (mmol/L)	4.925(4.2875–5.185)	5.13(4.86–6.2625)	−4.787	<0.001
URIC (umol/L)	297.44 ± 57.02	369.44 ± 81.758	7.913	<0.001
TG (mmol/L)	1.000(0.7525–1.0875)	1.735(1.0125–1.7350)	−7.643	<0.001
CHOL (mmol/L)	5.0(4.3725–5.4425)	5.15(4.7375–6.000)	−2.924	0.003
HDL-C (mmol/L)	1.615(1.3125–1.9975)	1.200(1.000–1.430)	−6.929	<0.001
LDL-C (mmol/L)	2.905(2.415–3.205)	3.290(2.675–3.865)	−3.826	<0.001

### 3.2. Metabolomics alterations in the plasma of MAFLD patients

**Figure 2.**
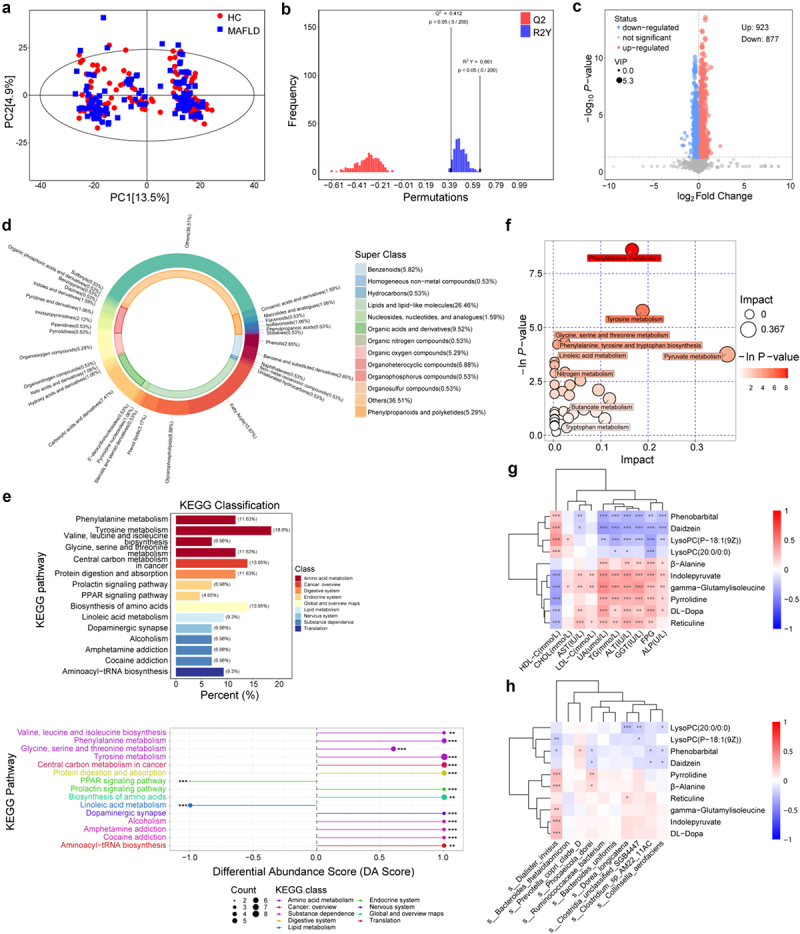
Serum metabolic profiling reveals distinct alterations in MAFLD patients compared to HC. (a) Principal component analysis (PCA) score plot illustrating the global metabolic differences between the HC and MAFLD groups, highlighting distinct clustering patterns. (b) Orthogonal partial least squares-discriminant analysis (OPLS-DA) permutation test (*n* = 200) demonstrating model validity, with the histogram showing the distribution of permuted R^2^ and Q^2^ values compared to the original model. (c) Volcano plot displaying the differential serum metabolites between HC and MAFLD, with each point representing a metabolite (x-axis: log₂ Fold change; y-axis: −log₁₀ p-value). Significantly altered metabolites (*p* < 0.05, Fold change > 1.5) are highlighted. (d) Hierarchical classification of differential metabolites based on the human metabolome database (HMDB), categorizing them into major chemical classes (e.g., lipids, amino acids, organic acids). (e) Kyoto Encyclopedia of genes and genomes (KEGG) pathway enrichment analysis of the differentially abundant metabolites, identifying key metabolic pathways dysregulated or upregulated in MAFLD. (f) Pathway impact analysis bubble plot integrating pathway enrichment (y-axis: −in p-value) and topological importance (x-axis: pathway impact score), with bubble size representing metabolite count. (g) Spearman correlation heatmap depicting associations between the top 10 significantly altered metabolites and clinical indices (e.g., liver enzymes, lipid profiles). (h) Microbial-metabolite correlation network revealing significant relationships (|r| > 0.5, *p* < 0.05) between key gut bacterial species (from Figure 1) and the top differential serum metabolites. The analysis included 120 healthy controls (HC) and 120 metabolic-associated fatty liver disease (MAFLD) patients, matched for age, sex, etc.

PCA was used to identify differential metabolites (Figure 2(a)). Validation of the PLS-DA model confirmed the absence of overfitting (Figure 2(b)). A volcano plot revealed 1,800 distinct metabolites differentiating the HC and MAFLD groups (Figure 2(c)). HMDB compound classification at the Superclass level indicated that most differential metabolites were lipids and lipid-like molecules, along with organic acids and their derivatives (Figure 2(d)). Among these, 189 metabolites with MS2 annotation were selected for further analysis. KEGG pathway analysis indicated notable disturbances in multiple metabolic pathways, such as tyrosine metabolism, amino acid biosynthesis, central carbon metabolism associated with cancer, phenylalanine metabolism, and glycine, serine, and threonine metabolism (Figure 2(e–f)). Differential Abundance Score (DA Score) analysis showed a marked upregulation of amino acid biosynthesis and metabolism (DA score = 1) and significant downregulation of linoleic acid metabolism and PPAR signaling pathway activity in MAFLD (Figure 2(e)). The top 10 differential metabolites, ranked by VIP values, included six upregulated metabolites: indolepyruvate, pyrrolidine, β-alanine, DL-dopa, reticuline, and gamma-glutamylisoleucine and 4 downregulated differential metabolites: LysoPC (*p*-18:1 (9Z)), Phenobarbital, Daidzein, and LysoPC (20:0/0:0), which exhibit strong correlations with clinical indicators in the context of MAFLD (Figure 2(g)). Correlation analysis was conducted between the top 10 microbial species identified by LEfSe (with relative abundance above 0.01%) and the top 10 differential metabolites (Figure 2(h)). Dorea longicatena demonstrated a strong negative correlation with LysoPC (20:0/0:0). LysoPC (20:0/0:0) and phenobarbital showed strong associations with multiple microbial species (Figure 2(h)). Further analysis revealed that plasma levels of LPC (18:1), LPC (18:2), LysoPC (O-18:0), and LysoPC (*p*-16:0) were significantly lower, while levels of L-tyrosine, 2-phenylacetamide, L-gulonic gamma-lactone, 4-hydroxycinnamic acid, and β-alanine were higher in subjects with msMAFLD compared with those with miMAFLD and HCs(*p* < 0.05)(Figure S4).

### 3.3. Joint analysis of microbiota and metabolomics

Correlation analysis was performed with thresholds of |rho| > 0.25 and p < 0.05 (Figure S5, Table S3). Several associations between microbial species and metabolites were identified. Intestinibacter_SGB6139 showed strong positive correlations with lipid and choline metabolites, including tetracosahexaenoic acid (corr = 0.41038) and ACar (13:1) (corr = 0.40584), as well as negative correlations with β-alanine (corr = −0.32911), L-carnitine (corr = −0.34130), and creatine (corr = −0.36336). Clostridium-spuAF364 demonstrated positive correlations with LPC (19:1) (corr = 0.43491) and LPC (17:1) (corr = 0.40258), and a negative correlation with β-alanine (corr = −0.30153). Clostridium-sap-AT4 was negatively correlated with LysoPC (O-18:0) (corr = −0.31021), while Negativibacillus massiliensis was negatively correlated with LPE (20:3) (corr = −0.32587). Turicibacillus sanguinis exhibited a negative correlation with cephagenin (corr = −0.34476). Among the differential metabolites, indolepyruvate (AUC = 0.78), daidzein (AUC = 0.74), and LysoPC (*p*-18:1 (9Z)) (AUC = 0.74) showed strong diagnostic performance. The combined metabolite prediction model achieved an AUC of 0.91, outperforming the microbial prediction model (AUC = 0.80). Integration of multi-omics data (microbiota + metabolites) further improved diagnostic accuracy, with an AUC of 0.93 (Figure S6).

### 3.4. *Colonization with gut microbiome from MAFLD patients was sufficient to induce MAFLD symptoms in mice, while* B. uniformis *was able to ameliorates MAFLD*

**Figure 3.**
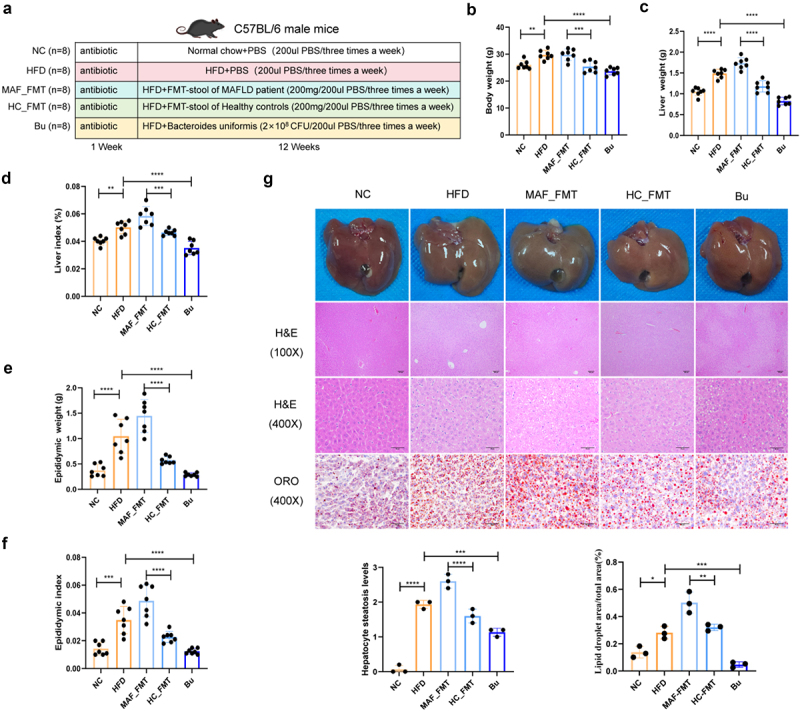
Effect of fecal microbiota transplantation (FMT) and Bacteroides uniformis supplementation on obesity and liver histopathology in HFD-induced MAFLD mice. (a) Experimental timeline illustrating the study design, including duration of antibiotic sustained cleansing, HFD feeding duration, FMT/B. uniformis intervention periods, *n* = 8 for each group. Pilot histology: At 11 weeks, a representative liver from each group underwent H&E/Oil red O/Masson staining, confirming both steatosis induction and treatment efficacy. (b) Body weight trajectories across experimental groups, demonstrating the impact of interventions on obesity progression, *n* = 7 for each group, *n* = 8 for each group. (c) Absolute liver weight measured at endpoint, showing significant differences between treatment groups and HFD controls, *n* = 7 for each group. (d) liver index calculated as (liver weight/body weight)×100%, reflecting hepatomegaly severity, *n* = 7 for each group. (e) Epididymal white adipose tissue (eWAT) mass, a marker of visceral adiposity, compared among experimental groups, *n* = 7 for each group. (f) Epididymal white adipose tissue (eWAT) index expressed as (eWAT weight/body weight)×100%, quantifying fat accumulation differences, *n* = 7 for each group. (g) Representative photomicrographs of liver sections stained with: H&E: revealing histopathological changes (steatosis, inflammation, ballooning); oil red O: quantifying neutral lipid deposition, *n* = 3 for each group. Statistical significance versus HFD group, *p < 0.05, **p < 0.01, ***p < 0.001(one-way ANOVA with post-hoc Dunnett’s test).

The role of the GM in MAFLD was evaluated by transplanting stool samples from HC, patients with MAFLD, and *B. uniformis* into C57BL/6 mice following antibiotic-mediated microbiota depletion (Figure 3(a), Figure S7). Metagenomic analysis and qPCR confirmed the successful colonization of *B. uniformis* (Figure S8). No significant differences in energy intake were observed between the HFD group and other HFD intervention groups (Table S4). After 12 weeks of intervention, mice in the HC-FMT and *B. uniformis* groups exhibited lower body weight, liver weight, liver index, epididymal fat weight, and eWAT index vs. the HFD group, whereas aggravate was found in the MAF-FMT group (Figure 3(b–f)). After 12 weeks of intervention, mice in Bu groups exhibited lower body weight, liver weight, liver index, eWAT weight, and eWAT index vs. the HFD group, whereas aggravate was found in the MAF-FMT group vs. HC-FMT (Figure 3(b–f)).

Similarly, eWAT pathology was elevated in the HFD vs. Bu group, while HC-FMT interventions significantly mitigated adipose tissue pathology compare to MAF-FMT group (Figure S9).

Histological analysis revealed that, compared with the NC group, the HFD group developed marked hepatic ballooning and steatosis, which was improved by Bu (Figure 3(g)). HC-FMT administration reduced these pathological changes compared to MAF-FMT group (Figure 3(g)).

Compared with the NC group, HFD exposure resulted in liver and kidney dysfunction, abnormal lipid metabolism(Figure S10), oxidative stress (OS), and elevated inflammatory responses (Figure S11), as indicated by increased serum levels of ALP, AST, ALT, CHOL, LDL-C, TG, LPS, IL-1β, and TNF-α, along with increased hepatic MDA, IL-1β, and TNF-α, and reduced serum HDL-C. These alterations were improved by Bu. Compared with MAF-FMT group, HC-FMT interventions mitigated these abnormalities.

Intestinal barrier function was evaluated by H&E staining. The NC group showed intact mucosal structure, whereas epithelial shedding was evident in the HFD group and was mitigated in the Bu group (Figure S12). Compared with MAF-FMT group, HC-FMT interventions mitigated these abnormalities (Figure S12).

Collectively, these findings demonstrate that colonization with GM from MAFLD patients induced MAFLD-like phenotypes in mice, whereas GM from HC or *B. uniformis* alleviated disease progression.

### 3.5. Fecal 16rRNA analysis in different groups

No significant differences were observed in Chao1, Goods_coverage, observed_otus, and Pielou_e indices among the groups (Figure S13(a)). Simpson index was elevated in the HFD group compared to the NC group, as well as in the MAF- FMT compared to the HC-FMT (Figure S13(a)). Principal coordinate analysis revealed clear separation across the five groups, with the HC-FMT and Bu groups showing distinct clustering away from the HFD group and trending toward the NC group (Figure S13(b)). Analysis of microbial composition at the phylum and genus levels revealed distinct differences between groups(Figure S13(c,d)). The relative abundance of *Actinobacteriota* increased progressively in the HFD and MAF-FMT groups compared with the NC group and was partially restored by HC-FMT and Bu intervention. At the genus level, *Ligilactobacillus* was reduced in the HFD and MAF-FMT groups and restored following HC-FMT or Bu treatment. KEGG pathway analysis (LEVEL 3) revealed that HC-FMT and Bu interventions upregulated genes involved in human disease pathways, biological systems, genetic information processing, and lipid and amino acid metabolism compared with the HFD group (Figure S13(e,f)), suggesting potential involvement in disease improvement.

Twelve genera, including P*revotellaceae_UCG-001* and *Duncaniella*, showed significant alterations in abundance in the HFD group and were restored following Bu intervention. These genera displayed negative correlations with liver enzymes, lipid levels, BMI, and markers of inflammation and ROS, as shown in the heatmap (Figure S13(g)). Network analysis indicated that Bu enhanced microbial interactions, with strong associations observed between genera such as *Proteus* and *Absiella* (Figure S13(h)).

We also focus on targeted metagenomic sequencing of donor microbiota from the HC-FMT group showing anti-MAFLD effects, with particular on Bacterial species. Metagenomic analysis revealed two additional fatty acid-producing Bacteroides species (B. thetaiotaomicron and B. nordii) enriched in healthy controls, showing cooperative interactions with B. uniformis (Figure S14(a)). Besides, fecal 16srRNA revealed HC-FMT recipients exhibited enriched probiotic taxa (Ligilactobacillus and Lactobacillales) compared to MAF-FMT mice (Figure S14(b)).

### 3.6. B.Uniformis suppresses intestinal fat absorption and free fatty acids (FFA) from eWAT influx into liver via the gut-liver axis

**Figure 4.**
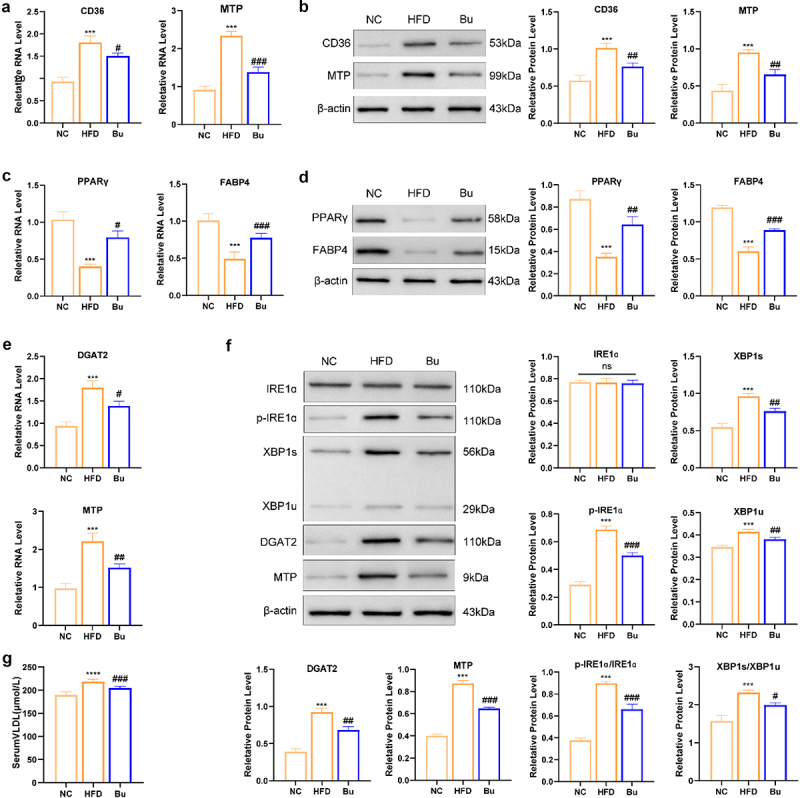
B. uniformis inhibited fat absorption and transported, and IRE1α-XBP1-mediated flipogenesis. (a) Ileum mRNA expression profiles of fat absorption and transported-related genes (CD36, MTP) (*n* = 3 for each group). (b) Quantitative analysis of WesternBlot banding plots of CD36, MTP proteins in the ileum of mice and the gray value of protein expression levels (*n* = 3 for each group). (c) White adipose tissue (eWAT) mRNA expression profiles of fatty acid handling-related genes (PPARγand FABP4) (*n* = 3 for each group). (d) Quantitative analysis of WesternBlot banding plots of PPARγand FABP4 proteins in the eWAT of mice and the gray value of protein expression levels (*n* = 3 for each group). (e) Liver mRNA expression profiles of flipogenesis-related genes (DGAT2 and MTP) (*n* = 3 for each group). (f) Quantitative analysis of WesternBlot banding plots of p-IRE1α, p-IRE1α/IRE1α ratio, XBP1s, XBP1u, XBP1s/XBP1u ratio, DGAT2, and MTP proteins in the liver of mice and the gray value of protein expression levels (*n* = 3 for each group). (g) Serum VLDL, *n* = 8 for each group. Statistical significance: compared with the NC group, *p < 0.05, **p < 0.01, ***p < 0.001. Compared with the HFD group, #*p* < 0.05, # #*p* < 0.01, # # # *p* < 0.001.

Analysis by qPCR and WB suggested that HFD tended to increase expression levels of the fatty acid transporter CD36 and the chylomicron synthesis-related factor MTP in ileal epithelial cells relative to NC group (Figure 4(a–b)). In eWAT, HFD feeding appeared to moderately reduce expression of PPARγ, an important regulator of adipocyte differentiation, along with FABP4, a protein involved in fatty acid handling (Figure 4(c–d)). Interestingly, administration of *B. uniformis* showed potential to partially counteract these HFD-associated changes such as CD36, MTP, PPARγand FABP4 expression patterns.

### 3.7. Liver transcriptomic and proteomic analysis revealed B.Uniformis might suppresses XBP1-mediated flipogenesis and ferroptosis

RNA sequencing of liver tissues was performed to explore the mechanism by which *B. uniformis* alleviates hepatic steatosis (HS) in MAFLD. A total of 494 DEGs were identified in the HFD group vs. the NC group, including 220 upregulated and 274 downregulated genes(Figure S15(a)). Compared with the HFD group, the Bu group showed 3,588 DEGs, with 2,067 upregulated and 1,521 downregulated(Figure S15(b)). KEGG analysis and volcano plots illustrated the top 20 altered genes (Figure S16(a,b)). Among these, 199 genes showed reversed expression patterns between the Bu, HFD, and NC groups. Cytoscape analysis identified three gene clusters: Gm11808, Rpl31, Rpsa, Mrpl13, Rpl32, and Mrto4, Rps27a; Srsf6, Luc712, Rbm25, Bclaf1, and Snrpb2; and Gstk1, Ube2d2a, Pex5, and Ehhadh. Ehhadh participates in the peroxisomal fatty acid β-oxidation pathway(Figure S17).

Hepatic proteomic analysis was performed to further investigate the underlying mechanisms. The top 20 upregulated and downregulated KEGG pathways are presented in Figure S18(a,b). Compared to the NC group, the HFD group showed enrichment of pathways associated with ferroptosis, chemokine signaling, NOD-like receptor signaling, lipid metabolism, and atherosclerosis, with XBP1 identified as a key regulatory molecule (Figure S18(a)). Following *B. uniformis* intervention, enriched pathways included autophagy and cAMP signaling, with core genes such as PSMD10, Gde1, Apoh, and Fam193a (Figure S18(b)). Sterol metabolism emerged as the most affected pathway, with fatty acid synthase (FASN) and hydroxysteroid 17-β dehydrogenase 7 (HSD17B7) identified as key genes (Figure S19(a)). DEPs regulated by *B. uniformis* across the three groups were primarily enriched in pathways linkes to energy, lipid, amino acid, and OS (Figure S19(b)).

Ferroptosis emerged as one of the top ten downregulated pathways in the Bu group vs. the HFD group(Figure S20(a)). Analysis of ferroptosis- related gene expression showed upregulation of Fth1, Ftl1, GPX4 and Trf (Figure S20(b)), along with downregulation of Lpcat3, a key promoter of lipid peroxidation. The antioxidant gene GPX4 was upregulated, suggesting enhanced defense against oxidative damage. Protein-protein interaction network analysis of the transcriptome indicated that XBP1 occupies a central position in the regulation of ferroptosis in MAFLD (Figure S20(c)). Integrated transcriptomic and proteomic analysis of the Bu and HFD groups revealed 45 upregulated and 19 downregulated molecules, primarily associated with the PPAR signaling pathway, oxidative phosphorylation, and metabolic pathways. PPI network analysis further suggested XBP1 as a critical regulator (Figure S20(d)).

#### 3.7.1. B. uniformis suppresses IRE1α-XBP1-mediated flipogenesis

qPCR analysis demonstrated that the mRNA expression levels of MTP and DGAT2 in liver tissues were substantially higher in the HFD group compared with the NC group, and *B. Uniformis* administration effectively downregulated their expression in HFD-fed mice (Figure 4(e)). Consistent with these findings, WB revealed that the protein expression levels of p-IRE1α, p-IRE1α/IRE1α ratio, XBP1s, XBP1u, XBP1s/XBP1u ratio, DGAT2, and MTP in liver tissues were significantly elevated in the HFD group compared with the NC group (Figure 4(f)). Notably, B. Uniformis intervention markedly reduced the expression of these indicators compared to the HFD group. Besides, Serum VLDL was substantially higher in the HFD group compared with the NC group, and *B. Uniformis* administration effectively downregulated their expression in HFD-fed mice (Figure 4(g)).

#### B. uniformis suppresses IRE1α-XBP1-Hrd1 mediated ferroptosis via Nrf2/SLC7A11/GPX4 pathway

**Figure 5.**
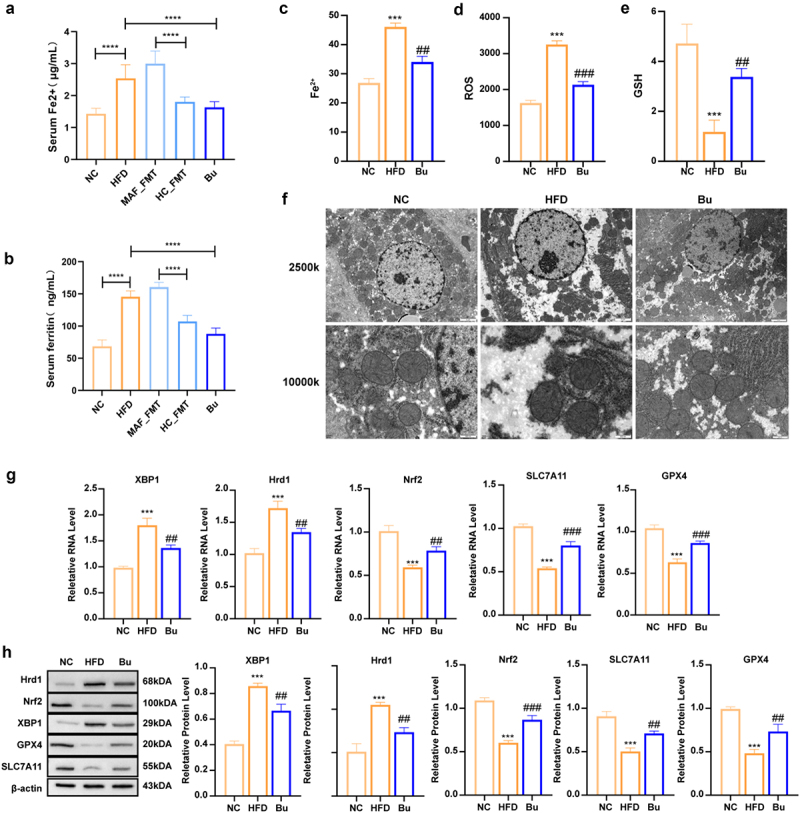
Bu attenuates ferroptosis and ameliorates MAFLD progression in mice. (a) Serum Fe^2^+ of mice in different groups(*n* = 8 for each group). (b) Serum ferritin of mice in different groups(*n* = 8 for each group). (c) Liver Fe^2^+ of mice in different groups(*n* = 3 for each group). (d) Reactive oxygen species (ROS) levels in liver tissues in different groups(*n* = 3 for each group). (e) Liver glutathione (GSH) levels of mice in different groups(*n* = 3 for each group). (f) Representative transmission electron microscopy (TEM) images of liver ultrastructure (*n* = 3 for each group). (g) Liver mRNA expression profiles of ferroptosis-related genes (XBP1, Nrf2, Hrd1, SLC7A11, GPX4) (*n* = 3 for each group). (h) quantitative analysis of WesternBlot banding plots of XBP1, Nrf2, Hrd1, SLC7A11, and GPX4 proteins in the liver of mice and the gray value of protein expression levels (*n* = 3 for each group). (a-b) Statistical significance: (*p < 0.05, **p < 0.01, ***p < 0.001, and ****p < 0.0001, two subgroups were compared separately: NC vs HFD vs Bu, MAF_FMT vs HC_FMT). (c-h)Statistical significance: compared with the NC group, *p < 0.05, **p < 0.01, ***p < 0.001. Compared with the HFD group, #*p* < 0.05, # #*p* < 0.01, # # # *p* < 0.001.

Compared to HC, serum Fe^2 +^ and ferritin levels were elevated in MAFLD patients and positively correlated with HS (Figure S21(a,b)). Corresponding increases were observed in the HFD group and were more pronounced in the MAF-FMT group, while both HC-FMT and Bu interventions significantly reduced these indicators (Figure 5(a–b)). Compared with the HFD group, the Bu group exhibited reduced hepatic Fe^2 +^ levels, decreased ROS production, increased GSH levels (Figure 5(c–e)), and fewer ferroptosis-associated mitochondrial abnormalities (Figure 5(f)). Validation of this pathway was confirmed by qRT-PCR and WB analyses in the MAFLD mouse model (Figure 5(g–h)).

### 3.8. *HDA is identified as the functional metabolite generated from* B. uniformis

**Figure 6.**
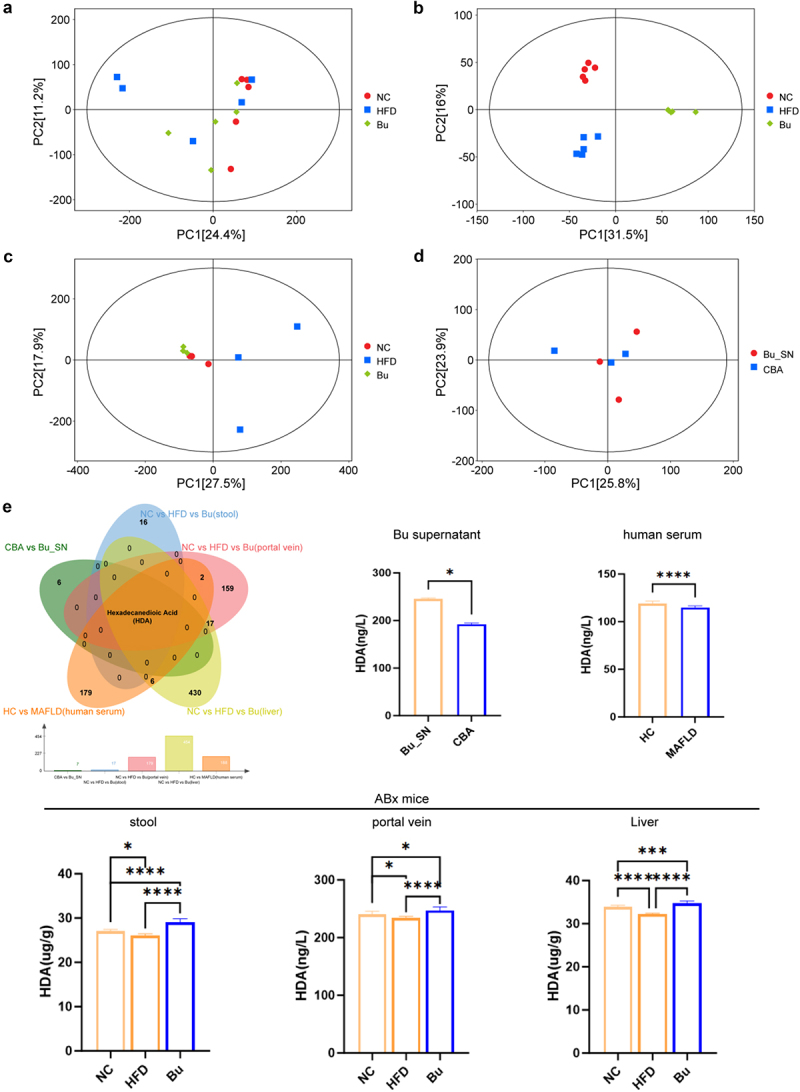
Metabolomics identifies HDA as the key bioactive metabolite mediating the therapeutic effects of Bu in MAFLD mice. (a) Principal component analysis (PCA) of stool metabolomic profiles across experimental groups, *n* = 5 for each group. (b) PCA of portal vein plasma metabolites, *n* = 5 for each group. (c) Hepatic metabolic profiling by PCA, *n* = 3 for each group. (d) PCA of Bu supernatant metabolites, *n* = 3 for each group. (e) Intersection of metabolites and HDA quantification from different sample sources. Venn diagram illustrating overlapping metabolites among different biological matrices. HDA level in the portal vein, stool, and liver of B. uniform-gavaged and PBS-gavaged germ-free mice. HDA level between HC and MAFLD groups. HDA level between B. uniform and Columbia blood agar groups. (*p < 0.05, **p < 0.01, ***p < 0.001, and ****p < 0.0001).

To investigate the metabolic mechanisms by which *B. uniformis* influences MAFLD, untargeted metabolomics was conducted on stool (Figure 6(a)), portal vein serum (Figure 6(b)), and liver tissues (Figure 6(c)) from ABX mice gavaged with *B. uniformis* for three months. In parallel, untargeted metabolomics of *B. uniformis* culture supernatant identified 218 significantly elevated metabolites (Figure 6(d)). Serum metabolomics data from MAFLD patients in the cohort were further analyzed. Cross-comparison of the five datasets using a Venn diagram indicated that HDA may serve as a *B. uniformis*-derived metabolite contributing to MAFLD progression (Figure 6(e)).

KEGG pathway analysis of supernatant metabolites revealed that *B. uniformis* significantly impacted metabolic processes, particularly fatty acid biosynthesis. Genomic analysis of *B. uniformis* identified the presence of a gene (EC:3.1.2.14, FASN) involved in the HDA synthesis pathway (hexadecanoyl-[ACP] → hexadecanoic acid) (Figure S22).

These results indicate that HDA is closely linked to *B. uniformis* and may represent a key metabolite in the pathogenesis of MAFLD.

### 3.9. HDA suppresses flipogenesis and ferroptosis in vitro

**Figure 7.**
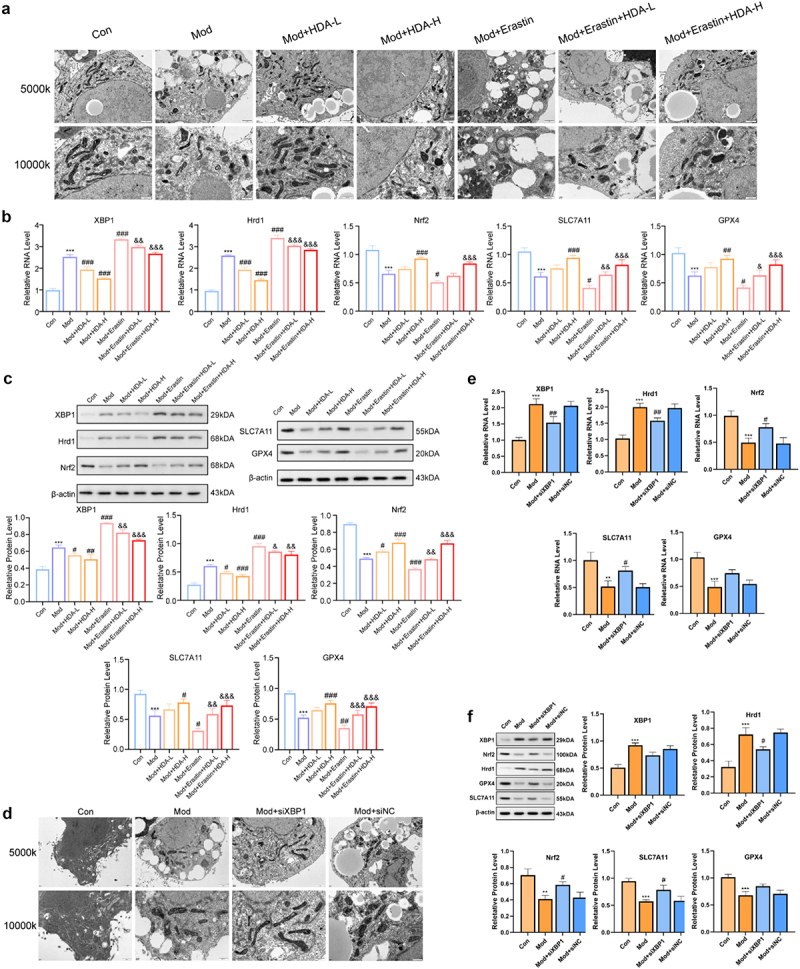
HDA attenuates ferroptosis in MAFLD through XBP1 regulation. (a) Representative transmission electron micrographs of hepatic ultrastructure in HepG2 MAFLD models following HDA treatment(*n* = 3 for each group). (b) qRT-PCR analysis of ferroptosis-related genes (XBP1, Nrf2, Hrd1, SLC7A11, GPX4) in HDA-treated HepG2 MAFLD cells(*n* = 3 for each group). (c) Quantitative Western blot analysis of corresponding protein expression levels (normalized band intensities shown)(*n* = 3 for each group). (d) liver electron microscopy of mice in HepG2 MAFLD cell model with knockdown of XBP1 added(*n* = 3 for each group). (e) Effects of HDA on mRNA expression levels of XBP1, Nrf2, Hrd1, SLC7A11, GPX4 in HepG2 MAFLD cell model with knockdown of XBP1 added(*n* = 3 for each group). (f) quantitative analysis of WesternBlot banding plots of XBP1, Nrf2, Hrd1, SLC7A11, and GPX4 proteins in HepG2 MAFLD cell model with knockdown of XBP1 added(*n* = 3 for each group). Statistical significance: (a-c)***p < 0.001, ****p < 0.0001 vs control; ##*p* < 0.01, ####*p* < 0.0001 vs model; &*p* < 0.05, &&*p* < 0.01, &&&&*p* < 0.0001 vs model+Erastin. (D-F) ***p < 0.001 vs control; ##*p* < 0.01 vs model.

Cytotoxicity testing showed that HDA did not induce significant toxicity in HepG2 cells at concentrations below 320 μg/mL (Figure S23(a)). Treatment with 80 μg/mL and 40 μg/mL HDA significantly reduced intracellular ALT and TG (Figure S23(b,c)), align with the results of Oil Red O staining(Figure S23(d)). Based on these findings, 80 μg/mL and 40 μg/mL HDA were selected to evaluate the effects on ferroptosis in the HepG2 steatosis model, with or without the ferroptosis inducer Erastin. Following HDA treatment, intracellular HDA levels increased, indicating cellular uptake and activity(Figure S24(a)). Compared with the Control group, the Mod group exhibited elevated MDA, ROS, Fe^2 +^ levels, decreased GSH (Figure S24(b,e)), along with mitochondrial morphological changes characteristic of ferroptosis (Figure 7(a)). These alterations were more pronounced in the Mod+Erastin group. HDA treatment reversed these changes, with 80 μg/mL showing stronger effects. Consistent with the mechanisms observed in MAFLD mice, RT-qPCR and western blot analyses confirmed that HDA downregulated XBP1 and Hrd1 expression while upregulating Nrf2, SLC7A11, and GPX4 in the HepG2 steatosis model, thereby inhibiting ferroptosis (Figure 7(b–c)).

### 3.10. Effect of knockdown of XBP1 on lipid metabolism and ferroptosis in vitro

XBP1 knockdown was successfully achieved in the HepG2 MAFLD cell model, as confirmed by reduced XBP1 mRNA expression following plasmid interference (Figure S25). Cells transfected with si-XBP1–2, which exhibited the most significant reduction in XBP1 expression, were selected for further analysis. Compared with the empty plasmid control group, XBP1 knockdown significantly reduced intracellular TG, ALT, Fe^2 +^, ROS, and MDA levels (Figure S26), while Oil Red O staining indicated a decrease in lipid accumulation (Figure S27). In contrast, GSH levels and cell viability increased following XBP1 knockdown (Figure S26). Mitochondrial morphology also improved in the XBP1 knockdown group, characterized by reduced volume, increased membrane density, and decreased cristae structure, indicating attenuation of ferroptosis-related damage (Figure 7(d)). XBP1 knockdown led to reduced mRNA and protein expression of XBP1 and Hrd1 (*p* < 0.05), while Nrf2, SLC7A11, and GPX4 expression levels were significantly upregulated at the mRNA and protein levels vs. the empty plasmid control group (*p* < 0.05)(Figure 7(e–f)). In conclusion, XBP1 is central in linking *B. uniformis*-generated HDA and SLC7A11/GPX4 ferroptosis signaling pathway.

### 3.11. HDA suppresses IRE1α-XBP1-Hrd1 mediated ferroptosis via Nrf2/SLC7A11/GPX4 pathway in vivo

To determine whether the anti-MAFLD effects of *B. uniformis* are mediated by its bioactive metabolites rather than the bacteria themselves, MAFLD mice were treated with live *B. uniformis*, heat-inactivated *B. uniformis* (100°C for 30 minutes), fermentation products from Columbia Blood Agar (Bu_SN), CBA, and HDA (Figure S28A).

**Figure 8.**
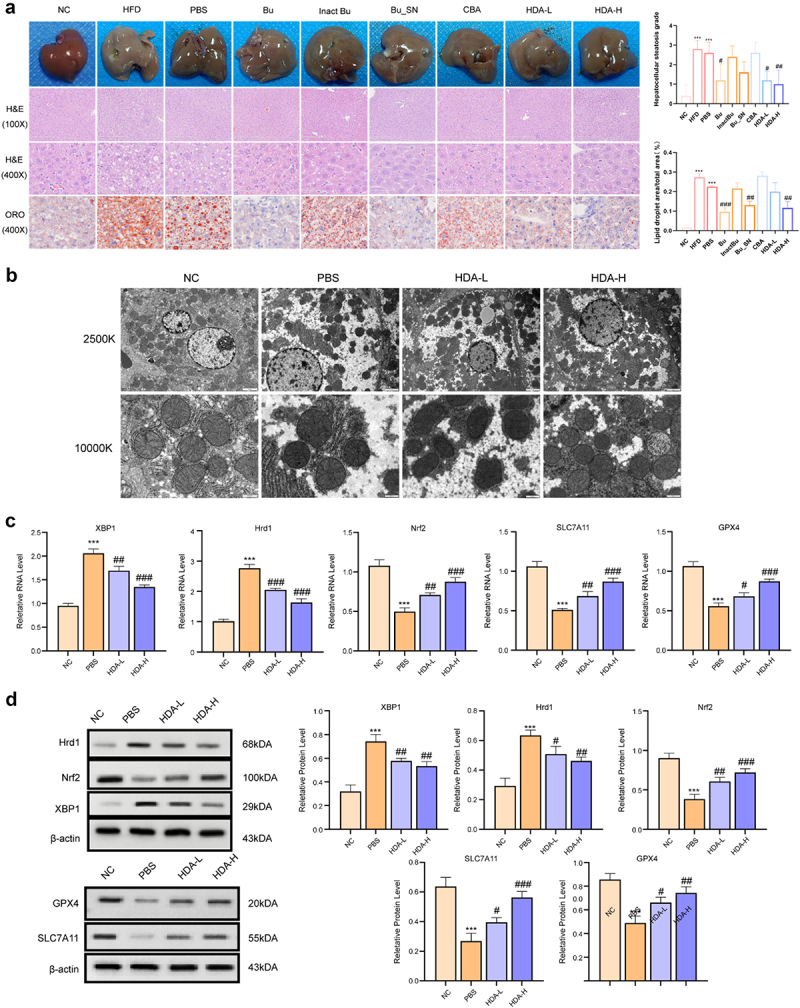
HDA attenuates mitochondrial ferroptosis in MAFLD mouse hepatic cells during MAFLD progression. (a) Histopathological evaluation of liver sections by hematoxylin-eosin (H&E) and lipid deposition by oil red O staining across experimental cohorts(*n* = 3 for each group). (b) Ultrastructural examination of hepatocytes via transmission electron microscopy(*n* = 3 for each group). (c) Relative mRNA abundance of ferroptosis-associated genes (XBP1, NRF2, HRD1, SLC7A11, GPX4) in hepatic tissues(*n* = 3 for each group). (d) Quantitative immunoblot analysis of corresponding protein expression, with band intensities normalized to reference controls(*n* = 3 for each group). Statistical analyses: *p < 0.05, **p < 0.01, ***p < 0.001 versus normal control (NC) group; #*p* < 0.05, ##*p* < 0.01, ###*p* < 0.001 versus phosphate buffer saline(PBS) group.

No significant differences in energy intake were observed between the HFD group and intervention groups (Table S5). However, body weight, epididymal fat index, liver weight, and liver index were elevated in the HFD and PBS groups compared to NC, while these parameters were notably reduced after treatment with live *B. uniformis*, Bu_SN, and HDA (Figure S28(b-e)). No improvements were observed with heat-inactivated *B. uniformis*, CBA, or PBS (Figure S28(b-e)). Histological analysis revealed that steatosis scores and lipid droplet area ratios were reduced following intervention with live *B. uniformis*, Bu_SN, and HDA, with no improvements detected in the inactivated *B. uniformis*, CBA, or PBS groups (Figure 8(a)). AST, ALT, TC, TG, LDL-C, and HDL-C were also improved after treatment with live *B. uniformis*, Bu_SN, and HDA (Figure S29). These findings indicate that the anti-MAFLD impact of *B. uniformis* are primarily mediated by its active metabolite HDA rather than the bacteria themselves. Besides, 135.9ng/L HDA is circulating in systemic circulation with a dose of 16 mg/0.2 ml (Figure S30).

Consistent with the protective mechanism of *B. uniformis* in MAFLD mice, HFD increased serum LPS, MDA, ROS, Fe^2 +^, IL-1β, and TNF-α levels, while reducing GSH levels. These alterations were reversed following HDA treatment, with the most pronounced effects observed at 16 mg/0.2 mL (Figure S31). HDA treatment also improved mitochondrial morphology, reducing ferroptosis-related structural damage (Figure 8(b)). Evaluation of the intestinal barrier revealed that mucosal integrity was maintained in the NC group, while the HFD group exhibited mucosal shedding, which was alleviated by HDA intervention (Figure S32). RT-qPCR and western blot analyses confirmed that HDA downregulated hepatic XBP1 and Hrd1 expression while upregulating Nrf2, SLC7A11, and GPX4, contributing to ferroptosis inhibition in MAFLD mice (Figure 8(c–d)).

### 3.12. HDA suppresses intestinal fat absorption, FFA from eWAT influx into liver via the gut-liver axis and liver XBP1-mediated flipogenesis

**Figure 9.**
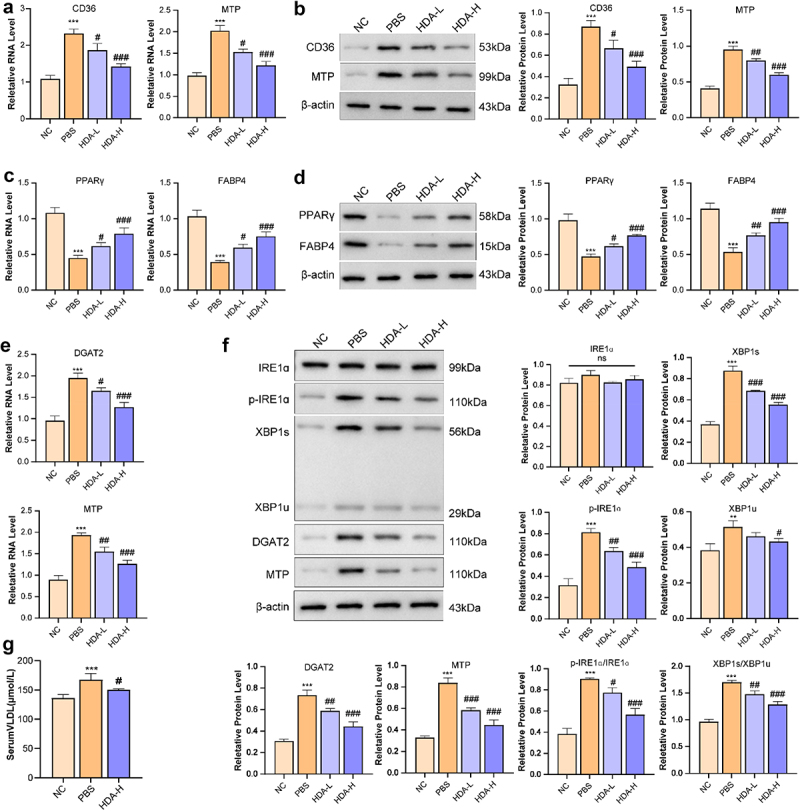
HDA inhibited fat absorption and transported, and IRE1α-XBP1-mediated flipogenesis. (a) ileum mRNA expression profiles of fat absorption and transported-related genes (CD36, MTP) (*n* = 3 for each group). (b) quantitative analysis of WesternBlot banding plots of CD36, MTP proteins in the ileum of mice and the gray value of protein expression levels (*n* = 3 for each group). (c) white adipose tissue (eWAT) mRNA expression profiles of fatty acid handling-related genes (PPARγand FABP4) (*n* = 3 for each group). (d) quantitative analysis of WesternBlot banding plots of PPARγand FABP4 proteins in the eWAT of mice and the gray value of protein expression levels (*n* = 3 for each group). (e) liver mRNA expression profiles of flipogenesis-related genes (DGAT2 and MTP) (*n* = 3 for each group). (f) quantitative analysis of WesternBlot banding plots of p-IRE1α, p-IRE1α/IRE1α ratio, XBP1s, XBP1u, XBP1s/XBP1u ratio, DGAT2, and MTP proteins in the liver of mice and the gray value of protein expression levels (*n* = 3 for each group). (g) serum VLDL. Statistical significance: compared with the NC group, *p < 0.05, **p < 0.01, ***p < 0.001. Compared with the PBS group, #*p* < 0.05, # #*p* < 0.01, # # # *p* < 0.001.

Analysis by qPCR and Western blot suggested that PBS group tended to increase expression levels of the fatty acid transporter CD36 and the chylomicron synthesis-related factor MTP in ileal epithelial cells relative to NC (Figure 9(a–b)). In eWAT, PBS group appeared to moderately reduce expression of PPARγ, an important regulator of adipocyte differentiation, along with FABP4, a protein involved in fatty acid handling (Figure 9(c–d)). Interestingly, administration of HDA showed potential to partially counteract these HFD-associated genes and proteins expression patterns changes such as CD36, MTP, PPARγand FABP4.

qPCR analysis demonstrated that the mRNA expression levels of MTP and DGAT2 in liver tissues were substantially higher in the PBS model group than in NC, and HDA administration effectively downregulated their expression in PBS model group (Figure 9(e)). WB analysis revealed that the protein expression levels of p-IRE1α, p-IRE1α/IRE1α ratio, XBP1s, XBP1u, XBP1s/XBP1u ratio, DGAT2, and MTP in liver tissues were significantly elevated in the PBS model group compared with the NC group (Figure 9(f)). Consistent with these findings, HDA intervention markedly reduced the expression of these indicators compared to the PBS model group. Besides, Serum VLDL was substantially higher in the PBS group compared with the NC group, and HDA effectively downregulated their expression in HFD-fed mice (Figure 9(g)).

## 4. Discussion

Recent studies have highlighted the strong association between MAFLD and metabolic dysfunction. Consistent with these findings, BMI, ALT, AST, FPG, URIC, and TG were identified as independent risk factors for MAFLD, supporting a disrupted metabolic state. Among these, ALT and AST may serve as potential biomarkers for assessing the severity of hepatic steatosis in MAFLD. Previous research has demonstrated that URIC contributes to MAFLD progression by promoting insulin resistance, enhancing mitochondrial and endoplasmic reticulum OS, triggering inflammatory responses, and facilitating fructose metabolism.^22,23^

The GM is essential in the pathogenesis of MAFLD by producing bioactive metabolites that interact with host metabolic processes.^24^ Intestinal dysbiosis is commonly observed in patients with MAFLD, characterized by a reduction in beneficial genera, such as *Bifidobacterium spp*., and an enrichment of conditionally pathogenic genera, such as *Aspergillus*.^25^ The aforementioned imbalance can aggravate hepatic fat accumulation and inflammation through multiple pathways, including disruption of lipid absorption, bile acid reabsorption, metabolic signaling, and intestinal barrier integrity.^25^ In the present study, a marked reduction in *B. uniformis* was identified in patients with MAFLD, particularly in those with moderate-to-severe hepatic steatosis and obesity. Transplantation of GM from MAFLD patients induced MAFLD-like phenotypes in mice, whereas transplantation from healthy controls or *B. uniformis* supplementation improved disease features and increased the abundance of beneficial genera, including *Firmicutes* and *Ligilactobacillus*.^25,26^ These findings indicate that GM dysregulation is linked to MAFLD progression and suggest that targeting the microbiota may provide a strategy to restore liver health.*B. uniformis*, a key commensal species in the human gut microbiome, has been shown to improve hepatic metabolism and immune function through modulation of SCFA production and other metabolites.^15,27^ Previous studies have demonstrated that *B. uniformis* regulates GM composition and reduces colonic inflammation in mice, effects associated with altered bile acid metabolism and regulation of key proteins involved in the NF-κB and MAPK signaling pathways.^28^ Reduced abundance of *B. uniformis* has also been reported in individuals with cardiovascular disease compared with healthy controls, further supporting its therapeutic potential.^29^ Despite these findings, the specific mechanisms by which *B. uniformis* contributes to MAFLD remain poorly understood.

Limited research has explored the relationship between gut dysbiosis and phenotypic variation in MAFLD across populations with different body mass indexes. A reduction in microbial diversity was observed in non-obese individuals with MAFLD. To identify biologically relevant features distinguishing these populations, differential abundance analysis revealed decreased levels of *Intestinibacter bartlettii* and *Turicibacter sanguinis* in the non-obese MAFLD group. LefSe analysis further identified *Romboutsia timonensis*, *Bilophila*, *Bilophila wadsworthia*, and *Mitsuokella multacida* as potential contributors to non-obese MAFLD, with *Bilophila*, *Bilophila wadsworthia*, and *Turicibacter sanguinis* identified as key discriminatory taxa between the two groups. *Bilophila*, a bile-tolerant microorganism associated with animal-fat-rich diets, has been linked to increased intestinal inflammation.^30^ Elevated *Bilophila* wadsworthia abundance under high-fat dietary conditions contributes to impaired intestinal barrier function, heightened inflammatory responses, disrupted bile acid metabolism, and hepatic steatosis.^30,31^ *Turicibacter* colonization has been shown to regulate genes involved in bile acid and lipid metabolism, influencing host lipid homeostasis.^32,33^ *Turicibacter sanguinis*, the most studied species within this genus, has been reported to modulate intestinal lipid gene expression and lower serum TG in mice.^34^ Further studies with larger sample sizes and investigations at cellular, molecular, and animal levels are necessary to determine whether these microbial differences contribute to phenotypic variation in MAFLD across different BMI categories.

Most existing studies have focused on broad alterations in GM composition and conventional metabolite profiles, such as SCFA and secondary bile acids. However, the specific regulatory mechanisms linking strain-level metabolites to host signaling pathways in MAFLD pathogenesis remain largely unexplored.^26,35^ In the present study, *B. uniformis* and its potential putative metabolite HDA were precisely identified as key factors associated with MAFLD symptoms. Functional investigations conducted in vivo and in vitro provided direct evidence supporting the causal role of both the bacterium and its metabolite in MAFLD progression.

The smooth endoplasmic reticulum (sER) is the central hub for de novo lipogenesis (DNL), making endoplasmic reticulum stress (ERS) a critical driver of hepatic steatosis.^36,37^ Under chronic ERS conditions, lipid accumulation is exacerbated through three key mechanisms: (1) upregulation of DNL and adipose tissue lipolysis, (2) suppression of mitochondrial fatty acid (FA) β-oxidation, and (3) impaired assembly and secretion of very-low-density lipoproteins (VLDL). The IRE1α-XBP1 pathway, a major arm of the unfolded protein response (UPR), becomes activated during ERS through a sequential process: (1) dissociation of IRE1α from its inhibitory chaperone GRP78, (2) oligomerization and autophosphorylation that triggers its RNase activity, and (3) unconventional splicing of XBP1 mRNA to generate the transcriptionally active spliced isoform (XBP1s).^36,37^ XBP1s then translocates to the nucleus, where it upregulates key lipogenic genes, including diacylglycerol O-acyltransferase 2 (DGAT2) and sterol regulatory element-binding protein 1c (SREBP1c), further promoting hepatic lipid deposition. Our experimental findings, supported by existing literature, demonstrate that Bacteroides uniformis and its putative metabolite HDA ameliorate MAFLD through a multi-pronged mechanism: (1) inhibition of intestinal fat absorption via suppression of luminal lipid emulsification and micelle formation, thereby reducing dietary lipid uptake; (2) suppression of eWAT lipolysis, leading to decreased free fatty acid (FFA) release into circulation and subsequent attenuation of hepatic FFA influx through the gut-liver axis; (3) direct modulation of the hepatic IRE1α-XBP1s pathway, resulting in downregulation of DGAT2 and microsomal triglyceride transfer protein (MTP) expression while alleviating ER stress; and (4) inhibition of ferroptosis by restoring glutathione peroxidase 4 (GPX4) activity, thereby improving hepatocyte lipid handling. Together, these coordinated mechanisms significantly reduce hepatic steatosis, highlighting Bacteroides uniformis and HDA as promising candidates for microbiota-targeted MAFLD therapy.

Disrupted hepatic iron balance is strongly linked to MAFLD progression, contributing to iron overload, elevated reactive oxygen species, and lipid peroxidation, ultimately triggering ferroptosis. Ferroptosis, a controlled cell death pathway, is closely involved in the progression of various liver disorders such as MAFLD, acute liver injury/failure (ALI/ALF), immune-mediated hepatitis, alcoholic liver disease, and hepatic fibrosis. Inhibiting ferroptosis has emerged as a promising strategy to slow liver disease progression.^38,39^ The transcription factor Nrf2 is essential for suppressing ferroptosis by promoting antioxidant defenses through genes such as HO-1 and GPX4, limiting MAFLD severity.^38,39^ Increasing evidence also suggests that interactions between ferroptosis and the GM contribute to MAFLD pathogenesis. Previous studies have shown that gut microbial metabolites, such as tauro-β-muricholic acid, promote ferroptosis through TFR-ACSL4 signaling, facilitating the development of toxin-associated MAFLD.^40^ Hepatic transcriptomic and proteomic analyses in *B. uniformis*-treated MAFLD mice identified ferroptosis as a key downregulated pathway. These findings suggest that *B. uniformis* may inhibit ferroptosis through activation of the Nrf2/SLC7A11/GPX4 pathway, thereby slowing MAFLD progression and providing new insight into potential therapeutic approaches for MAFLD management.

Endoplasmic reticulum (ER) stress is critical in metabolic disorders and inflammatory responses. The transcription factor XBP1 regulates ER stress responses, fatty acid metabolism, and lipid homeostasis. Overactivation of XBP1 triggers sustained inflammatory responses and amplifies OS, forming a positive feedback loop with ferroptosis. This mechanism has been indicated in tumorigenesis, neurodegeneration, and metabolic diseases, highlighting XBP1 as a potential therapeutic target for hepatic metabolic disorders and inflammation. Previous findings identified XBP1 as a key regulator of hepatic ferroptosis and lipid accumulation. Knockdown of XBP1 in HepG2 cells significantly reduced lipid deposition and OS under high-fat conditions, accompanied by decreased intracellular iron levels and mitigation of ferroptosis-related mitochondrial alterations observed by electron microscopy. These results support the involvement of the “HDA-XBP1-ferroptosis” signaling axis. Further analysis demonstrated that XBP1 knockdown reduced intracellular ALT and TG levels compared with the model group. Disruptions in lipid metabolism are closely linked to ferroptosis, and modulation of XBP1 may enhance cellular resistance to ferroptosis by improving lipid metabolic pathways.^41,42^ In addition, OS indicators, including MDA and Fe^2 +^, were significantly reduced following XBP1 knockdown, while glutathione (GSH) levels increased. These results suggest that XBP1 knockdown alleviates OS, strengthening cellular defenses against ferroptosis. OS is a key trigger of ferroptosis, while activation of the Nrf2 pathway effectively suppresses oxidative damage and protects cells.^43^ XBP1 knockdown may enhance antioxidant capacity through Nrf2 pathway activation, further increasing resistance to ferroptosis. Regulation of ferroptosis via the XBP1/Nrf2 axis advances understanding of cellular OS responses and identifies potential therapeutic targets. However, the precise molecular mechanisms by which HDA modulates XBP1 activity to regulate ferroptosis remain unclear. Future studies will focus on comprehensive mechanistic analyses to address these critical questions.

Hepatic fatty acid uptake and adipogenesis are elevated in MAFLD, and excessive free fatty acids contribute to hepatocellular apoptosis, inflammation, and insulin resistance.^44^ However, emerging evidence suggests that certain fatty acids and their derivatives exert protective effects during MAFLD progression. Population-based lipidomic analyses have reported reduced hepatic levels of omega-3 (ω-3) fatty acids in individuals with MAFLD.^45^ In animal models, both endogenous and exogenous ω-3 fatty acid supplementation alleviated hepatic steatosis and inflammation.^45^ Previously considered detrimental, trans-oleic acid (EA) has been shown to inhibit tumor growth and enhance the efficacy of PD-1 blockade therapy.^46^ Untargeted metabolomic analyses of portal plasma, feces, and liver tissues from MAFLD mice treated with *B. uniformis*, along with supernatant profiling of *B. uniformis* cultures, identified HDA as a key metabolite associated with the therapeutic effects of *B. uniformis*. HDA is a long-chain dicarboxylic acid with 16 carbons and two carboxyl groups, known to be transported via OATP1B and involved in hepatic and renal lipid metabolism.^47^ HDA has been shown to stimulate hepatic mitochondrial respiration, particularly during succinate oxidation, significantly increasing the rate of free mitochondrial respiration without concurrent ATP synthesis. This effect may support cellular defenses against OS and metabolic dysregulation.^48^ Although the molecular targets and signaling pathways of medium- and long-chain fatty acids such as HDA in the liver remain incompletely characterized, these fatty acids and their derivatives are widely recognized to activate G protein-coupled receptors (GPCRs), including GPR40 (FFAR1) and GPR120 (FFAR4), which regulate metabolic and inflammatory pathways in hepatocytes, pancreatic β-cells, and enteroendocrine cells.^49^ GPR40 is highly expressed in hepatic and pancreatic tissues and plays an important role in the pathogenesis of NAFLD and insulin resistance, positioning it as a key target in metabolic disease research. Binding analysis indicated a strong interaction between HDA and GPR40, suggesting that GPR40 may link HDA with the XBP1-ferroptosis pathway, providing a theoretical framework for targeted therapies in MAFLD.

Our study also has some shortcomings. Currently, there is no direct evidence demonstrating that HDA is the sole or primary driver of B. uniformis-mediated MAFLD improvement. Future studies employing HDA genetic knockout models will be required to definitively establish its specific contribution. Besides, MAFLD-FMT + B. uniformis Intervention is scientific Rationale, in Future studies We will specifically evaluate the efficacy-enhancing effects of B. uniformis in the MAFLD-FMT context. Future Direction is to quantify B. uniformis-mediated augmentation effects in FMT recipients and test HDA production and microbial network stability.

The present study demonstrated that *B. uniformis* and its potential putative metabolite HDA may contribute to MAFLD progression modulation, through inhibiting intestinal fat absorption, FFA from eWAT influx into liver via the gut-liver axis and regulation of the IRE1α-XBP1s axis. Future research should further evaluate the clinical potential of *B. uniformis* and HDA, particularly regarding their roles in improving liver function and regulating metabolic homeostasis. In addition, combining *B. uniformis* with other therapeutic strategies, such as dietary interventions and pharmacological treatments, may enhance its efficacy in MAFLD management and provide more comprehensive therapeutic options for patients.

## Supplementary Material

Supplementary Materials.docx

## Data Availability

This paper does not report the original code. Original data for creating all graphs in the paper are provided in Data S1. Human fecal macrogenomic data and mice fecal 16srRNA data have been deposited, and you can visit https://dataview.ncbi.nlm.nih.gov/object/PRJNA1193240. Human Metabolomics data have been deposited to the EMBL-EBI MetaboLights database (https://www.ebi.ac.uk/metabolights/editor/console) with the identifier MTBLS11947. Untargeted metabolomics data of portal vein serum, stool, and liver tissues from ABX mice and *B. uniformis* Supernatant have been deposited to the EMBL-EBI MetaboLights database (https://www.ebi.ac.uk/metabolights/editor/console) with the identifier MTBLS11923, MTBLS12013, MTBLS12265, MTBLS12266 respectively. Mice liver proteomics data has been uploaded IPX (https://www.iprox.cn/page/MSV022.html)with the identifier 0010522000. Data on mouse liver transcriptomics have been uploaded to https://dataview.ncbi.nlm.nih.gov/object/PRJNA1177604.
