# Supplementary material for: Bacteroides uniformis-generated hexadecanedioic acid ameliorates metabolic-associated fatty liver disease
Source: Gut Microbes. 2025 May 25;17(1):2508433. doi: 10.1080/19490976.2025.2508433 (PMC12118425; doi:10.1080/19490976.2025.2508433)
Supplement: Supplementary Materials.docx [file KGMI_A_2508433_SM4943.docx]

# Bacteroides uniformis-generated Hexadecanedioic Acid ameliorates Metabolic-associated Fatty Liver Disease

**Da-Ya Zhang^1,2^, Da Li^1,2^, Shi-Ju Chen^1,2^, Li-Jun Zhang^3^, Xu-Li Zhu^4^, Fa-Di Chen^5^, Chen Chen^1,2^, Qi Wang^1,2^, Yiping Du^6^, Jian-Xin Xiong^7^, Shi-Mei Huang^1,2^, Xiao-Dong Zhang^1,2^, Yan-Ting Lv^1,2^, Fan Zeng^1,2^, Run-Xiang Chen^1,2^, Xianfeng Huang^1,2^, Fengjiao Mao^1,2^, Shuo Zhou^1,2^, Qicen Yao^8^, Yuliang Huang^1,2^, Runyu Chen^1,2^, Ying Mo^1,2^, Yunqian Xie^2^, Yue-Hong Jiang^9^, Zhai Chen^10^, Cui-Yi Mo^11^, Jia-Jia Chen^11^, and Fei-Hu Bai^2,12^***

^1^The Second School of Clinical Medicine, Hainan Medical University, Haikou, China, 571199

^2^Department of Gastroenterology, The Second Affiliated Hospital of Hainan Medical University, Haikou, China, 570216

^3^Health Management Center, The Second Affiliated Hospital of Hainan Medical University, Haikou, China, 570216

^4^Otog Front Banner People's Hospital, Otog Front Banner, China, 016200

^5^Wuzhishan Center for Disease Control and Prevention, Wuzhishan, China, 572299

^6^Cardiovascular Surgery, The Second Affiliated Hospital of Hainan Medical University, Haikou, China, 570216

^7^Department of Gastroenterology, Hainan Second People's Hospital, Wuzhishan, China, 572299

^8^Department of Rheumatology and Immunology, The Second Affiliated Hospital of Hainan Medical University, Haikou, China, 570216

^9^Department of Gastroenterology, The Second People's Hospital of Ledong Li Autonomous County, Ledong Li Autonomous County, China, 572500

^10^Department of Gastroenterology, Dongfang People's Hospital, Dongfang, China, 572699

^11^Department of Gastroenterology, Qionghai People's Hospital, Qionghai, China, 571400

^12^The Gastroenterology Clinical Medical Center of Hainan Province, Haikou, China, 570216

*Correspondence: Feihu Bai, Chief Physician and Professor of the Department of Gastroenterology, The Second Affiliated Hospital of Hainan Medical University, Yehai Avenue, #368, Longhua District, Haikou, Hainan Province.

Tel: +86-18995181963

Email: 875025464@qq.com;

Fax: 0898-66809168

ORCID: 0000-0002-1560-6131

**Supplementary methods**

**Inclusion and exclusion criteria for MAFLD**

MAFLD was screened using abdominal ultrasound or CT according to the international expert consensus statemen^1^. MAFLD study participants must have (1) varying degrees of hepatic steatosis on abdominal ultrasound. (2) a. Overweight/obesity (BMI ≥ 23 kg/m2); b. Presence of type 2 diabetes mellitus (fasting glucose ≥ 7.0 mmol/L; and c. Metabolic dysfunction, with at least two of the following: a) High waist circumference; b) Hypertension; c) Hypertriglyceridemia; d) high-density lipoprotein cholesterol; e) prediabetes; f) insulin resistance; g) ultrasensitive C-reactive protein levels.

Exclusion criteria included: (1) specific diseases that may cause steatosis, such as viral hepatitis, alcoholic liver disease, drug-induced liver disease, and autoimmune liver disease. (2) other chronic diseases, including, but not limited to, chronic infections, significant intestinal disorders or symptoms (including, but not limited to, chronic constipation, diarrhoea, ulcerative colitis (UC), Crohn's disease (CD), intestinal obstruction, and intestinal malignancies). (3)other severe acute cardiovascular disease, renal disease, malignancies, etc., that may have interfered with data analysis in the month prior to the screening visit. (4) cirrhosis of the liver. (5) patients with dietary ‘abnormalities’ (e.g., vegan diets) in the past 12 months, or exposure to medications or interventions (e.g., drugs or interventions) that affect the composition of the intestinal microbiome in the past 3 months. (6) Patients who have been exposed to medications or interventions that affect the composition of the gut microbiome (e.g., antibiotics, immunosuppressants, chemotherapy, proton pump inhibitors, etc.) in the past 3 months. (7) Excessive consumption of alcohol (>1000 ml of beer or >200 ml) or a sudden major change in diet in the 1 week after sample collection. (8) Any condition unsuitable for the study, such as malaise or poor compliance. The healthy control group was created by matching without MAFLD by age, sex, ethnic, and BMI.

Ultrasound physician blinded to the study further classified it as (1) mild HS or moderate or severe HS. Ultrasound in patients with mild HS may show normal liver size and morphology, but with enhanced echoes in the anterior field, insignificant attenuation of echoes in the posterior field, and well-defined intrahepatic ductal structures. Moderate HS may show normal liver size and morphology, or mild or moderate enlargement, with enhanced anterior field echoes, attenuated posterior field echoes, and blurred intrahepatic ductal structures, but still recognizable. Severe HS shows that the liver is obviously enlarged, full in shape, with obvious enhancement of anterior field echo, obvious attenuation of posterior field echo, and even may present anechoic area, with unclear contour and difficult to recognize tubular structure. We defined MAFLD with BMI < 28 kg/m^2^ as non-obese MAFLD and MAFLD with BMI ≥ 28 kg/m^2^ as obese MAFLD. MAFLD individuals with normal weight (BMI <23 kg/m^2^), has been defined as normal weight^2^. MAFLD individuals (23 kg/m^2^ ≤BMI <28 kg/m^2^), has been defined as overweight. Non-obese MAFLD included MAFLD with normal weight or overweight body. Out of 120 MAFLD patients, 39 patients with moderate to severe HS (msMAFLD), and 81 patients with mild HS (miMAFLD) were included in overall and subgroup analysis. Out of 120 MAFLD patients, 46 MAFLD with BMI ≥ 28 kg/m^2^ as obMAFLD and 74 MAFLD with BMI <28 kg/m^2^ as nobMAFLD were included in overall and subgroup analysis.

***Anthropometric and demographic measurements***

The study physician administered a detailed written questionnaire^3^ to each participant to collect their characteristics. Anthropometric measurements, including weight, height, blood pressure, were performed by trained nurses using standard protocols and calibrated instruments. BMI was calculated as weight (kilograms) divided by the square of height (meters). We collected detailed scales of personal circumstances including age, sex, BMI, alcohol consumption, smoking, physical activity, mental stress, sleep conditions, diabetes, hypertension, medical history, etc. The dietary questionnaire was conducted by a full-time dietitian. A paper-based questionnaire with a combination of pictures, videos, voice conversations and video calls was used to ensure the reliability of the dietary intake information, taking into account the volunteers' literacy level, language communication and other interfering factors. Food types were categorized into 5 main groups (main food, side dishes, fruits, beverages and other local specialties). The dietary questionnaire was designed for the Chinese population and included traditional Chinese foods such as staple foods, side dishes (a variety of meats and vegetables), fruits, and beverages (tea and coffee), as well as foods native. Intake of these foods was recorded as yes or no in the past 1 month and on the last 1 day. The dietary questionnaire used in this study was designed according to the 2016 Dietary Guidelines for Chinese Residents. The revised questionnaire also underwent two rounds of pilot testing and validation before it was officially used in this study. If some items in the questionnaire were confusing, or if respondents suggested improvements to items in the questionnaire, the questionnaire was further refined to ensure clarity, information and variability of answers.

***Sample collections and clinical laboratory measurements***

Fasting venous blood samples were collected from the veins of the subjects after 12 hours of fasting. Each volunteer collected 2 tubes of venous blood samples in the morning in health centers, one red riser tube for biochemical indexes and one purple tube for metabolomics and blood routine. The purple tube of blood samples needs to be centrifuged at 3000 rpm for 10 minutes in a high speed refrigerated centrifuge to obtain serum for testing. Blood routine include blood cells, neutrophils (Neu), lymphocytes (Lym), monocytes (Mono), Red blood cell distribution width (RDW), platelets (PLT) and other routine blood tests. Biochemical variables include serum K+, Na+, Cl-, Ca+, alanine aminotransferase (ALT), aspartate aminotransferase (AST), glutamyl transpeptidase (GGT), alkaline phosphatase (ALP), total bilirubin (TBIL), direct bilirubin (DBIL), indirect bilirubin (IBIL), creatinine (Cr), uric acid (URIC), Blood urea nitrogen (BUN), high-density lipoprotein cholesterol (HDL-C), triglycerides (TG), cholesterol (CHOL), low-density lipoprotein (LDL-C), fasting plasma glucose(FPG). Blood routine was detected by LH780 blood cell analyzer. Other indexes were tested by Cobas 6000 auto-analyzer. The testing of the above indicators is done by the Laboratory Department of hospital.

The participants received oral and written instructions regarding the stool collection procedure under non-fasting conditions. Stool collection kits for self-collection were provided to the participants, who were required to deliver at least 30 g of fresh stool at ambient temperature to the laboratory within 2 h of defecation. On arrival, samples were subsequently aliquoted and placed at −80°C.

All stool samples and Venous blood plasma from HN and NM were shipped overnight by special post to SHANGHAI BIOTREE BIOTECH CO., LTD, where they were stored in −80°C freezers until further testing.

***Fecal Suspension Preparation for FMT:***

Donor stool samples were processed within 30 min of collection in pre-reduced, anaerobic PBS. Fecal material was homogenized with ice-cold anaerobic PBS (1:10 ratio, 200 mg feces/2 mL) under anaerobic conditions. The mixture was vortexed until homogeneous, then sequentially filtered through sterile mesh filters (200-, 400-, and 800-μm pore sizes) to remove particulate matter. The filtrate was vortexed (5 min), centrifuged (600×g, 5 min), and the supernatant collected as FMT inoculum.

***Biochemical index detection in mice***

Serum biochemistry analysis Levels of serum triglycerides (TG), total cholesterol (TC, TCHO), high density lipoprotein cholesterol (HDL-C), low density lipoprotein cholesterol (LDL-C), alanine transaminase (ALT), aspartate transaminase (AST), alkaline phosphatase (ALP), creatinine (Cr), uric acid (URIC), Blood urea nitrogen (BUN), were measured by the automatic biochemical analyzer (XR220 Plus) as instructed by the manufacturer. Iron metabolism (iron, ferritin) and oxidative stress（MDA, GSH）were determined according to the manufacturer's instructions (Mlbio, Shanghai, China). All experiments were performed in triplicate.

***Enzyme-linked immunosorbent assay (ELISA)***

LPS and inflammatory factors (TNF-α, IL-1β) were determined using commercial ELISA kits according to the manufacturer's instructions (Mlbio, Shanghai, China). All experiments were performed in triplicate.

***ROS Testing***

***Tissue ROS steps:***

1. fresh tissue is immediately placed in pre-cooled PBS and cleaned of blood and other contaminants.
2. remove block dead components, fibers, fats and blood vessels from the tissue block.
3. use ophthalmic scissors to cut the tissue block into small pieces of about 1mm3, put it in pre-cooled tissue culture solution or PBS and rinse it to wash away the clipped cell debris.
4. Add appropriate amount of enzyme digestive solution and digest for 20-30min at 37℃ in a constant temperature water bath, during which the cells were intermittently shaken or blown.
5. Terminate the digestion with PBS, remove the tissue clumps by cell sieve filtration, collect the filtered cells, centrifuge at 500g for 10min, remove the supernatant and leave the precipitate, and wash with PBS for 1～2 times.
6. Resuspend the cell precipitate with diluted DCFH-DA at a cell density of 1×10^6^mL.
7. Incubate the cells at 37°C for 30 min, mixing upside down every 3-5 min so that the probe is in full contact with the cells. Collect the single cell suspension after incubation (probe labeling), 1000g, centrifuge for 5-10 min, remove the supernatant to collect the cell precipitate, and wash with PBS 1-2 times to fully remove the DCFH-DA that has not entered the cells.
8. Fluorescence zymography detection, wavelength setting: the best excitation wavelength 488nm, the best emission wavelength 525nm.

***Cellular ROS steps:***

1. DCFH-DA probe is added to serum-free medium: 1:1000 dilution of DCFH-DA with serum-free culture medium (final concentration of 10µM).
2. Collect the cell precipitate and add an appropriate volume of diluted DCFH-DA to cover the cells, usually not less than 1mL of diluted DCFH-DA is added to one well of a 6-well plate.
3. Incubate the cells at 37℃ for 30min (mix upside down every 3~5 minutes to make full contact between the probe and the cells)
4. Aspirate off the culture solution, and clean it by utilizing serum-free culture solution or 0.01MPBS.
5. Sample testing on the machine

***Histopathology***

**HE testing of liver, epididymal white adipose tissue (eWAT), colon and ileum tissues**

Referring to the instructions for HE staining, the sections were dewaxed through paraffin sections to water - hematoxylin staining - eosin staining - dehydration sealing. Final microscopic examination, image acquisition and analysis (nucleus blue, cytoplasm red).

**Oil red O staining of liver**

Frozen sections were rewarmed and dried, fixed in 4% paraformaldehyde for 15 min, washed and dried, poured into the oil red working solution and dipped for 8-10 min (avoiding light), washed; 75% alcohol was slightly differentiated. Hematoxylin re-staining about 3-5 min, tap water wash, hydrochloric acid alcohol rapid differentiation, ammonia aqueous solution to return to the blue, water wash. Absorb water from the periphery with filter paper, glycerol gelatin seal (lipid droplets are orange to bright red, nuclei blue).

**Sirius Red staining**

Deparaffinized tissue section, rehydration, Sirius Red staining, tissue sectioning and dehydration transparency; sealing: neutral gum sealing, microscopic observation of the liver tissue of each group of mice and photographing.

**Scoring of liver tissues**

Liver tissues were assessed for steatosis by H&E staining, and the degree of steatosis was assessed using the Non-Alcoholic Steatohepatitic Liver Disease Activity Score (NAS), which represents the sum of three scores, i.e., severity of steatosis (0-3), lobular inflammation (0-2) and hepatocyte swelling (0-3). Fibrosis was assessed by Sirius red staining and liver fibrosis was scored on a 5-point scale (0-4).

***Cell Transmission electron microscopy (TEM)***

1. Take the material and fix it:
2. Cultured cells discard the medium, add trypsin digestion. After trypsin digestion, add medium to terminate the digestion, and gently blow the cells with a pipette until they float. Suction into the centrifuge tube, low-speed centrifugation (1500-3000rpm), about 3-5min, discard the supernatant, add the electron microscope fixative (room temperature), room temperature and avoid light fixation for 30min, and then transferred to 4 °storage.
3. Pre-embedding: centrifuge the sample and discard the supernatant, add 3% low melting point agar. Centrifuge at low speed, wait until the agar solidifies, then cut off the mass in 2.5% glutaraldehyde;
4. Post-fixation: the sample block 0.1M phosphoric acid rinsing solution rinsed 3 times, each time 15min, after the sample into the 1% osmium acid 4 ℃ refrigerator fixed 4h or overnight;
5. Sample dehydration: the samples were sequentially dehydrated into 30%-50%-70%-80%-95%-100%-100% alcohol in a gradient shock dehydration, 40min each time, 100% epichlorohydrin 3 times, 30min each time;
6. Infiltration and embedding: propylene oxide + embedding solution (2:1), room temperature shock infiltration for 4h, propylene oxide + embedding solution (1:2) room temperature shock overnight, pure embedding solution shock room temperature overnight, replace the pure embedding solution, room temperature shock infiltration for 3-4h. Then pick out the sample with a toothpick, and embed it in the embedding plate;
7. polymerization: the embedding plate is placed in the oven at 60 ℃ for 48h, until the resin is completely polymerized, remove the embedded block standby;
8. Semi-thin positioning and ultra-thin sectioning: after rough repair block, the resin block in the ultra-thin sectioning machine semi-thin sectioning positioning, according to the positioning of the repair block, the required position for ultra-thin sectioning, section thickness 70nm, copper mesh fishing slice;
9. Staining: 3% uranyl acetate saturated alcohol solution staining 8min; 70% alcohol cleaning 3 times, ultrapure water cleaning 3 times; 2.7% lead citrate solution staining 8min; ultrapure water cleaning 3 times, filter paper slightly absorbent;
10. Observation and photo-taking: observation under transmission electron microscope, collecting images for analysis.

***Tissue transmission electron microscopy:***

1. Sampling and fixation:
2. Animals were anesthetized/executed and quickly opened where the material needed to be taken. Rapidly cut a piece of tissue, saline or PBS quickly rinse and dip into glutaraldehyde on the cutting plate for further cutting; cut into 1mm³ small pieces, stick into 1.5ml EP tube with a water-soaked toothpick, fill up with 2.5% glutaraldehyde, and store at 4℃ away from light.
3. Pre-embedding: centrifuge the sample and discard the supernatant, add 3% low melting point agar suspension. Centrifuge at low speed, wait until the agar solidifies and then cut off the clumps in 2.5% glutaraldehyde;
4. Post-fixation: the sample block 0.1M phosphoric acid rinsing solution rinsed 3 times, each time 15min, after the sample into the 1% osmium acid 4 ℃ refrigerator fixed 4h or overnight;
5. Sample dehydration: the samples were sequentially dehydrated into 30%-50%-70%-80%-95%-100%-100% alcohol in a gradient shock dehydration, 40min each time, 100% epichlorohydrin 3 times, 30min each time;
6. Infiltration and embedding: propylene oxide + embedding solution (2:1), room temperature shock infiltration for 4h, propylene oxide + embedding solution (1:2) room temperature shock overnight, pure embedding solution shock room temperature overnight, replace the pure embedding solution, room temperature shock infiltration for 3-4h. Then pick out the sample with a toothpick, and embed it in the embedding plate;
7. polymerization: the embedding plate is placed in the oven at 60 ℃ for 48h, until the resin is completely polymerized, remove the embedded block standby;
8. Semi-thin positioning and ultra-thin sectioning: after rough repair block, the resin block in the ultra-thin sectioning machine semi-thin sectioning positioning, according to the positioning of the repair block, the required position for ultra-thin sectioning, section thickness 70nm, copper mesh fishing slice;
9. Staining: 3% uranyl acetate saturated alcohol solution staining 8min; 70% alcohol cleaning 3 times, ultrapure water cleaning 3 times; 2.7% lead citrate solution staining 8min; ultrapure water cleaning 3 times, filter paper slightly absorbent;
10. Observation and photo-taking: observation under transmission electron microscope, collecting images for analysis.

***Samples DNA extraction, 16S rRNA gene sequencing, and microbiome analysis***

Mice feces were collected and stored at -80℃, and 16SrRNA sequencing was commissioned to Shanghai Baiyun Biotechnology Co. Total genomic DNA was extracted from the samples using the CTAB method, and the DNA was diluted to 1 ng/μL using sterile water according to the concentration.The highly variable regions V3 and V4 of 16SrRNA were selected for amplicon generation and taxonomic analysis. DNA libraries were constructed using the TruSeqRDNA PCR-free Sample Preparation Kit, and the libraries were sequenced on the Illumina NovaSeq platform. Double-ended reads were merged using FLASH (V1.2.7, http://ccb.jhu.edu/software/FLASH/). Raw tags were quality filtered according to the QIIME (V1.9.1, http:/lqiime.org/scripts/split_libraries_fastq.html) quality control process. Sequence analysis was performed by Uparse software (Uparsev 7.0.1001, http://drive5.com/uparse/). In addition, dilution curves, relative abundance of species, PCoA and LDA effect size (LEfSE) analyses were performed in R software (version 2.15.3).

***Liver transcriptomic analysis in mice***

RNA extraction consisted of grinding 50-150 mg of mouse liver tissue, homogenization in MagZolTM reagent (Axygen, USA), addition of chloroform, and centrifugation to collect RNA. quality control consisted of agarose gel electrophoresis, Nanodrop spectrophotometric assay, and Qubit fluorescence assay. Library preparation was simplified by kits combining double-stranded synthesis, end repair, and dA-tailing. mRNA was enriched with VAHTS mRNA capture beads (Vazyme, China) and then fragmented for double-stranded cDNA synthesis. The cDNA was then purified and ligated into the junction. The libraries were quality checked using Agilent Bioanalyzer and Qubit. Sequencing was performed on an Illumina Novaseq6000 platform using PE150 (paired-end 150) mode. Quality control of the sequencing data (raw data) was performed using fastp (v2.0) to remove adapters, trim low-quality reads (score <20), and discard reads with more than 10% N content. Clean reads were aligned to the reference genome using HISAT2 (v2.1.0) and alignment quality was assessed using RSeQC (v3.0.1). Expression was quantified using StringTie and significantly different genes were identified using DESeq2 (with duplicates) or edgeR (without) based on the criteria of |log2 fold change ≥ 1 and padj < 0.05. Data were visualized using ggplot2, clustering, and heatmaps. GO and KEGG enrichment analysis was performed using Fisher's exact test, and core genes in the GO/KEGG annotated gene set were identified using GSEA (Gene Set Enrichment Analysis). Protein interactions were assessed using the STRING database. Metabolic pathways were visualized using Path3.0 to fully display the KEGG pathway. SNP/Indel analysis was performed using STAR for comparison and GATK for variant calling. The above experiments and sequencing procedures were performed by Shanghai Source Gene Biopharmaceutical Technology Co. Ltd (Shanghai, China), and academic protocols were established as required.

***Total RNA extraction and real-time quantitative polymerase chain reaction (qRT-PCR)***

Total RNA from liver tissues and cells was extracted and evaluated as the instruction manual of RNAiso Plus kit. The cDNA was synthesized by reverse transcription of the total RNA from liver tissue with the PrimeScriptTMRT kit with gDNA Eraser (Perfect Real Time). cDNA was stored in a refrigerator at -20℃. TB GreenPremix Ex Taq (Tli RNaseH Plus) kit was used for PCR amplification reaction. The expression levels of target mRNAs were detected using an ABIStepOnePlus real-time fluorescence quantitative PCR instrument. 18s were used as internal reference genes for mRNA level normalization. The validated genes were XBP1, NRF2, Hrd1, SLC7A11, GPX4, CD36, MTP, PPARγ, FABP4, DGAT2 and the primer sequences are shown in the Table.

**Table.** The primers used in qRT-PCR analysis in human

| Genes | Forward primer (5’--3’) | Revers primer (5’--3’) |
| --- | --- | --- |
| *Actin* | TCCTCCTGAGCGCAAGTACTCC | CATACTCCTGCTTGCTGATCCAC |
| *XBP1* | ACCAGGAGTTAAGACAGCGC | TTCATTCCCCTTGGCTTCCG |
| *Hrd1* | ACCAGCATCCCTAGCTCAGA | GAGCTGGAGGCCTTTCCATT |
| *NRF2* | GAGCAAGTTTGGGAGGAGCT | GGCTTCTGGACTTGGAACCA |
| *SLC7A11* | CTGAGCGGCTACTGGGAAAT | CGTTCATGGAGCCAAAGCAG |
| *GPX4* | CAGTGAGGCAAGACCGAAGT | CCGAACTGGTTACACGGGAA |

**Table. The primers used in qRT-PCR analysis in mice**

| Genes | Forward primer (5’--3’) | Revers primer (5’--3’) |
| --- | --- | --- |
| *Actin* | TCCTCCTGAGCGCAAGTACTCC | CATACTCCTGCTTGCTGATCCAC |
| *CD36* | GGAGGCATTCTCATGCCAGT | CTGCTGTTCTTTGCCACGTC |
| *MTP* | GGCACTTTGCAGTGTCTGTG | GTCACCAATGATGGCTCCCA |
| *PPARγ* | GGGGATGTCTCACAATGCCA | GATGGCCACCTCTTTGCTCT |
| *FABP4* | TTTGGTCACCATCCGGTCAG | ACACATTCCACCAGCTTGTCA |
| *IRE1ɑ* | CCCAGCACAGACCTCAAGTT | TGTTTGGGCAGGTTGTTAGGA |
| *XBP1* | CTGAGTCCGCAGCAGGTG | AGGTCCCCACTGACAGAGAA |
| *DGAT2* | GGCTACGTTGGCTGGTAACT | CTTCAGGGTGACTGCGTTCT |
| *Hrd1* | CCACCAGTACAGCCGTTTCT | GGCAGCATCCAGTAGTGTGT |
| *Nrf2* | CAGAGTGATGGTTGCCCACT | CACACACTTTCTGCGTGCTC |
| *SLC7A11* | ATGGTCAGAAAGCCAGTTGTG | CAGGGCGTATTACGAGCAGT |
| *GPX4* | CCAAAGTCCTAGGAAACGCCC | CCGGGTTGAAAGGTTCAGGA |

***Liver Proteomics analysis in mice***

(1) Samplepreparation

1) Protein extraction-Take 1/4 of the sample, add 200 𝜇 reagent 0 and 1 large and 2 small steel beads, grind at low temperature for 240 s at 70 HZ, sonicate for 20 min at 12,000 rpm in an ice-water bath, centrifuge at 4°C for 10 min, and transfer the supernatant to a new EP tube.

2) BCA quantification (BCAassay)

Aspirate BCA working solution to 96-well plate, 200 per well, 7 standard points, 1 blank. Add 20 samples (diluted accordingly) or standard proteins (BSA), shake at 37℃ for 30min, and detect the absorbance at 562nm. The standard curve was fitted according to the standard protein and the protein concentration of the corresponding samples was calculated.

3）Reduction&alkylation&Proteindigestion

Take 30 proteins, add 20 reagent A, incubate for 5 min at 1000 rpm at 95 ℃, cool the sample to room temperature, add 15 reagent B, mix well, and perform enzymatic digestion at 1000 rpm at 37 ℃ for 2 h. After the enzymatic digestion, add 55 reagent C, mix well, and terminate the enzymatic reaction. Add all the samples to the desalting column, centrifuge at 700g for 1min, repeat; 100 reagent D was added to the desalting column, centrifuge at 700g for 1min, repeat; 100 reagent E was added to the desalting column, centrifuge at 700g for 1min, repeat; put the desalting column into a new centrifugal tube, add 100 reagent F to the desalting column, centrifuge at 700g for 1min, repeat, the final sample volume was 200; vacuum freezing was used for the final sample volume of 200; the final sample volume was 200; the final sample volume was 200; the final sample volume was 200; the final sample volume was 200. The final sample volume was 200; vacuum freeze centrifuge concentrator to concentrate the sample, the test sample re-dissolved and added to the iRT on the machine.

(2) nanoLCMS/MSanalysis

For each sample, 200 ng of total peptides were separated by nanoUPLC nanoElute2 and then combined with a mass spectrometer (timsTOFPro2) equipped with a nanoliter ion source for data acquisition. Chromatographic separation was performed on a 75 μmID×15 cm reversed-phase column (PePSepC18, 1.9, 75×15 cm, Bruker, Germany). The mobile phase was acetonitrile aqueous formic acid system, in which mobile phase A was 0.1% formic acid aqueous solution and phase B was 0.1% formic acid acetonitrile solution. After the column was equilibrated with 100% of phase A, the sample was directly injected into the column by an autosampler, and then separated by a gradient of the column with a gradient duration of 45 min.

(3) Protein characterization

direct DIATM data retrieval (Spectronaut database search) The raw mass spectrometry files were imported into the Spectronaut (version 18.2.230802.50606; BiognosysAG) software for database search, and after the search was completed, the qualitative analysis was performed.

(4) Bioinformatics analysis

1) GO and KEGG enrichment analysis

The proteins were annotated according to GO and KEGG databases for their functions. The conditions for this differential protein screening were Foldchange ≥1.2 or ≤0.83 and P-value or P-value-chitest <0.05.

2) Construction of PPI network

DEPs with opposite expression trends between the two comparison groups were uploaded to the String database for PPI network analysis. The above experiments and sequencing procedures were performed by Shanghai Baxter and academic protocols were established according to the requirements.

***Western blotting***

The tissues and cells were mixed with the lysate solution, and the protein solution was collected by centrifugation at 12,000 rpm and 4°C for 10 min, and the total protein content was determined by BCA method. The proteins were denatured by keeping them in boiling water for 15 min, and the protein samples were separated by 10% SDS-PAGE (sodium dodecyl sulfate-polyacrylamide gel electrophoresis) gel and transferred to polyvinylidene fluoride (PVDF, 0.45 μm) membranes. The membranes were closed in skimmed milk for 30 min, and then incubated with primary antibodies (XBP1, NRF2, Hrd1, SLC7A11, GPX4, CD36, MTP, PPARγ, FABP4, DGAT2) overnight at 4ºC in a shaker. Subsequently the secondary antibody was diluted at 1:5000 and added to the incubation bath placed on a shaker at room temperature for 30 min. eventually, the cleaned membrane reacted sufficiently with the ECL solution to produce protein bands. The optical density values of the protein immunoblot bands were evaluated by ImageJ.

***Quantification of B. uniformis in fecal samples and colon tissue***

Real-time PCR (qPCR) was used to quantitatively analyze the abundance of *B. uniformis* in fecal samples from both humans and mice. The detailed methods for bacterial DNA extraction and qPCR analysis were described elsewhere. Fecal DNA was extracted by a TIAAmp Atool DNA Kit (TIANGEN, DP328) according to the manufacturer’s manual. After detecting the concentration, 450 ng of DNA was added to the octopus tube, and 25 µl of 2×Pro Taq master mix was added, with 1ul of forward primer (B. uniformis: F-AGTAGAGGCA GGCGGAAT) and backward primer (B. uniformis: R-CGAGCATCAGCGTCAGTT), respectively, and ddH2O was added to supplement the mixture to 50 µl. After reverse transcription was completed, 6×lodding buffer was added and mixed well for later use. The samples were added to 2% agarose gel under 120 V constant pressure and displayed in the gel imager after electrophoresis.

**Cell transfection**

1. SiXBP1 was synthesized in Jiangsu Saisofei Biotechnology Co., Ltd. and the transfection method was as follows:
2. The day before transfection, count the cells and add 1ml into 6-well plate, so that the cell density of the plate is not less than 2*10^5^ cells.
3. For each well, dilute 10ul of 100nM siRNA with 250ul of serum-free medium and incubate for 5min at room temperature.
4. For each well, dilute 5ul of Lipo3000 with 250ul of serum-free medium and incubate for 5min at room temperature.
5. Mix the liquids from 2 and 3 and incubate for 20min at room temperature.
6. Replace the pre-sorted adherent cells with serum-free medium, add the above mixture, incubate at 37℃, 5% CO2 for 6 hours, then replace with growth medium with blood, continue to incubate at 37℃, 5% CO2 for 48 hours. Cell samples were collected, washed twice with PBS, and the liquid was discarded for subsequent RNA extraction.

**References**

1. Eslam M, Newsome PN, Sarin SK, Anstee QM, Targher G, Romero-Gomez M, Zelber-Sagi S, Wai-Sun Wong V, Dufour JF, Schattenberg JM, Kawaguchi-Suzuki M, Kawaguchi T, Shiha G, Kawaguchi M, Campana LG, Hamaguchi M, Handa T, Lee CH, Yen CJ, Le M, et al. A new definition for metabolic dysfunction-associated fatty liver disease: An international expert consensus statement. J. Hepatol. 74, 202–209 (2021).
2. Iwaki M, Kessoku T, Ozaki A, Kasai Y, Kobayashi T, Nogami A, Honda Y, Ogawa Y, Imajo K, Yoneda M, Maeda A, Tanaka Y, Nakajima S, Ohno H, Usuda H, Kawanaka M, Kawaguchi T, Torimura T, Kage M, Hyogo H, Takahashi H, Eguchi Y, Aishima S, Wada K, Kobayashi N, Sumida Y, Saito S, Nakajima A. Gut microbiota composition associated with hepatic fibrosis in non-obese patients with non-alcoholic fatty liver disease. J Gastroenterol Hepatol. 2021 Aug;36(8):2275-2284.
3. Zuo T, Sun Y, Wan Y, et al. Human-Gut-DNA Virome Variations across Geography, Ethnicity, and Urbanization. Cell Host Microbe. 2020. 28(5): 741-751.e4.

**Supplementary Figures**


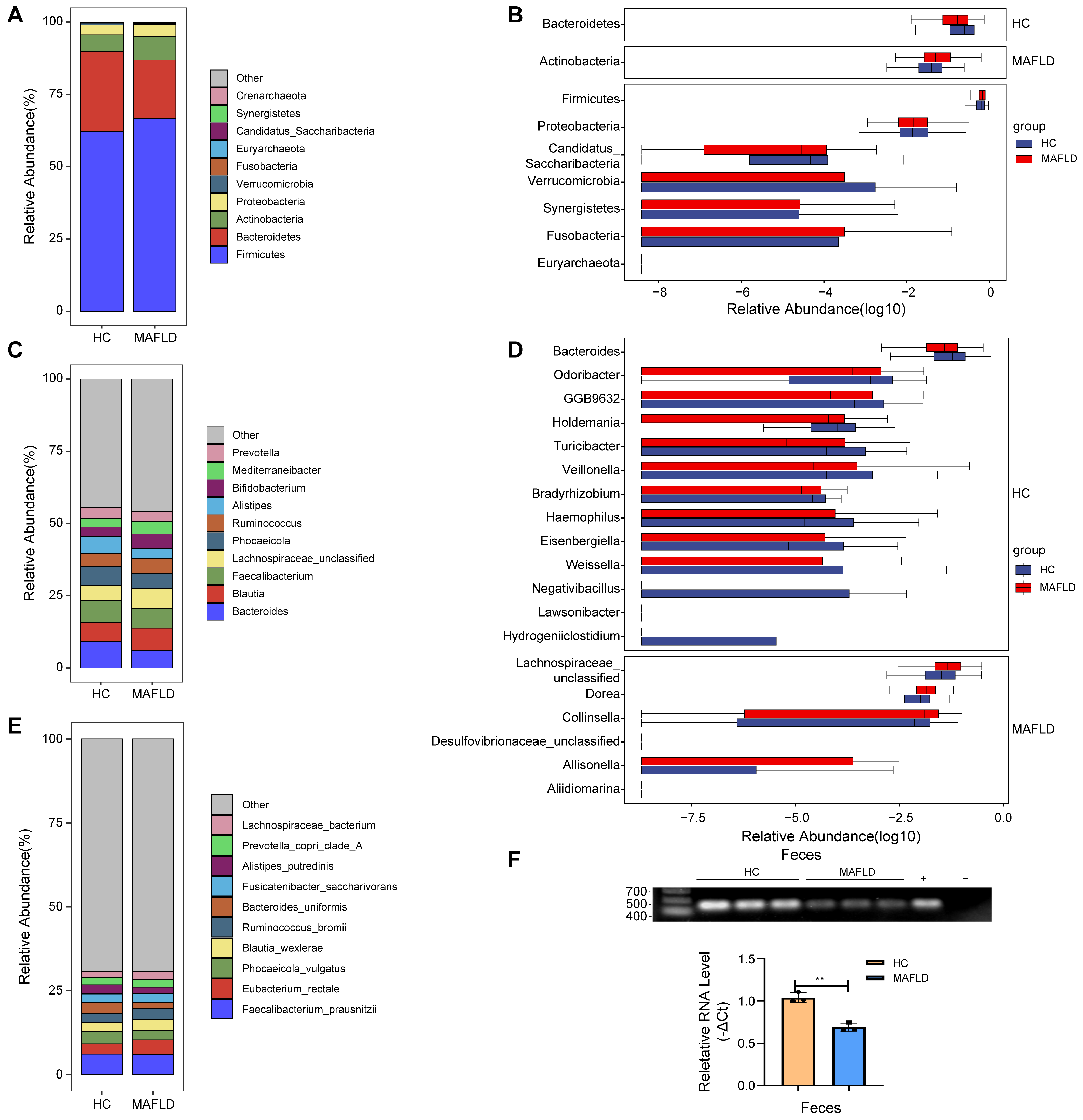


**Figure S1. Composition and abundance of phylum, genus, and species in MAFLD patients vs HC.** (A) Composition of dominant microbial communities at phylum levels. (B) Relative abundance of dominant microbial communities at the phylum level. (C) Composition of dominant microbial communities at genus levels. (D) Relative abundance of dominant microbial communities at the genus level. (E) Composition of dominant microbial communities at species levels. (F) Relative abundance of bacteroides_uniformis(n=3 for each group). (A-E)The analysis included 120 healthy controls (HC) and 120 metabolic-associated Fatty Liver Disease (MAFLD) patients, matched for age, sex, etc.


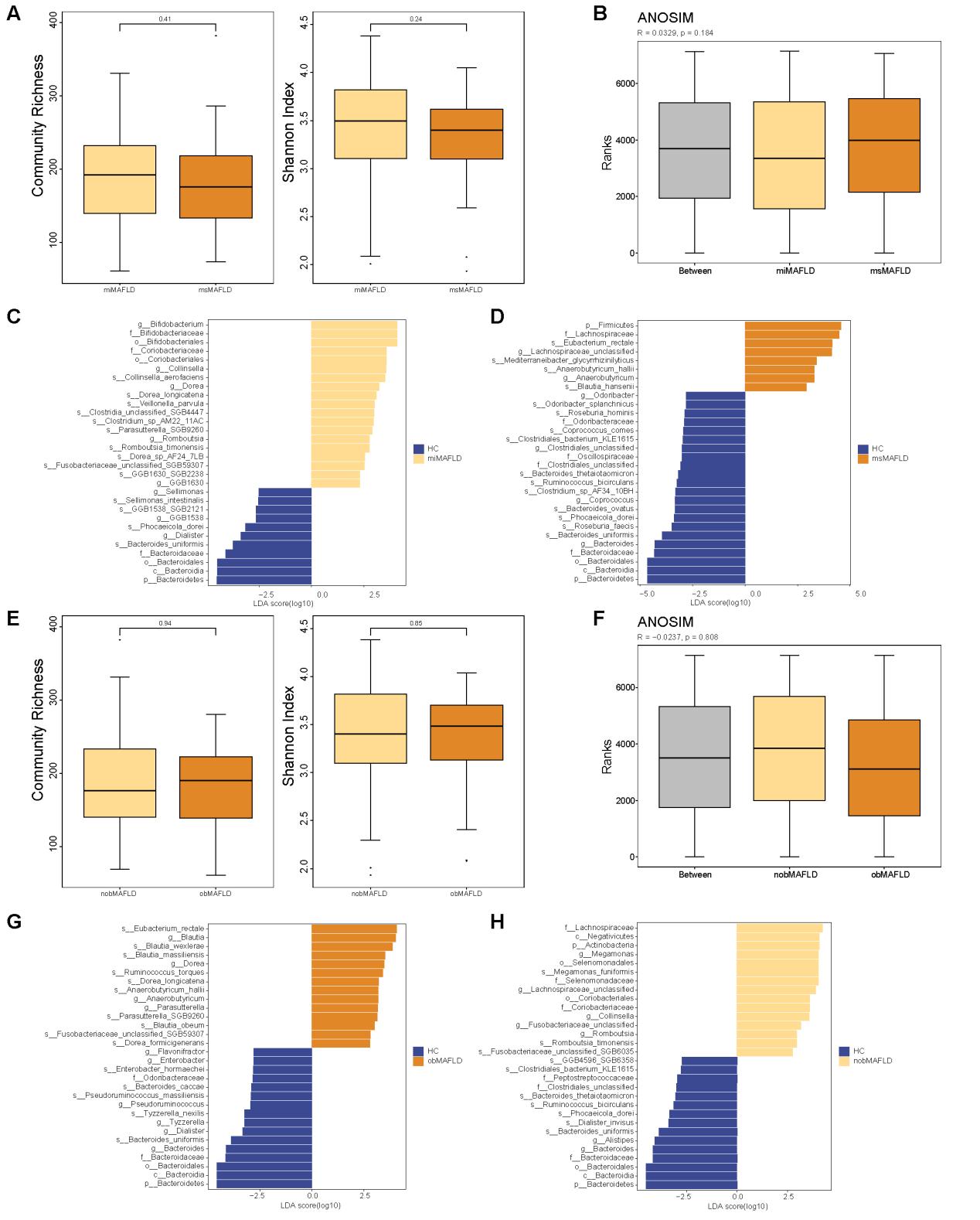


**Figure S2. Relationship between microbiota and HS, BMI.** (A) Alpha diversity indices (Community Richness and Shannon) of the intestinal bacterial communities of miMAFLD(n=81) vs msMAFLD group(n=39). (B) Beta differences determined by ANOSIM base on metagenomic sequencing in miMAFLD(n=81) vs msMAFLD group(n=39). (C) Differential analysis of species by [LEfSe](https://zhida.zhihu.com/search?content_id=136096456&content_type=Article&match_order=1&q=LEfSe&zhida_source=entity) in miMAFLD(n=81) vs HC group(n=120). (D) Differential analysis of species by [LEfSe](https://zhida.zhihu.com/search?content_id=136096456&content_type=Article&match_order=1&q=LEfSe&zhida_source=entity) in msMAFLD(n=39) vs HC group(n=120) . (E) Alpha diversity indices (Community Richness and Shannon) of the intestinal bacterial communities of nobMAFLD(n=74) vs obMAFLD group(n=46). (F) Beta differences determined by ANOSIM base on metagenomic sequencing in nobMAFLD(n=74) vs obMAFLD group(n=46). (G) Differential analysis of species by [LEfSe](https://zhida.zhihu.com/search?content_id=136096456&content_type=Article&match_order=1&q=LEfSe&zhida_source=entity) in nobMAFLD(n=74) vs HC group(n=120). (H) Differential analysis of species by [LEfSe](https://zhida.zhihu.com/search?content_id=136096456&content_type=Article&match_order=1&q=LEfSe&zhida_source=entity) in obMAFLD(n=46) vs HC group(n=120).


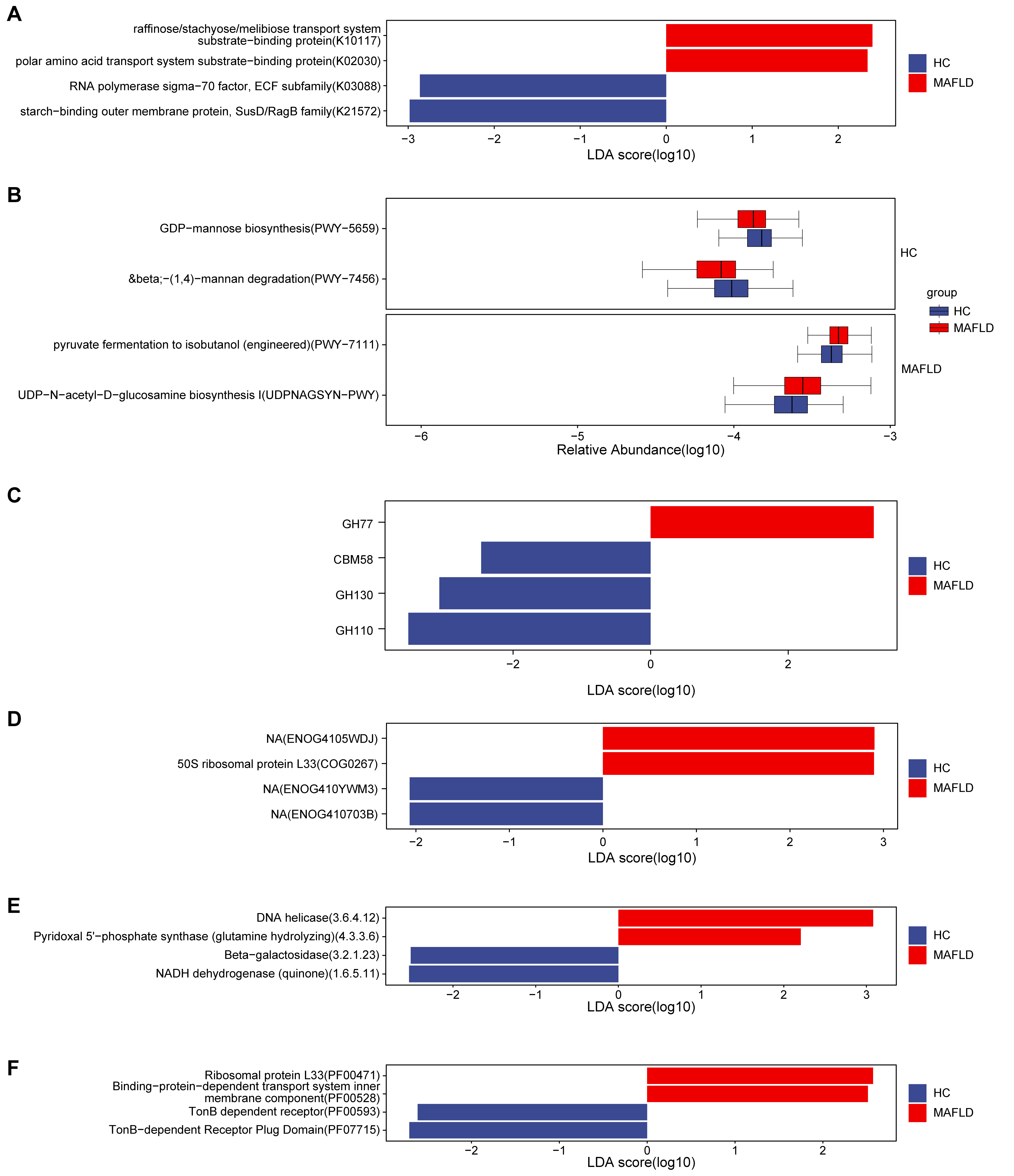


**Figure S3. Prediction of differential microbial community function.** (A) KEGG pathway. (B) MetaCyc database. (C) CAZy database. (D) EggNOG database. (E) EC database. (F) Pfam database. The analysis included 120 HC and 120 MAFLD patients, matched for age, sex, etc.


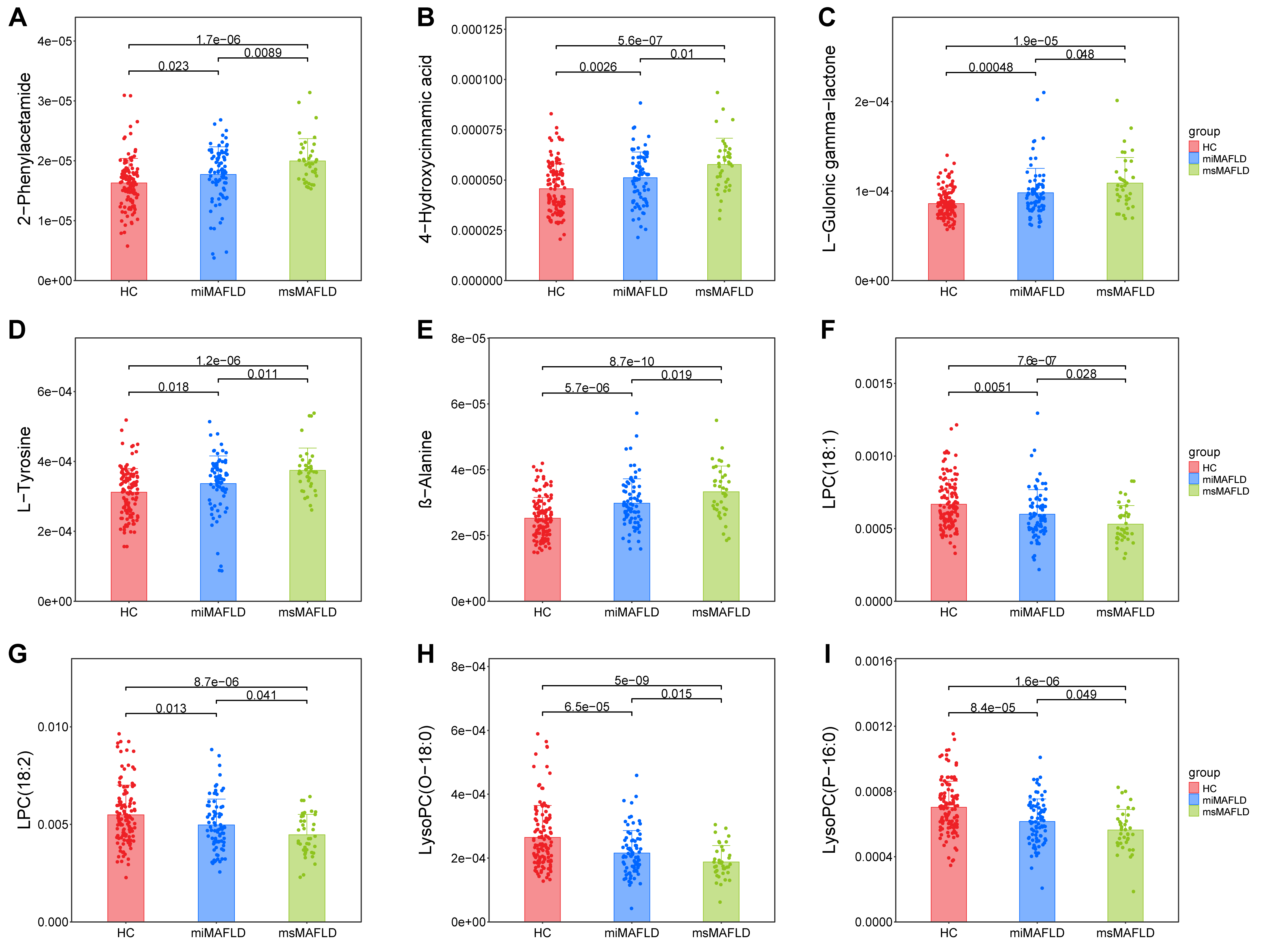


**Figure S4. Plasma differential metabolites associated with hepatic steatosis.**The analysis included 120 HC and 120 MAFLD patients, matched for age, sex, etc. Out of 120 MAFLD patients, 39 patients with moderate to severe HS (msMAFLD), and 81 patients with mild HS (miMAFLD) were included in overall and subgroup analysis.


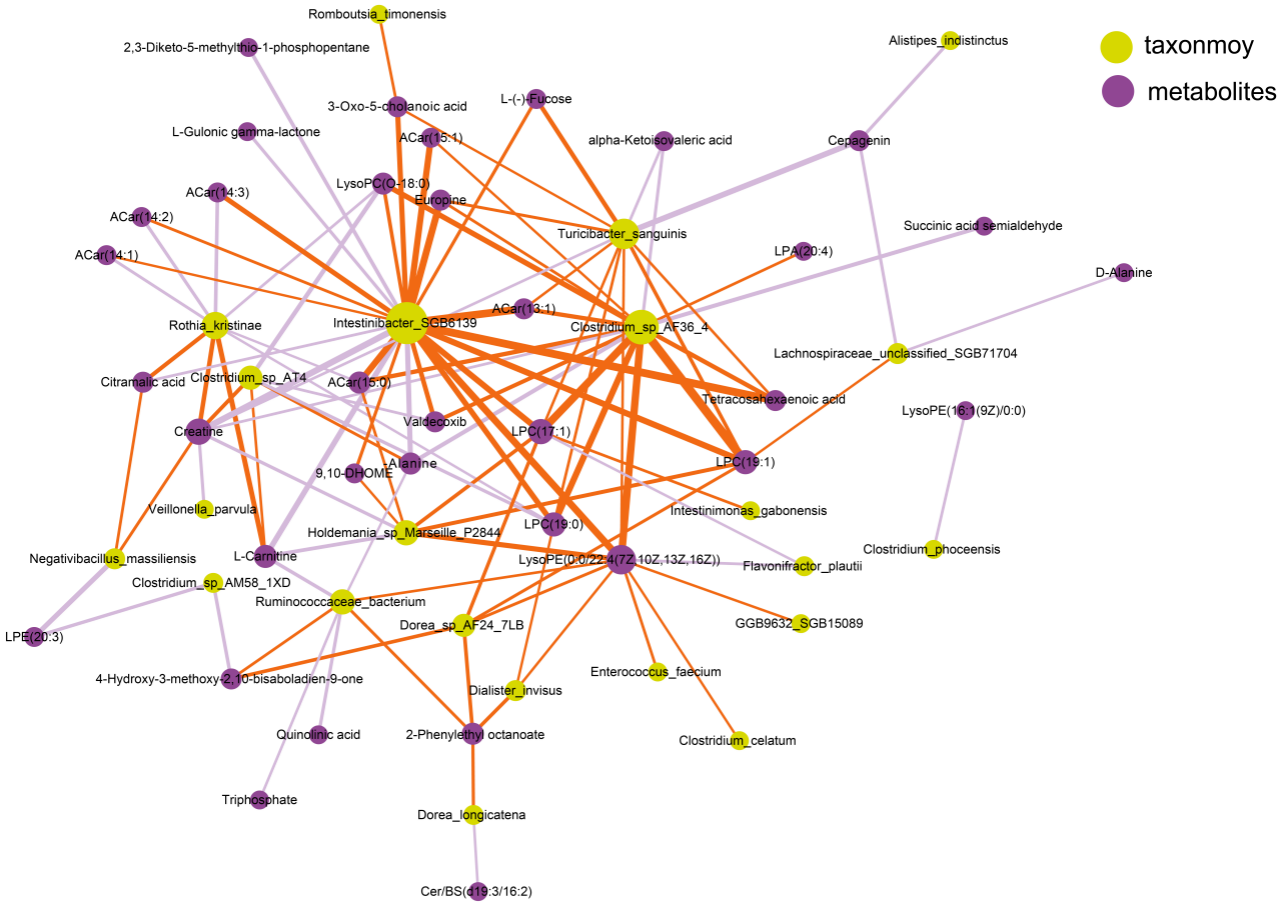


**Figure S5.Analysis of the correlation network between differential microbial communities and metabolites.** The analysis included 120 HC and 120 MAFLD patients, matched for age, sex, etc.


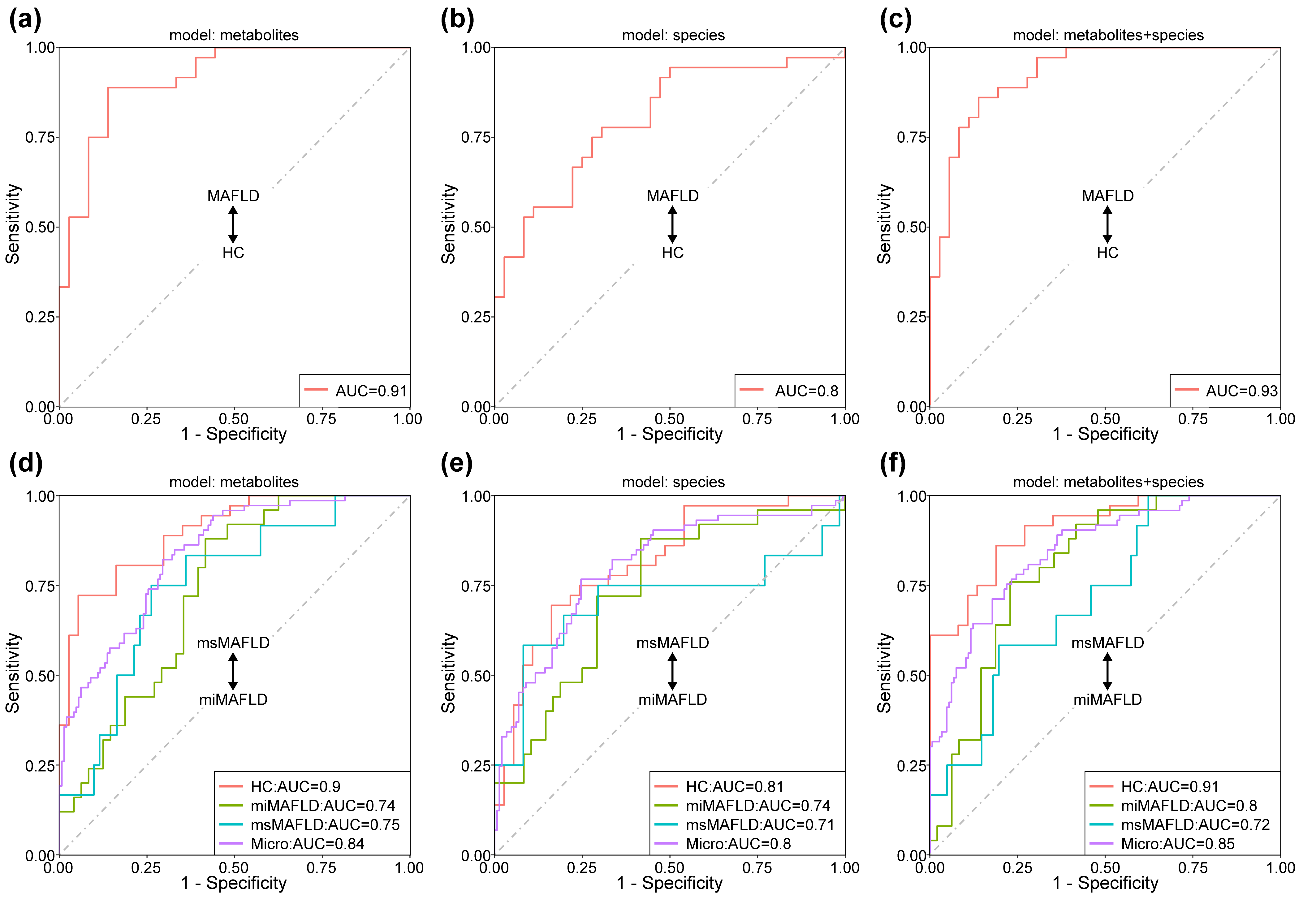


**Figure S6.MAFLD diagnosis and hepatic steatosis prediction model.**(A) MAFLD diagnostic model based on metabolites. (B) MAFLD diagnostic model based on microbiota. (C) MAFLD diagnostic model based on microbiota and metabolites. (D) MAFLD hepatic steatosis prediction model based on Microbial Communities. (E) MAFLD hepatic steatosis prediction model based on metabolite. (F) MAFLD hepatic steatosis prediction model based on microbial communities and metabolites. The analysis included 120 HC and 120 MAFLD patients, matched for age, sex, etc. Out of 120 MAFLD patients, 39 patients with moderate to severe HS (msMAFLD), and 81 patients with mild HS (miMAFLD) were included in overall and subgroup analysis.

**
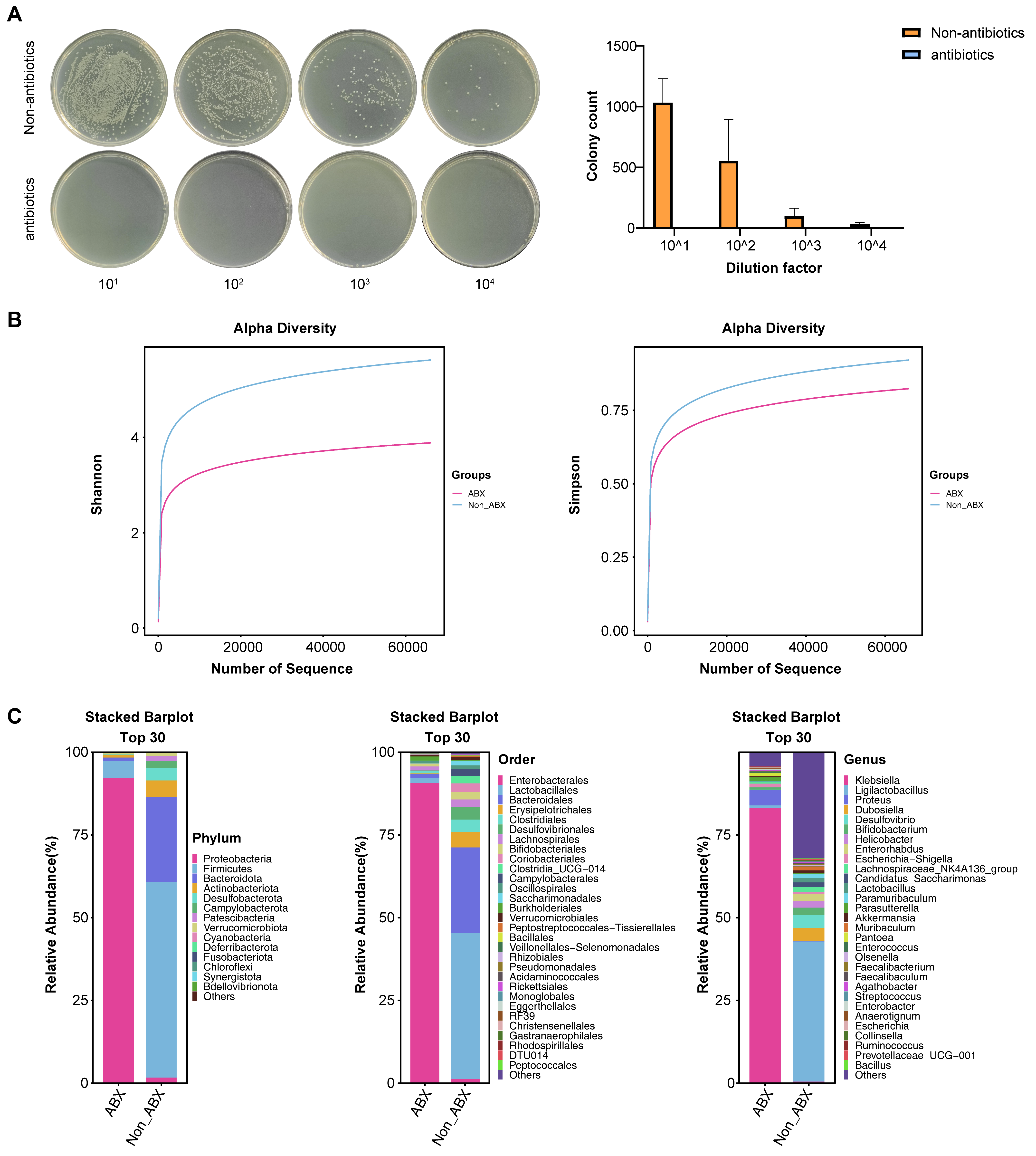
**

**Figure S7. ABX mice was further confirmed by plate coating experiments and 16SrRNA analysis of feces before and after antibiotic induced intestinal clearance in mice.** (A) Fecal coating experiment. (B-C) Fecal 16SrRNA.


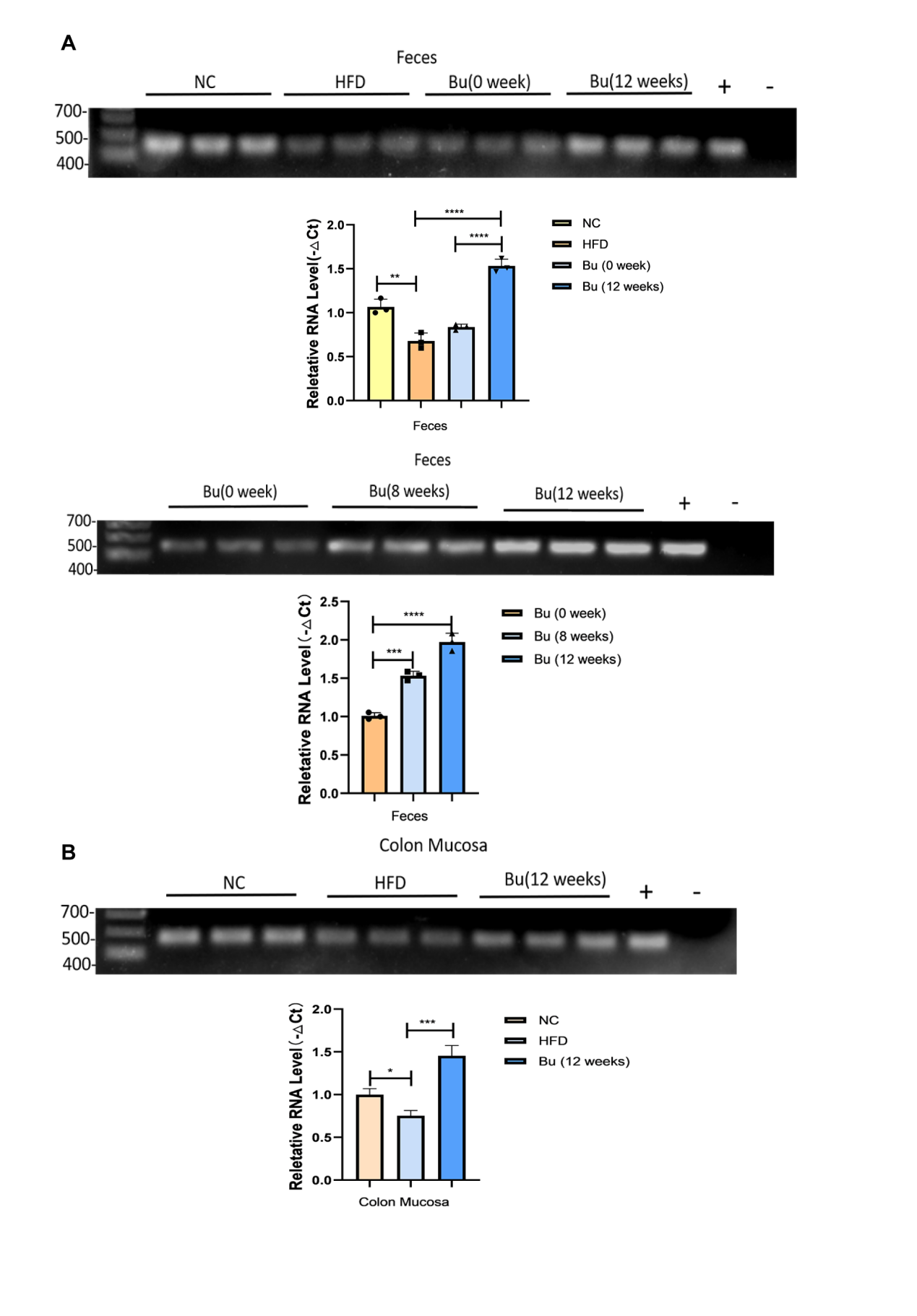


**Figure S8. qPCR analysis of feces and colon mucosa confirming the successful colonization of Bacteroides_uniformis.**(A) qPCR analysis of Bacteroides_uniformis in feces. (B) qPCR analysis of Bacteroides_uniformis in colonic mucosa. n=3 for each group.


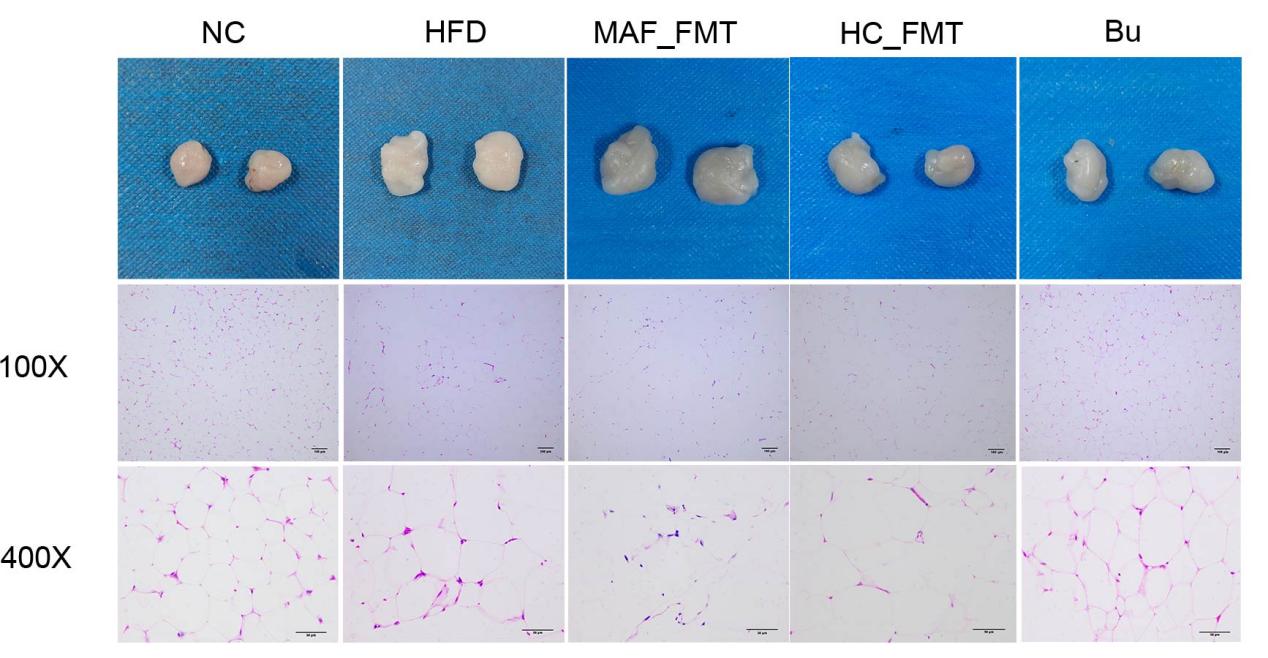


**Figure S9. Effects of FMT and Bu on the pathomorphology of epididymal fat in MAFLD mice.** H&E staining (100×, scale bar 10μm; 400×, scale bar 2.5μm). n=3 for each group.

**
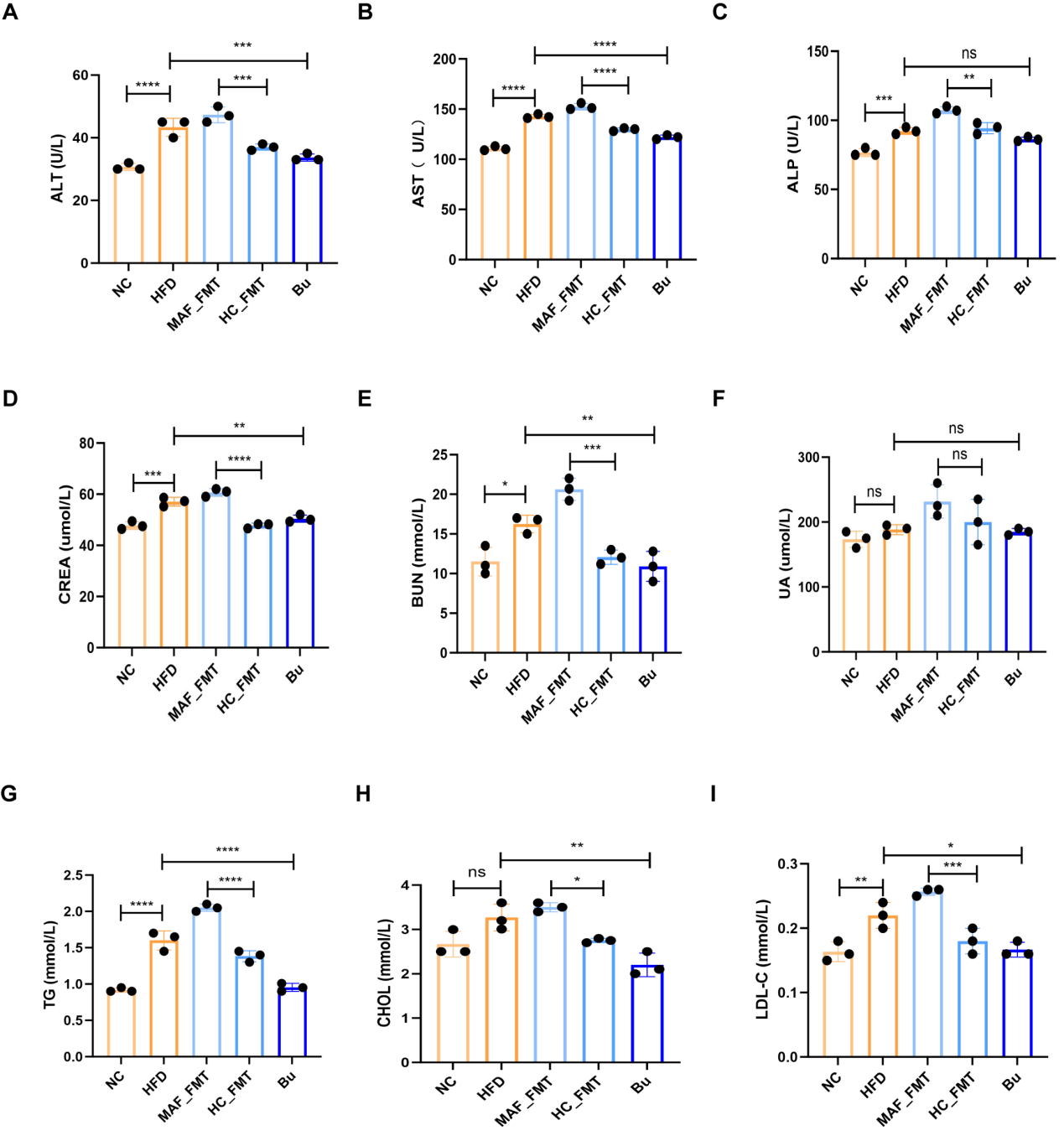
**

**Figure S10. Effects of FMT and Bu on liver damage, kidney function and dyslipidemia in MAFLD mice.** (A) serum ALT, n=3 for each group. (B) serum AST, n=3 for each group. (C) serum ALP, n=3 for each group. (D) serum CREA, n=3 for each group. (E) serum BUN, n=3 for each group. (F) serum UA, n=3 for each group. (G) serum TG, n=3 for each group. (H) serum CHOL, n=3 for each group .(I) serum LDL-C, n=3 for each group (**P*<0.05,***P*<0.01, ****P*<0.001, and *****P*<0.0001, two subgroups were compared separately: NC vs HFD vs Bu, MAF_FMT vs HC_FMT)

**
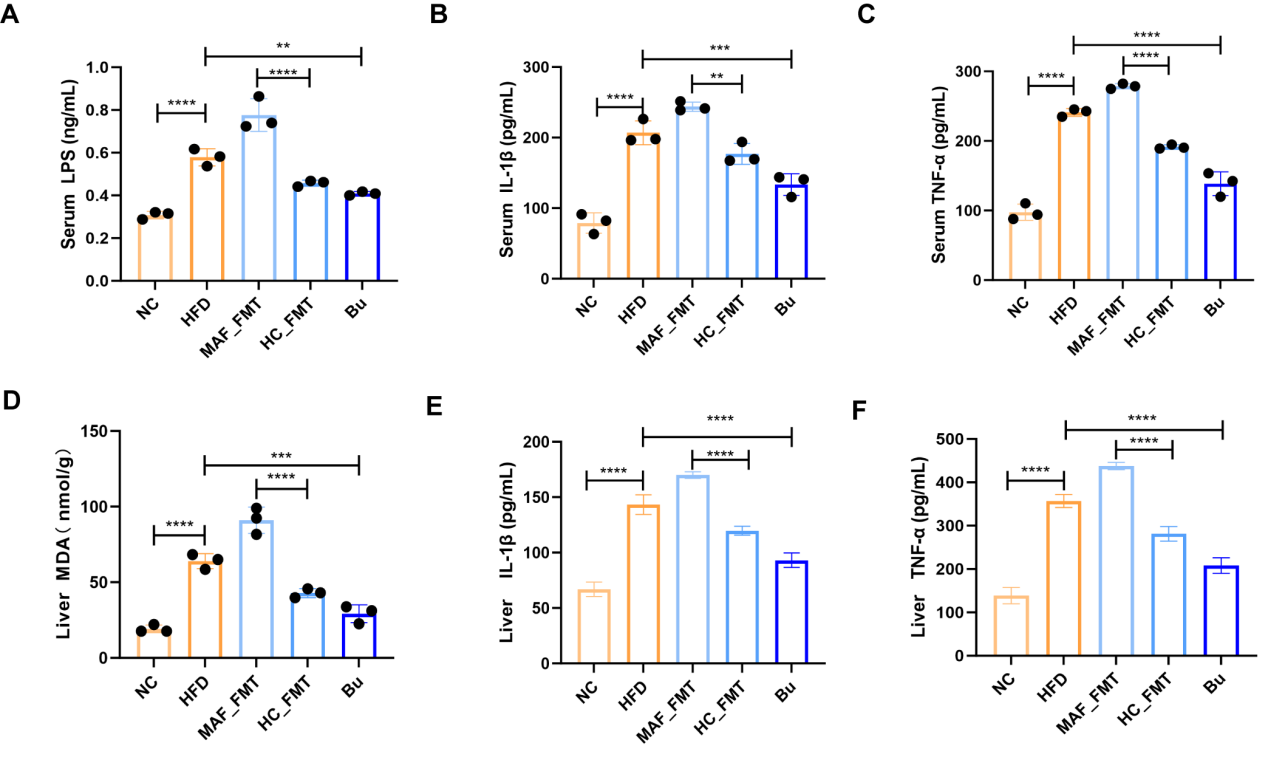
**

**Figure S11.Effects of FMT and Bu on inflammation and oxidative stress in MAFLD mice.** (A) serum LPS, n=3 for each group. (B) serum IL-1β, n=3 for each group. (C) serum TNF-α, n=3 for each group. (D) liver MDA, n=3 for each group. (E) liver IL-1β, n=3 for each group. (F) liver TNF-α, n=3 for each group (**P*<0.05,***P*<0.01, ****P*<0.001, and *****P*<0.0001, two subgroups were compared separately: NC vs HFD vs Bu, MAF_FMT vs HC_FMT)


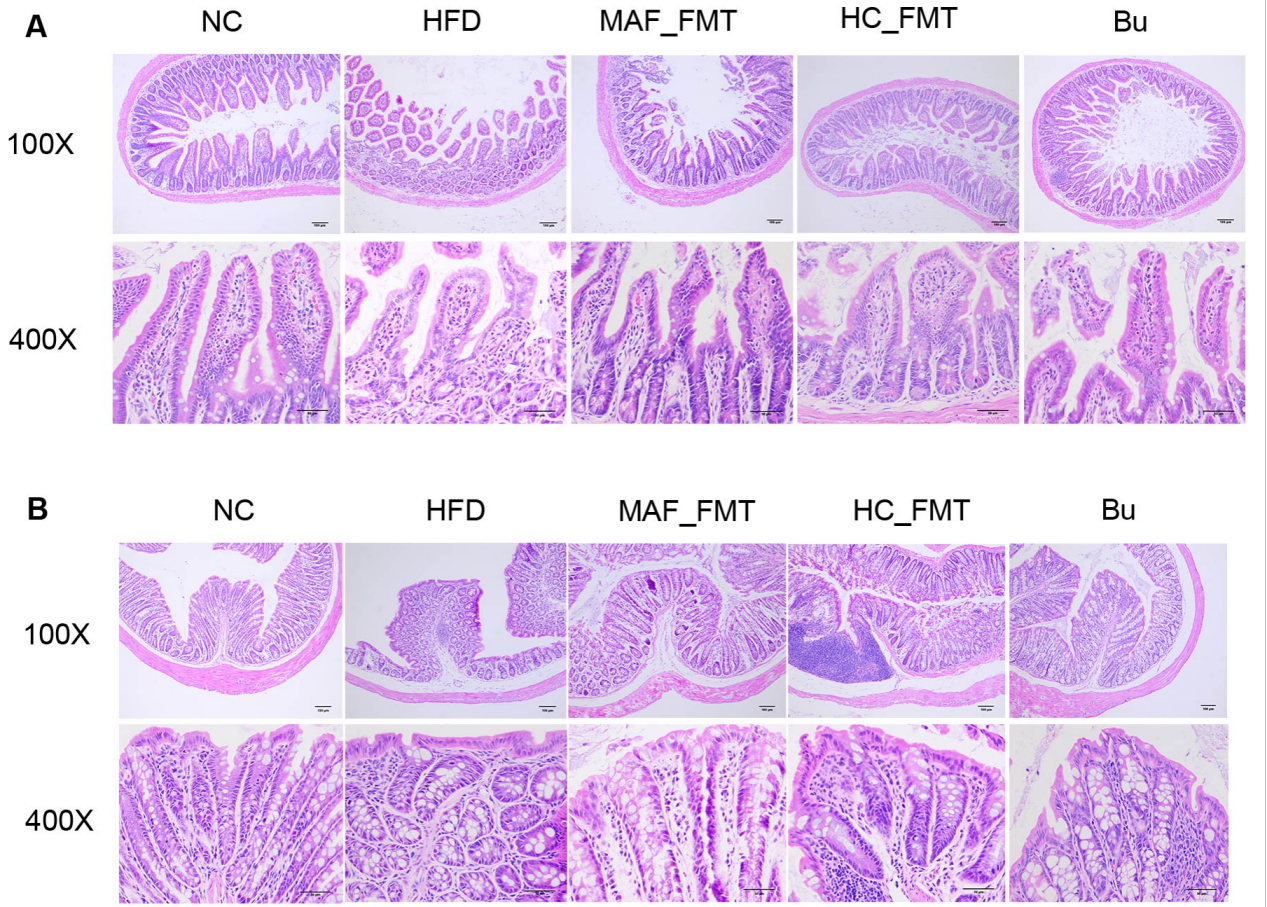


**Figure S12. Effects of FMT and Bu on intestinal mucosal barrier in MAFLD mice.** (A) ileum. (B) colon. H&E staining (100×, scale bar 10μm; 400×, scale bar 2.5μm). n=3 for each group.

**
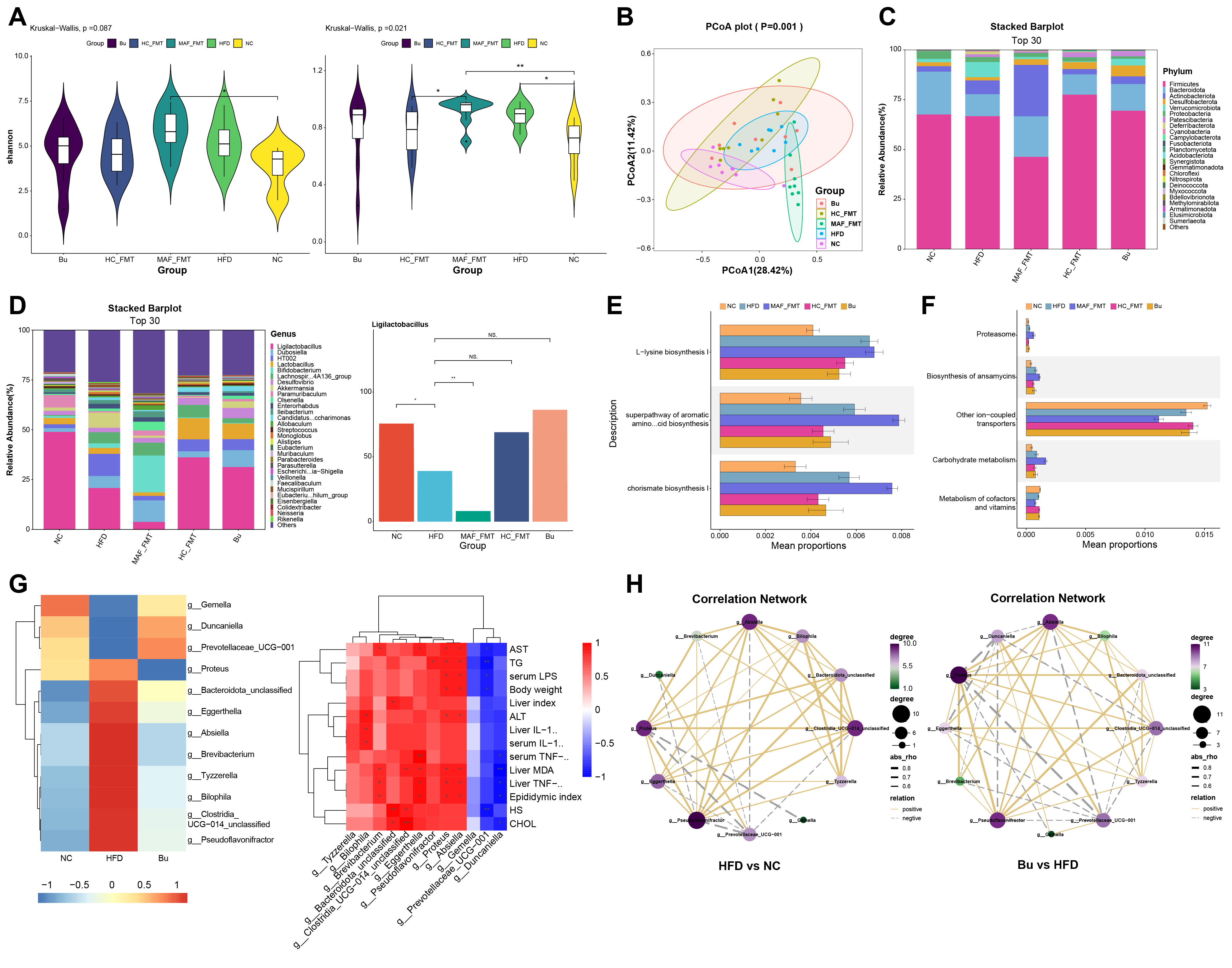
**

**Figure S13. Fecal 16rRNA analysis in different groups.** (A)Alpha Diversity. (B)PCA and PCoA. (C) abundance of intestinal flora at the phylum. (D) abundance of intestinal flora at the genus. (E) PICRUSt2_METACYC. (F)PICRUSt2_KEGG_level3. (G)Analysis of differential genera of Bu reversing MAFLD mice and their association with clinical indicators. (H)Network Interaction Analysis of Differential Bacterial Genera. n=8 for each group.

**
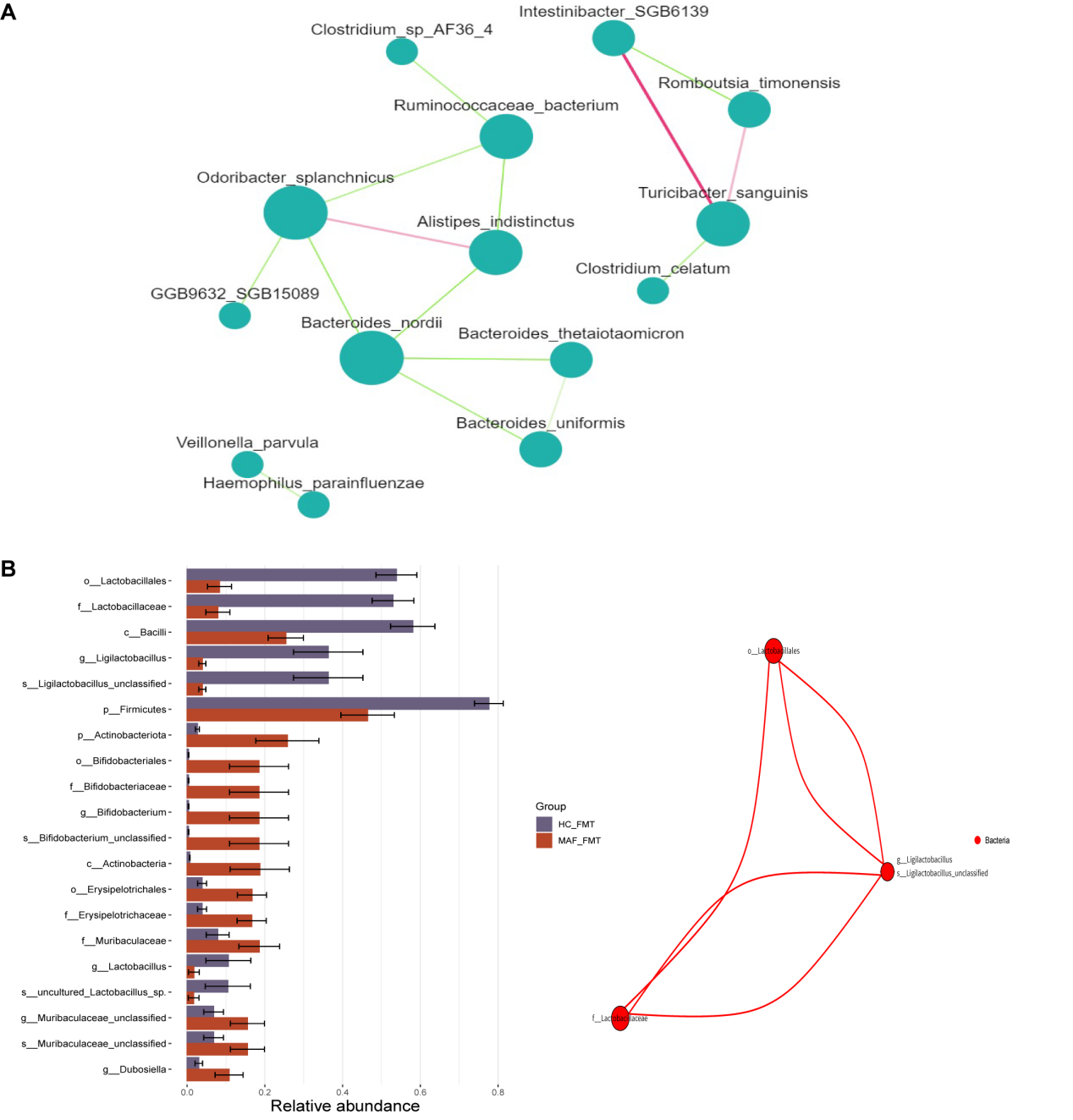
**

**Figure S14. Analysis of the gut microflora of donor populations and recipient mice.** (A)Analysis of bacterial interactions from donor populations fecal macrogenome. (B) Analysis of bacterial interactions from recipient mice fecal 16srRNA.


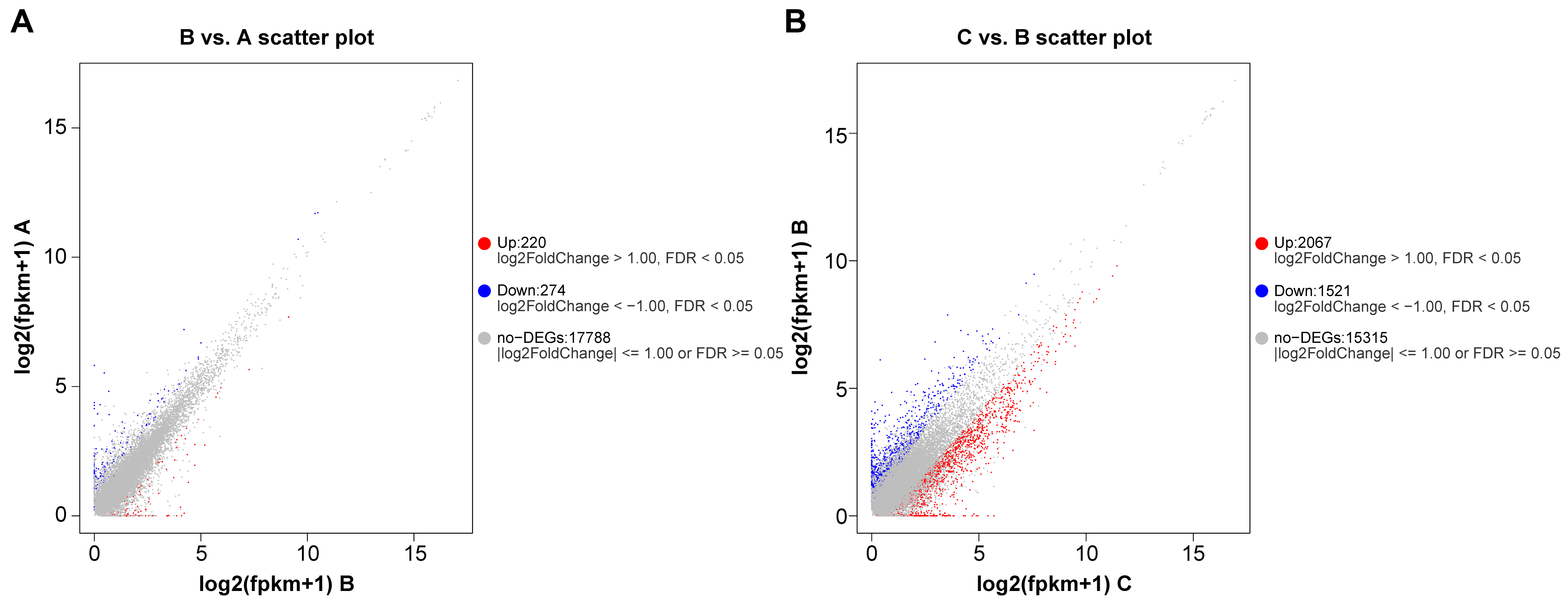


**Figure S15. Volcano plot of gene differences based on mice liver transcriptomics.** (A) HFD vs NC, n=3 for each group. (B) Bu vs HFD, n=3 for each group.


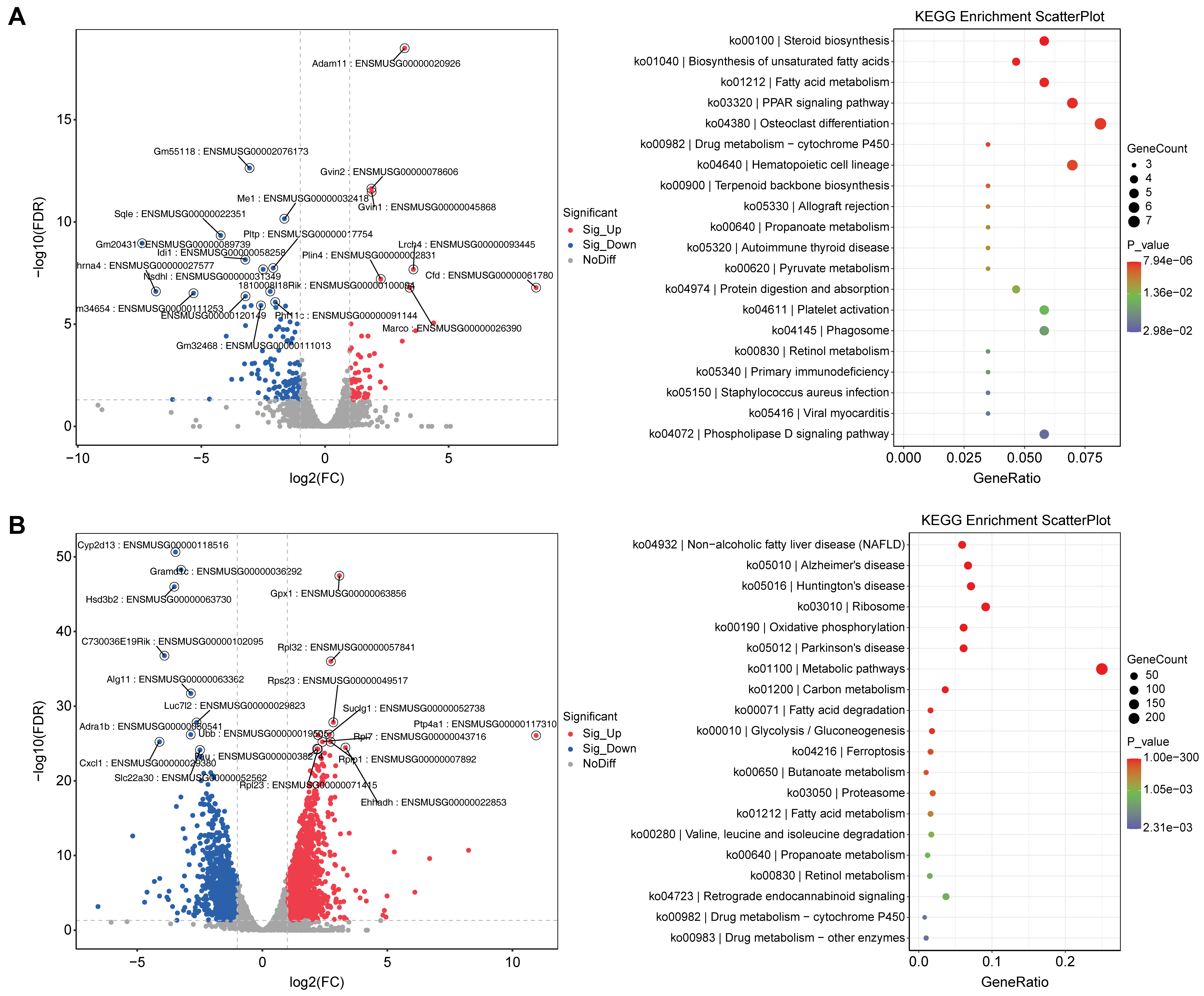


**Figure S16. KEGG pathways and volcano maps for the top 20 genes based on mice liver transcriptomics.** (A) HFD vs NC, n=3 for each group. (B) Bu vs HFD, n=3 for each group.

**
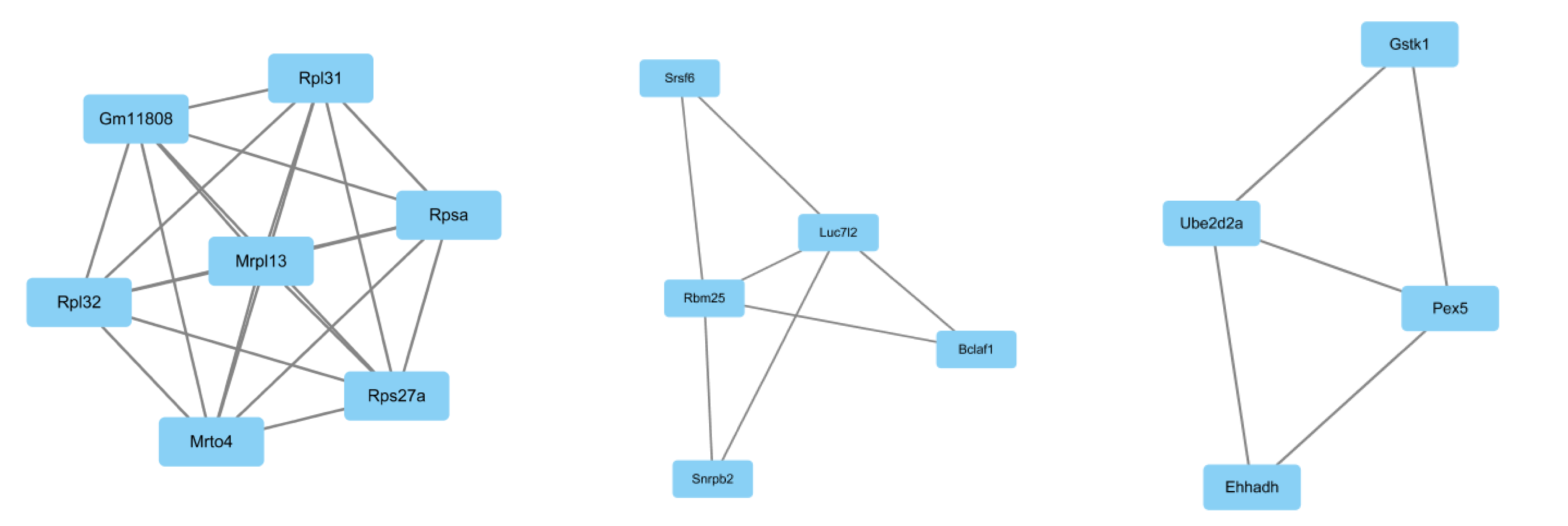
**

**Figure S17. Network diagram of differential gene expression interactions based on mice liver transcriptomics** (Bu vs HFD vs NC, n=3 for each group)


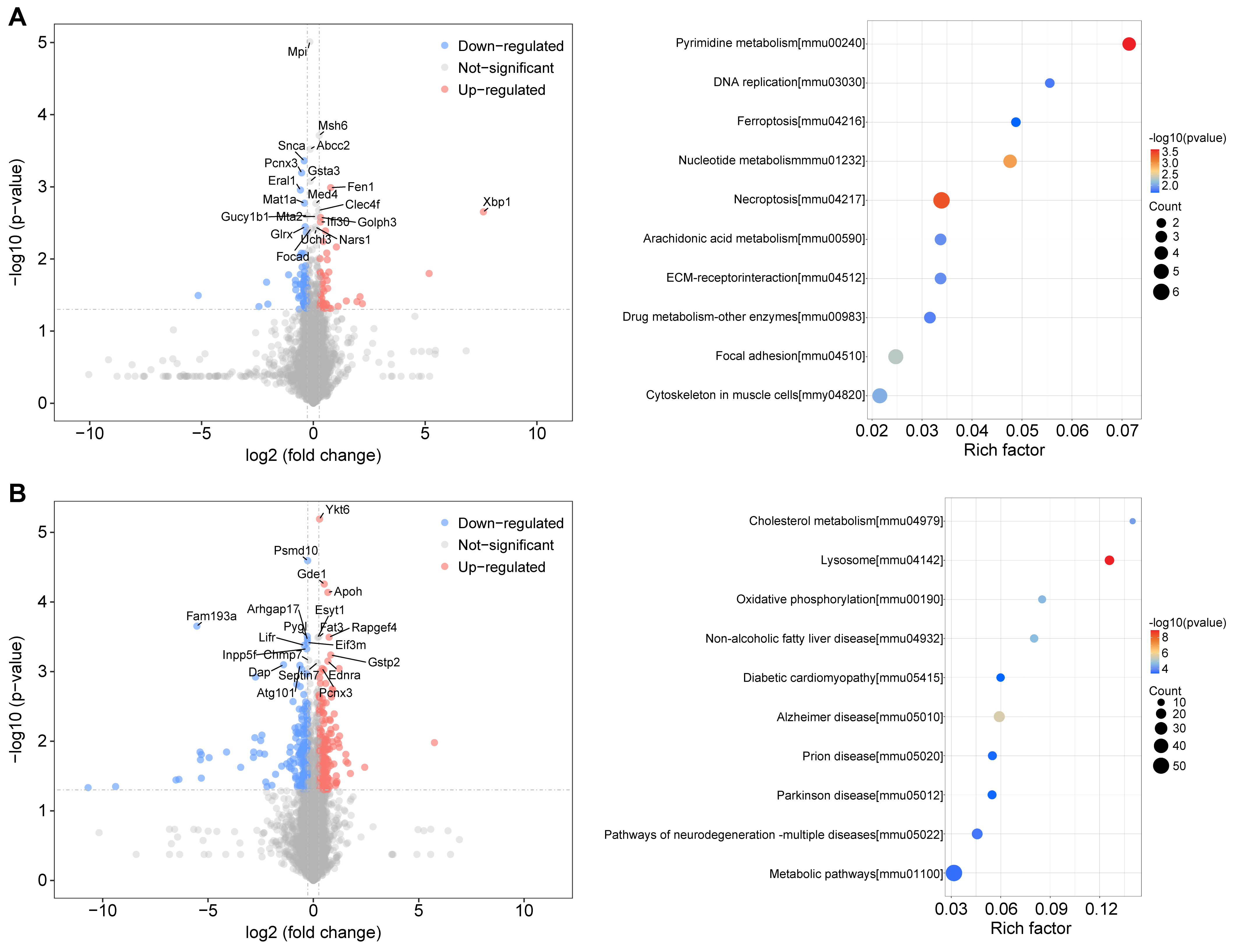


**Figure S18. KEGG pathways and volcano maps for the top 20 genes based on mice liver proteomics.** (A) HFD vs NC, n=3 for each group. (B) Bu vs HFD, n=3 for each group.


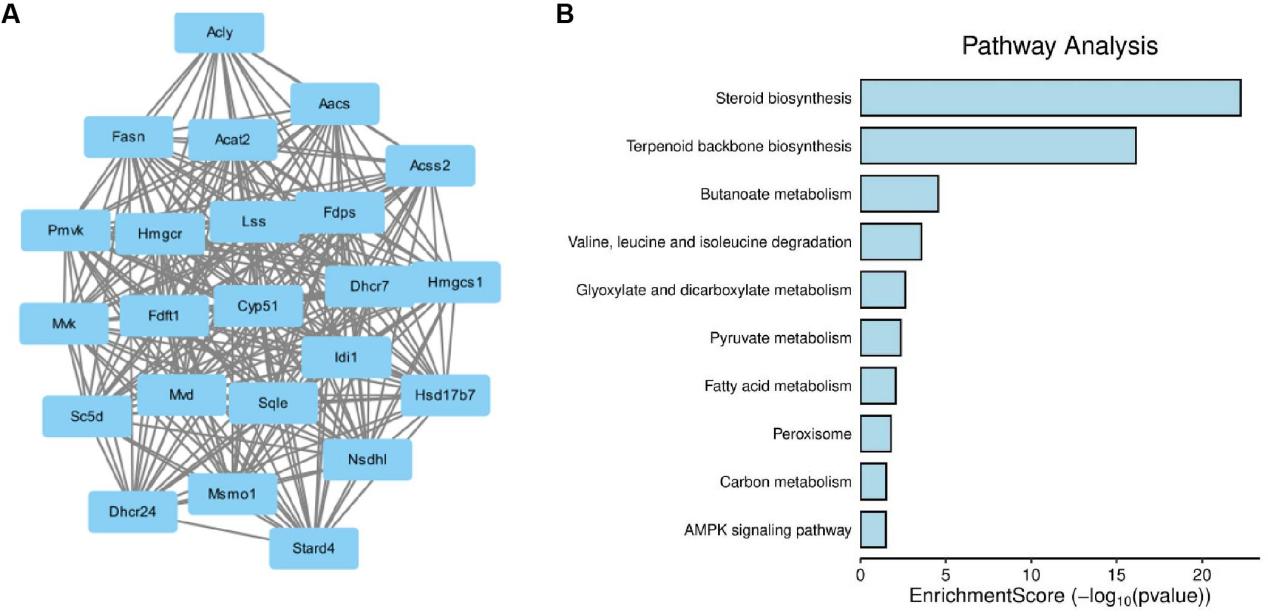


**Figure S19. Network diagram of differential gene expression interactions based on mice liver proteomics** (Bu vs HFD vs NC, n=3 for each group)

**
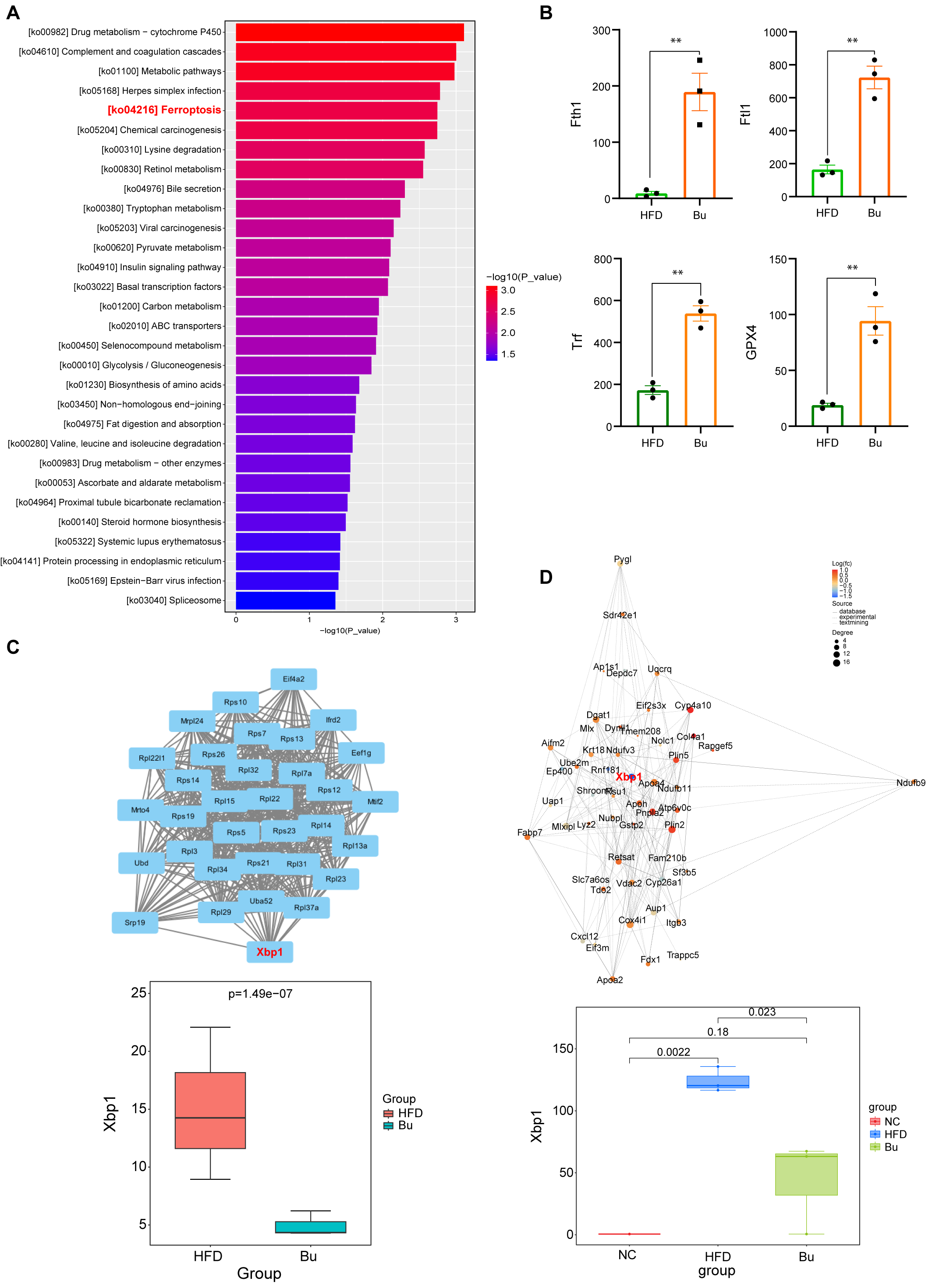
**

**Figure 20. Integrated multi-omics analysis reveals key pathways and molecular networks modulated by Bacteroides uniformis (Bu) intervention in HFD-induced MAFLD mice.** (A) KEGG Enrichment Bar Plot analysis (Y-axis: Names of enriched KEGG pathways, Color gradient: Represents p-value or FDR significance level, Bar length: Indicates enrichment degree or gene count) (Bu vs HFD, n=3 for each group). (B) Differential gene expression profile. Heatmap of significantly altered transcripts (FDR < 0.05, |log2FC| > 1) in Bu-treated vs HFD groups, highlighting key regulatory genes involved in: Iron metabolism (e.g., Fth1, Ftl1, Trf, GPX4) (n=3 for each group). (C) Protein-protein interaction (PPI) network of differentially expressed transcripts(Bu vs HFD, n=3 for each group). (D) Integrated transcriptome-proteome interaction network Overlay analysis identifying: Core hub genes at both mRNA and protein levels (Bu vs HFD, n=3 for each group).


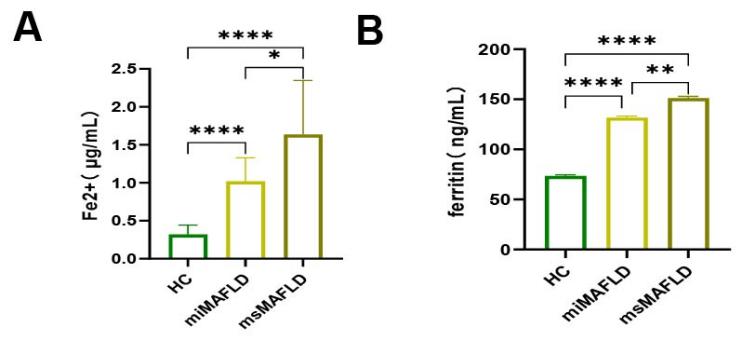


**Figure S21. Serum Fe²⁺and ferritin levels in MAFLD and HC Cohorts.** (A)Serum Fe²⁺. (B) serum ferritin(**p*<0.05,***p*<0.01, ****p*<0.001, and *****p*<0.0001)

**
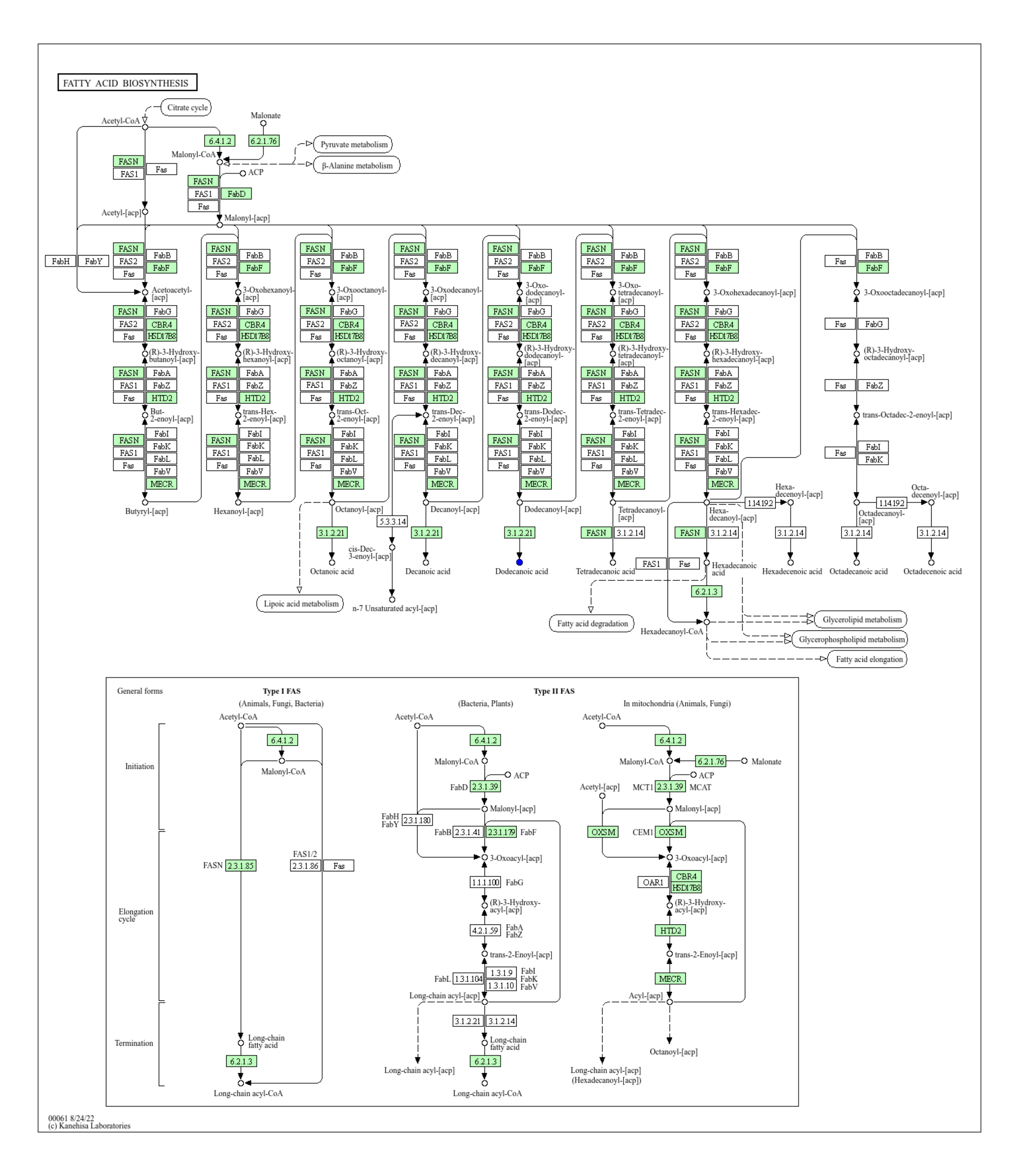
**

**Figure S22.Key pathway for HDA synthesis in Bu:Fatty acid biosynthesis**


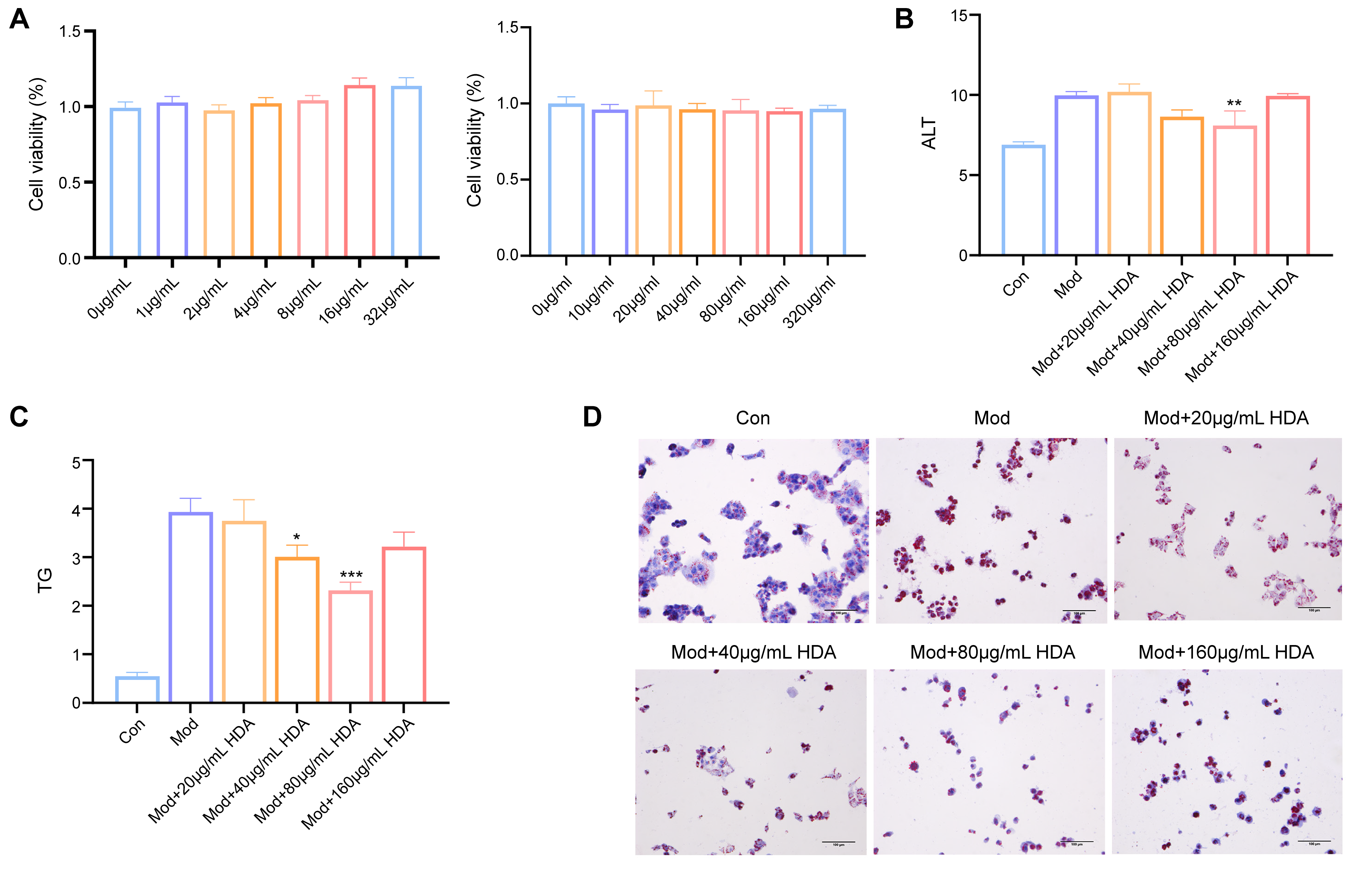


**Figure S23. HDA alleviates lipid deposition and hepatocyte injury in HepG2 cells, an in vitro MAFLD model.** (A) CCK-8 assay. (B) ALT levels in cell samples of different groups. (C) TG levels in cell samples of different groups. (D) results of white light microscopy microscopy after oil red O staining of cells in each group (100×, scale bar 10μm; 400×, scale bar 2.5μm). n=3 for each group. Compared with the Model group, **p*<0.05, ***p*<0.01, ****p*<0.001


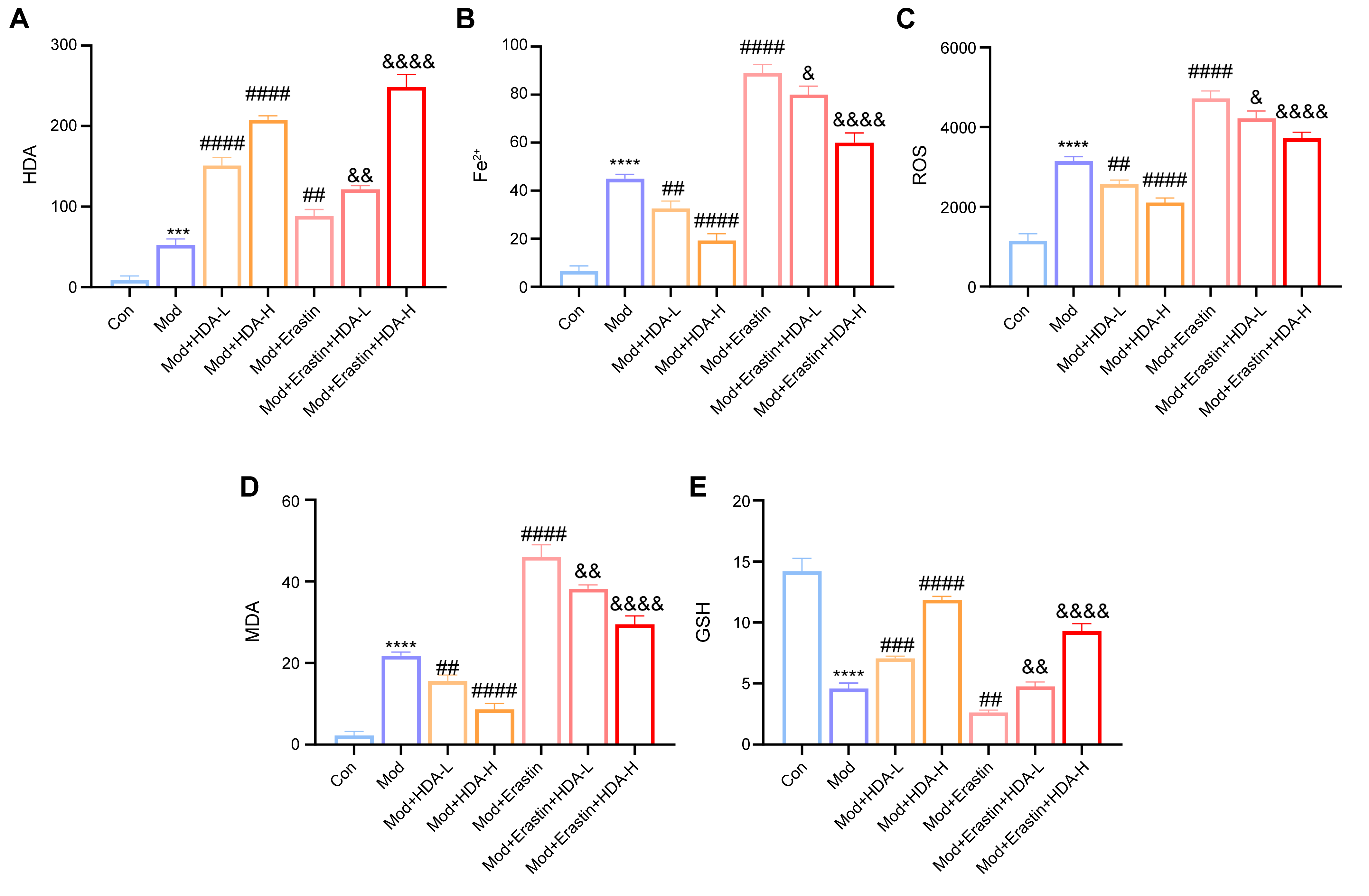


**Figure S24. Effect of HDA o**n **Fe^2^+ metabolism and oxidative stress in MAFLD model of HepG2 cells.** n=3 for each group. Compared with Con group, ****p*<0.001, *****p*<0.0001; compared with Mod group, ##*p*<0.01, ####*p*<0.001, ####*p*<0.0001; compared with Mod+Erastin group, &*p*<0.05, &&*p*<0.01, ＆＆＆＆*p*<0.0001


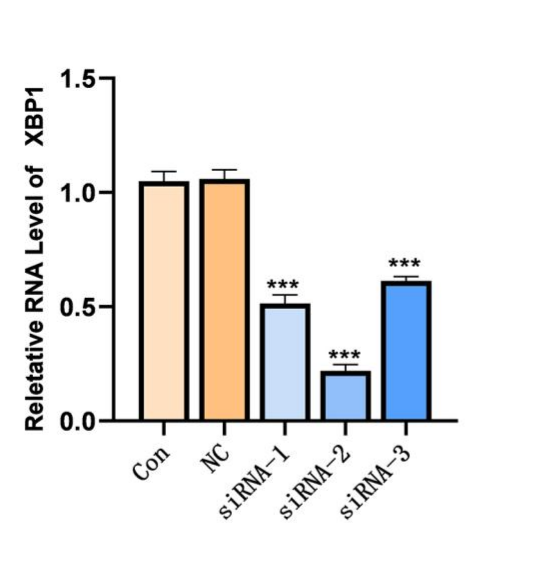


**Figure S25.Transfection efficiency of XBP1 interfering plasmid detected by RT-qPCR.** n=3 for each group. ****p*<0.001 compared with Con


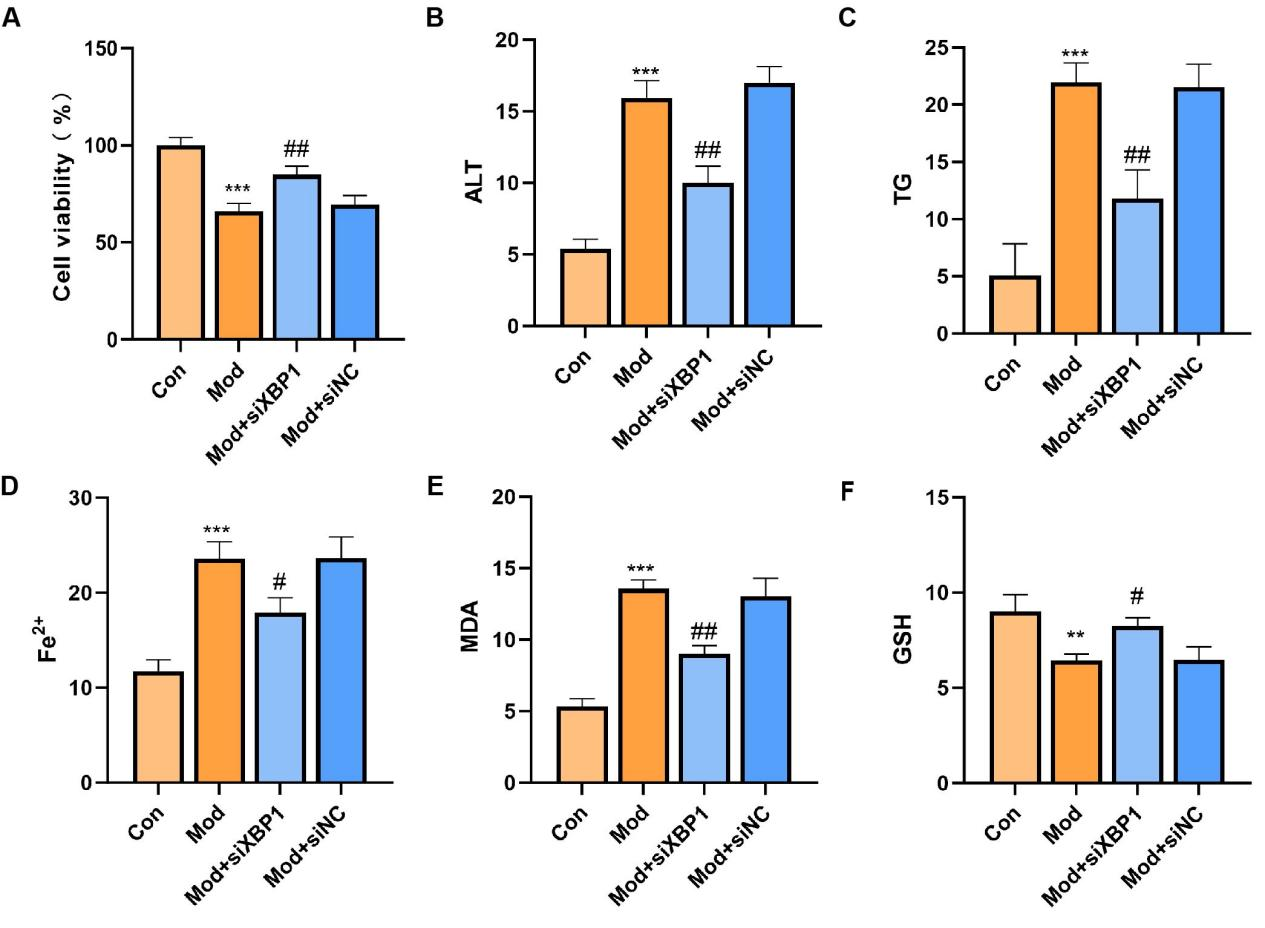


**Figure S26. Effects of knockdown of XBP1 on survival, lipid deposition in cells and damage in HepG2 cells in an in vitro MAFLD model.** (A) Cell survival in different groups. (B) ALT level in cell samples of different groups. (C) TG level in cell samples of different groups. (D) Fe^2^+ level in cell samples of different groups. (E) MDA level in cell samples of different groups. (F) GSH level in cells of each group. n=3 for each group. Compared with Con group, ****p*<0.001; compared with Mod group, ##*p*<0.01


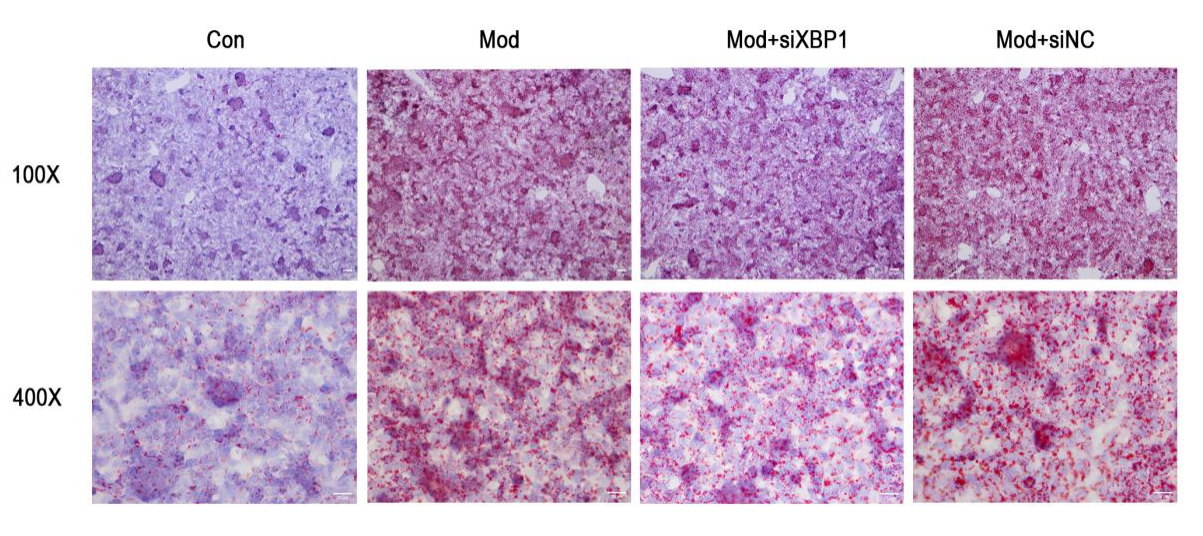


**Figure S27. The results of white light microscopy microscopy after oil red O staining of cell samples of different groups(100×, scale bar 10μm; 400×, scale bar 2.5μm).** n=3 for each group.

**
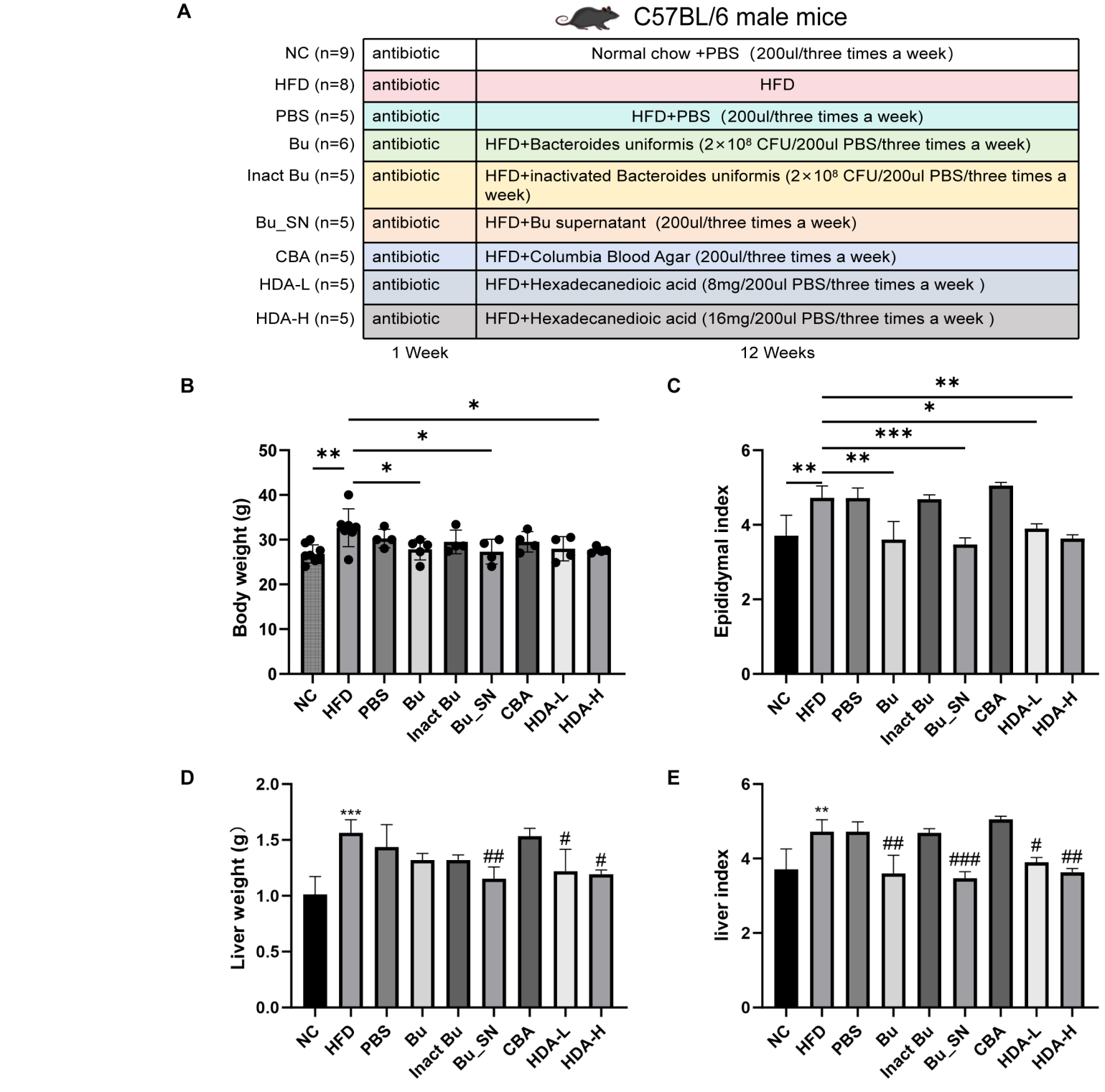
**

**Figure S28. Effects of live Bu, inactivated Bu, Bu bacterial supernatant, culture medium, and exogenous HDA on body weight and organ index of MAFLD mice.** (A) Schematic diagram of animal experimental design. (B) Weight of mice in different groups. (C) white adipose tissue (eWAT) index of mice in different groups. (D) Liver weight of mice in different groups. (E) Liver index of mice in different groups. Compared with the NC group, ****p*<0.001; compared with the PBS group, ##*p*<0.01 and ###*p*<0.001.


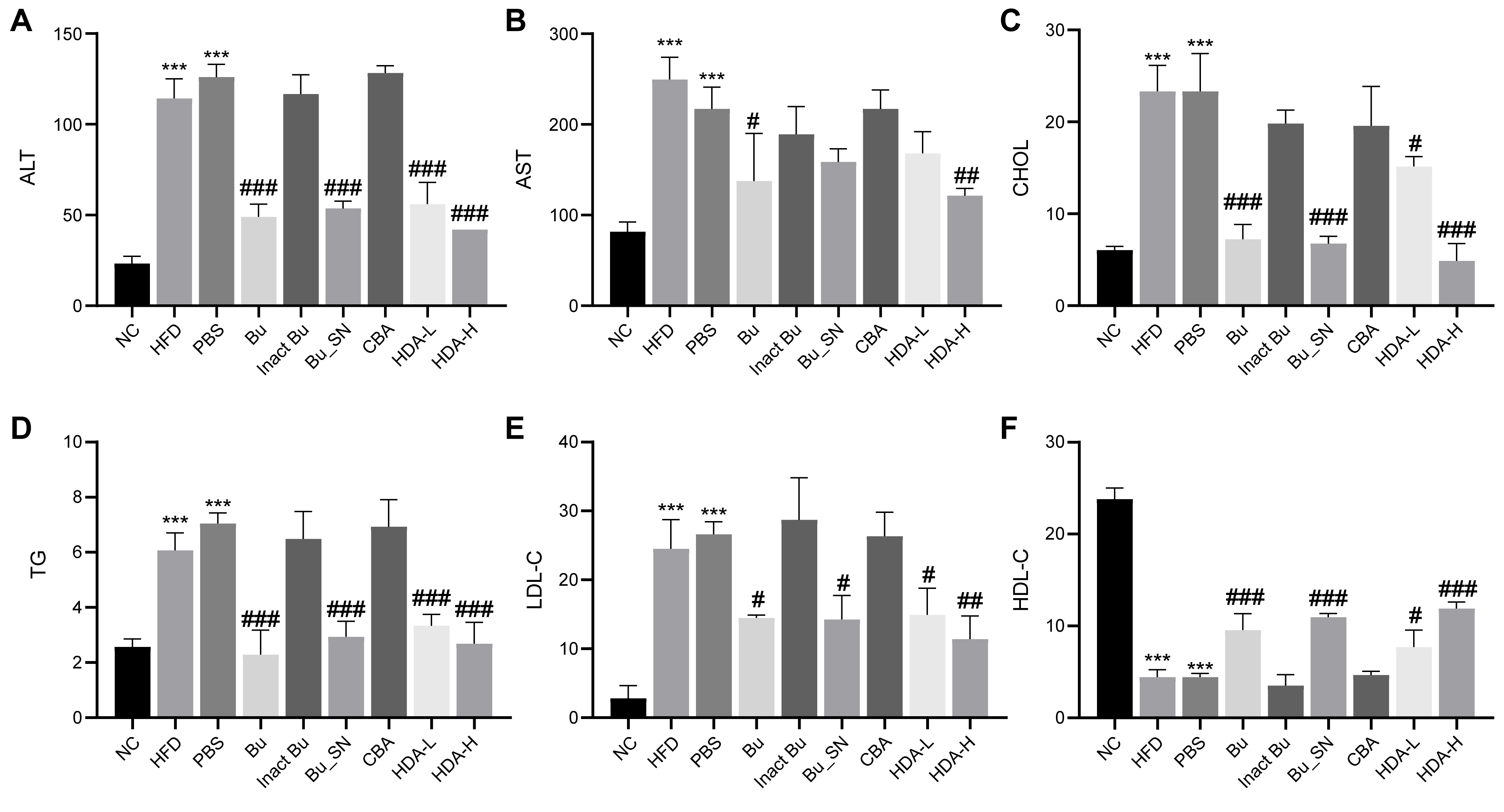


**Figure S29. Effects of live Bu, inactivated Bu, Bu bacterial supernatant, culture medium, and exogenous HDA on Lipids and Liver Function of MAFLD mice.**

(A) ALT. (B) AST. (C)CHOL.(D) TG. (E) LDL-C. (F)HDL-C. n=3 for each group. Compared with NC group, ****p*<0.001; compared with the PBS group, ##*p*<0.01 and ###*p*<0.001.


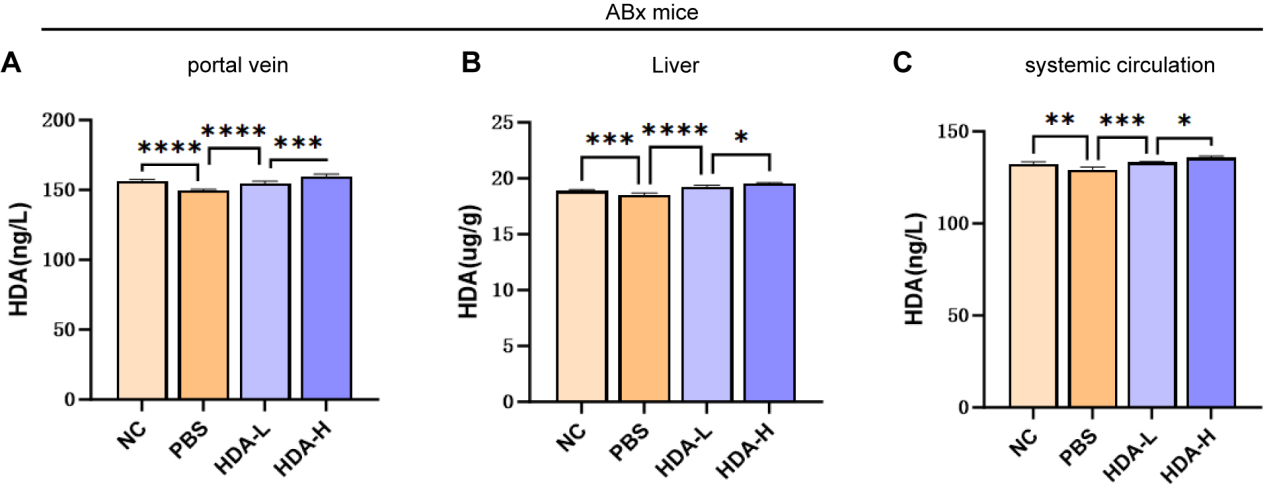


**Figure S30. HDA quantification in the portal vein, liver and systemic circulation of HDA-gavaged and PBS-gavaged germ-free mice.** HDA level in the portal vein(A), Liver(B), and systemic circulation(C) in germ-free mice.


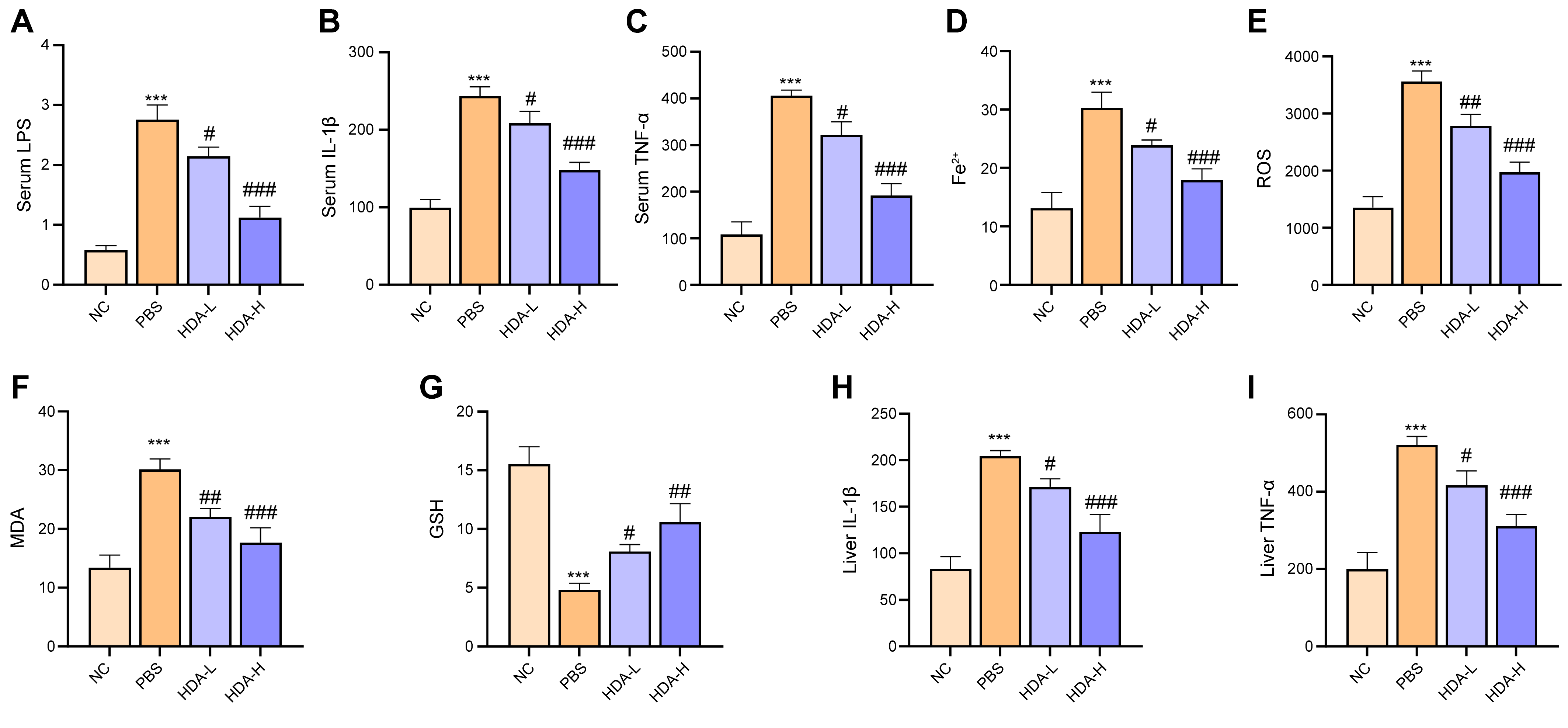


**Figure S31. HDA down-regulated serum endotoxin, serum inflammation and liver tissue Fe2+ metabolism, oxidative stress and inflammation levels in MAFLD mice.** n=3 for each group. Compared with NC group, ****p*<0.001; compared with the PBS group, ##*p*<0.01 and ###*p*<0.001.


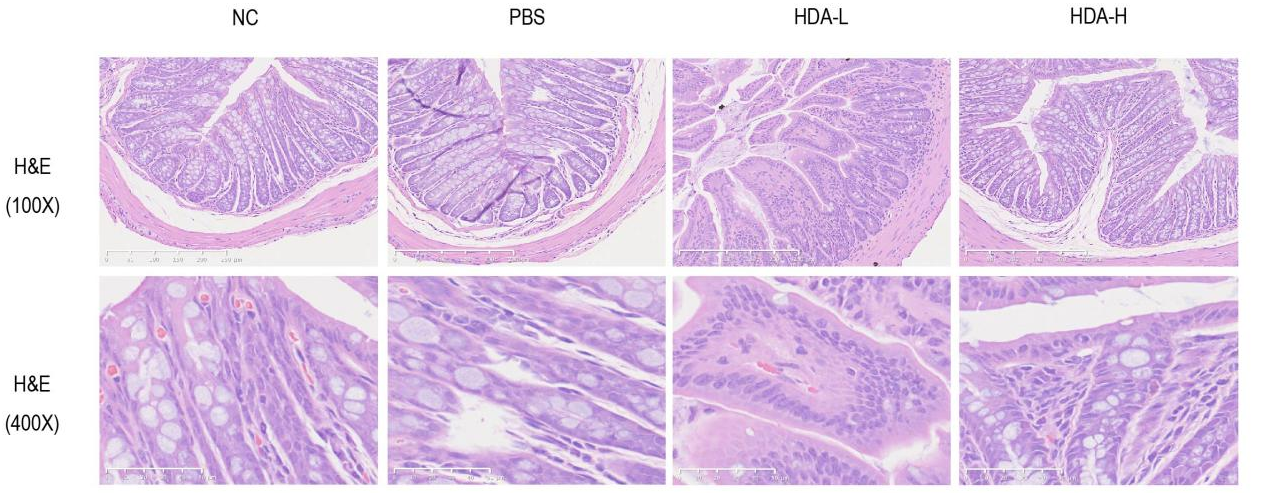


**Figure S32.Effect of HDA on the histopathologic morphology of colon tissue in MAFLD mice.** n=3 for each group. H&E staining of colon tissue (100×, scale bar 10μm; 400×, scale bar 2.5μm).

**Supplementary Tables**

**Table S1. Multifactorial analysis of clinical characteristics of MAFLD patients**

| Indicator | Regression coefficient | SE | Z | *P* | OR | 95%CI |
| --- | --- | --- | --- | --- | --- | --- |
| BMI (kg/cm2) | 0.206 | 0.056 | 13.502 | <0.001 | 1.229 | 1.101-1.372 |
| ALT (IU/L) | 0.108 | 0.030 | 13.365 | <0.001 | 1.114 | 1.052-1.181 |
| AST (IU/L) | -0.089 | 0.044 | 4.127 | 0.042 | 0.915 | 0.840-0.997 |
| GGT (IU/L) | -0.008 | 0.010 | 0.681 | 0.409 | 0.992 | 0.973-1.011 |
| FPG (mmol/L) | 0.553 | 0.224 | 6.086 | 0.014 | 1.739 | 1.120-2.699 |
| URIC (umol/L) | 0.009 | 0.003 | 9.245 | 0.002 | 1.009 | 1.003-1.016 |
| TG (mmol/L) | 1.053 | 0.393 | 7.174 | 0.007 | 2.867 | 1.326-6.195 |
| CHOL (mmol/L) | 0.010 | 0.248 | 0.002 | 0.969 | 1.010 | 0.622-1.640 |
| HDL-C (mmol/L) | -0.454 | 0.565 | 0.648 | 0.421 | 0.635 | 0.210-1.920 |
| LDL-C (mmol/L) | -0.069 | 0.106 | 0.417 | 0.519 | 0.934 | 0.758-1.150 |

**Table S2. Clinical characteristics of patients with different steatosis MAFLD**

| Indicator | HC (n = 120) | miMAFLD (n = 81) | msMAFLD (n = 39) | Test value | *p* |
| --- | --- | --- | --- | --- | --- |
| Age (year) | 48.00(41-55.00) | 52.0(46-56) | 52.00(43.5-57.00) | 3.745 | 0.154 |
| Gender [male], n [%] | 41(34.17%) | 29(35.80%) | 20(51.28%) | 3.829 | 0.146 |
| BMI (kg/cm^2^) | 22.87(20.95-24.46) | 26.29(23.26-29.29) | 27.25(24.97-29.69) | 54.455 | <0.001 |
| ALT (IU/L) | 16.5(13-21) | 23(16-31.5) | 34(22-50) | 52.092 | <0.001 |
| AST (IU/L) | 20(17-24) | 21(17.5-26.00) | 24(20-29) | 12.757 | 0.002 |
| GGT (IU/L) | 20(14.25-28) | 29(21-50.5) | 41.0(23-55) | 42.932 | <0.001 |
| FPG (mmol/L) | 4.925(4.2875-5.185) | 5.07(4.725-6.165) | 5.28(5.0-7.0) | 22.930 | <0.001 |
| URIC (umol/L) | 297.44±57.02 | 373.14±84.54 | 361.74±76.11 | 31.608 | <0.001 |
| TG (mmol/L) | 1.000(0.7525-1.0875) | 1.78(1.09-2.20) | 1.56(1.000-2.69) | 58.767 | <0.001 |
| CHOL (mmol/L) | 5.0(4.3725-5.4425) | 5.14(4.725-6.000) | 5.25(4.93-6.0) | 9.045 | 0.011 |
| HDL-C (mmol/L) | 1.615(1.3125-1.9975) | 1.22(1.000-1.495) | 1.00(1.000-1.41) | 49.58 | <0.001 |
| LDL-C (mmol/L) | 2.905(2.415-3.205) | 3.31(2.64-3.815) | 3.1(2.69-4.000) | 14.726 | 0.001 |

| Indicator | HC vs miMAFLD | HC vs msMAFLD | miMAFLD vs msMAFLD |
| --- | --- | --- | --- |
| BMI (kg/cm^2^) | <0.001 | <0.001 | 0.110 |
| ALT (IU/L) | <0.001 | <0.001 | 0.001 |
| AST(IU/L) | 0.417 | <0.001 | 0.007 |
| GGT(IU/L) | <0.001 | <0.001 | 0.050 |
| FPG (mmol/L) | <0.001 | <0.001 | 0.117 |
| URIC (umol/L) | <0.001 | <0.001 | 0.476 |
| TG (mmol/L) | <0.001 | <0.001 | 0.745 |
| CHOL (mmol/L) | 0.022 | 0.010 | 0.519 |
| HDL-C (mmol/L) | <0.001 | <0.001 | 0.142 |
| LDL-C (mmol/L) | 0.001 | 0.004 | 0.732 |

**Table S3. Bacterial and metabolite association analysis**

| node1 | node2 | corr | corr.p |
| --- | --- | --- | --- |
| LPC(19:1) | Clostridium_sp_AF36_4 | 0.434918613 | 1.70216E-12 |
| Tetracosahexaenoic acid | Intestinibacter_SGB6139 | 0.410381655 | 3.63493E-11 |
| ACar(13:1) | Intestinibacter_SGB6139 | 0.405844095 | 6.23402E-11 |
| LPC(17:1) | Clostridium_sp_AF36_4 | 0.402581633 | 9.14195E-11 |
| β-Alanine | Clostridium_sp_AF36_4 | -0.30153961 | 1.95268E-06 |
| LysoPC(O-18:0) | Clostridium_sp_AT4 | -0.310211814 | 9.47883E-07 |
| LPE(20:3) | Negativibacillus_massiliensis | -0.325877374 | 2.42036E-07 |
| β-Alanine | Intestinibacter_SGB6139 | -0.32911734 | 1.80725E-07 |
| L-Carnitine | Intestinibacter_SGB6139 | -0.341303753 | 5.84164E-08 |
| Cepagenin | Turicibacter_sanguinis | -0.344766292 | 4.20027E-08 |
| Creatine | Intestinibacter_SGB6139 | -0.363361078 | 6.66289E-09 |

**Table S4. food consumption in five groups（g/mouse/day）**

| Time | NC | HFD | MAF-FMT | HC-FMT | Bu | *P* |
| --- | --- | --- | --- | --- | --- | --- |
| 2 weeks | 4.34±0.26 | 4.45±0.33 | 4.17±0.10 | 4.41±0.43 | 4.48±0.38 | 0.4307 |
| 4 weeks | 4.80±0.28 | 5.01±0.41 | 4.78±0.42 | 4.54±0.56 | 4.42±0.46 | 0.1346 |
| 6 weeks | 4.66±0.19 | 4.72±0.19 | 4.54±0.22 | 4.61±0.19 | 4.64±0.36 | 0.4567 |
| 8 weeks | 4.65±0.33 | 4.68±0.40 | 4.40±0.34 | 4.81±0.27 | 4.72±0.55 | 0.3759 |
| 10 weeks | 4.65±0.45 | 4.61±0.38 | 4.62±0.35 | 4.80±0.34 | 5.04±0.21 | 0.1572 |
| 12 weeks | 4.66±0.19 | 4.72±0.19 | 4.54±0.22 | 4.61±0.19 | 4.64±0.36 | 0.3821 |

**Table S5. food consumption nine groups（g/mouse/day）**

| Time | 2 | 4 | 6 | 8 | 10 | 12 |
| --- | --- | --- | --- | --- | --- | --- |
| NC | 4.38±0.31 | 4.72±0.40 | 5.00±0.44 | 4.60±0.36 | 4.87±0.27 | 4.52±0.46 |
| HFD | 4.45±0.31 | 5.00±0.39 | 4.84±0.43 | 4.68±0.42 | 4.78±0.17 | 4.72±0.17 |
| PBS | 4.17±0.12 | 4.90±0.48 | 4.67±0.45 | 4.45±0.41 | 4.82±0.26 | 4.37±0.56 |
| Bu | 4.58±0.40 | 4.62±0.60 | 4.62±0.25 | 4.64±0.16 | 4.98±0.25 | 4.58±0.43 |
| Inact Bu | 4.47±0.55 | 4.55±0.61 | 4.57±0.26 | 4.97±0.54 | 5.10±0.24 | 4.75±0.36 |
| Bu_SN | 4.67±0.41 | 4.50±0.37 | 4.82±0.15 | 4.77±0.61 | 4.85±0.17 | 5.02±0.45 |
| CBA | 4.70±0.54 | 4.35±0.23 | 4.55±0.12 | 4.70±0.25 | 4.75±0.12 | 4.47±0.42 |
| HDA-L | 4.30±0.31 | 4.30±0.21 | 4.52±0.53 | 4.50±0.47 | 5.17±0.17 | 4.65±0.50 |
| HDA-H | 4.42±0.47 | 4.82±0.30 | 4.80±0.31 | 4.32±0.32 | 4.72±0.15 | 4.85±0.40 |
| P | 0.6140 | 0.1836 | 0.4151 | 0.5443 | 0.0547 | 0.4778 |
